# Strategies in the delivery of Cas9 ribonucleoprotein for CRISPR/Cas9 genome editing

**DOI:** 10.7150/thno.47007

**Published:** 2021-01-01

**Authors:** Song Zhang, Jiangtao Shen, Dali Li, Yiyun Cheng

**Affiliations:** 1 South China Advanced Institute for Soft Matter Science and Technology, School of Molecular Science and Engineering, South China University of Technology, Guangzhou 510640, China.; 2 The Second People's Hospital of Taizhou affiliated to Yangzhou University, Taizhou, 225500, China.; 3 Shanghai Key Laboratory of Regulatory Biology, East China Normal University, Shanghai 200241, China.

**Keywords:** genome editing, CRISPR, RNP, polymers, nanoparticles

## Abstract

CRISPR/Cas9 genome editing has gained rapidly increasing attentions in recent years, however, the translation of this biotechnology into therapy has been hindered by efficient delivery of CRISPR/Cas9 materials into target cells. Direct delivery of CRISPR/Cas9 system as a ribonucleoprotein (RNP) complex consisting of Cas9 protein and single guide RNA (sgRNA) has emerged as a powerful and widespread method for genome editing due to its advantages of transient genome editing and reduced off-target effects. In this review, we summarized the current Cas9 RNP delivery systems including physical approaches and synthetic carriers. The mechanisms and beneficial roles of these strategies in intracellular Cas9 RNP delivery were reviewed. Examples in the development of stimuli-responsive and targeted carriers for RNP delivery are highlighted. Finally, the challenges of current Cas9 RNP delivery systems and perspectives in rational design of next generation materials for this promising field will be discussed.

## Introduction

The clustered regularly interspaced short palindromic repeat (CRISPR)/CRISPR-associated (Cas) system as a revolutionary genome editing technology offers a powerful tool for scientific research and has great potential in the treatment of various diseases 1, 2. To date, more than 3000 human genes have been identified to be associated with genetic disorders and over 500 genes are related with susceptibility to complex diseases or infections 3. Therefore, genome editing tools like the CRISPR/Cas system have gained great attentions over the past decade for the well promise of treating genetic disorders 4-8. The native CRISPR/Cas system is an adaptive immune system in bacteria and archaea that protects themselves from invasive nucleic acids. Nakata and co-workers first reported a set of highly homologous sequence in the 3' end of *E. coli iap* gene in 1987 9. In the following years, studies have shown that similar repeating sequences exist in a variety of bacteria and archaea, and then the acronym CRISPR was first proposed by Jansen et al. in 2002 10. In 2005, Mojica et al. found that virus cannot infect cells possessing interval sequence with homology to the virus, so they speculated that the CRISPR and associated proteins might participate in the immune function against transmissible genetic elements 11, 12. Following these initial studies, a rapid increase of investigations have revealed more details of the characteristics and mechanisms on CRISPR system 13. In 2012, Doudna, Charpentier and colleagues first reported that the CRISPR-associate protein 9 can be adapted to genome editing with a customized CRISPR RNA (crRNA) together with a common transactivating CRISPR RNA (tracrRNA) or an artificial single guide RNA (sgRNA) which was a chimeric crRNA-tracrRNA hybrid 14. The CRISPR/Cas9 system has been further proven to be effective for genome editing in eukaryotic cells for the first time in 2013 15, 16. Subsequently, a flurry of papers proved the efficacy of CRISPR/Cas9 on genome editing in various species 17-24.

CRISPR/Cas systems can be divided into two classes by the difference of Cas proteins content. The class 1 system needs multiple Cas proteins to form the CRISPR-associated complex for antiviral defense (CASCADE), while the class 2 system only relies on a single Cas protein with multiple domains. The class 1 system contains type I, III and IV and the class 2 system contains type II and type V. Each type of CRISPR/Cas system possesses a distinctive composition of expression, interference, and adaptation modules. They are distinguishable by the presence of unique signature proteins: Cas3 for type I, Cas9 for type II, Cas10 for type III, Csf1 for type IV and Cas12a (Cpf1) for Type V 25. Among these different types of CRISPR/Cas systems, the type II system based on CRISPR/Cas9 from *streptococcus pyogenes* is the most widely studied and applied due to its simplicity, versatility, efficiency and specificity 26-29.

**Figure 1:**
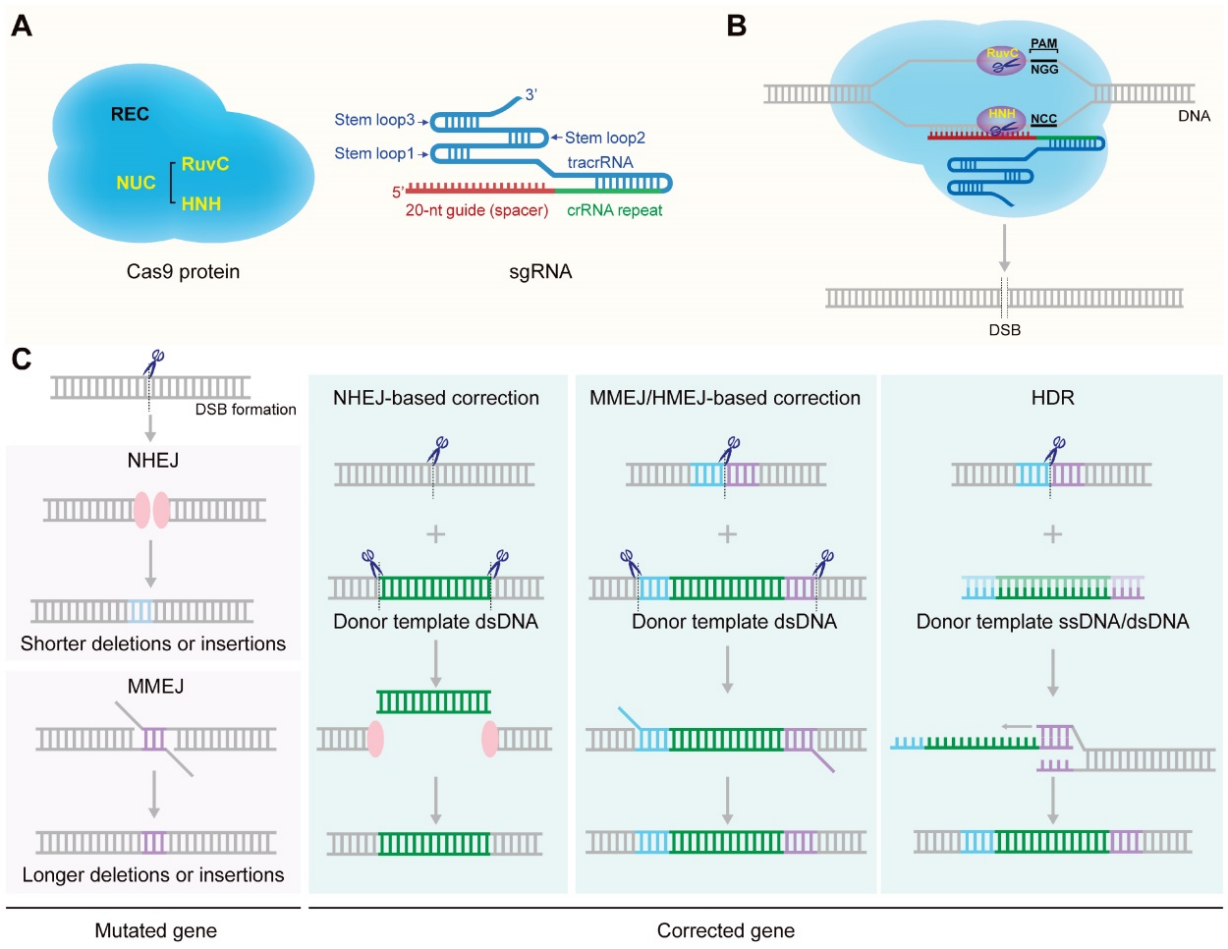
Schematic illustration of the structure and molecular mechanism of the CRISPR/Cas9 system. A. structure of Cas9 protein and sgRNA. B. Formation of DSB via CRISPR/Cas9 system. C. The repair mechanisms of DSBs.

Two critical components of the CRISPR/Cas9 system are Cas9 nuclease and sgRNA (Figure 1A) 14. The CRISPR/Cas9 gene-targeting is directed by the sgRNA formed by hybridization of a tracrRNA and a crRNA 16. The targeting crRNA is composed by a ~20-nt sequence (the protospacer) complementary to the target DNA with the sequence requirement of a protospacer adjacent motif (PAM) (5'-NGG for the mostly used SpCas9) 30. The tracrRNA hybridizes to the crRNA and binds directly to the Cas9 nuclease to form a ribonucleoprotein (RNP) complex 31. The CRISPR/Cas9 gene-editing function is directed by the Cas9 nuclease. There are two lobes of the Cas9 nuclease: a nuclease (NUC) lobe and a target recognition (REC) lobe. The RuvC, HNH and PAM-interacting domains comprise the NUC lobe. The HNH domain cleaves the target DNA strand complementary to crRNA while the RuvC domain cleaves the other strand, finally resulting in a double-stranded break (DSB) at the target site (Figure 1B) 14, 32. DSBs are mainly repaired by nonhomologous end joining (NHEJ) or homology directed repair (HDR) pathway (Figure 1C) 18, 33-35. NHEJ is error-prone and typically leads to indels (insertion and/or deletion of nucleotides) at the site of the break which would knockout a gene when the reading frame was shifted (Figure 1C) 36, 37. For the HDR pathway, a template DNA containing a sequence homologous to the DSBs is required to repair or precisely modify the genome of proliferating cells 38. Thus, utilizing the mechanism of HDR can repair disease-causing mutations or knock in customized sequences at DSBs loci to induce desired genotype (Figure 1C). NHEJ, the principal and most rapid pathway for DSB repair, is active throughout the cell cycle, while HDR is active during the S or G2 phase of cell cycle 33, 39. It is worth mentioning that the efficiency of HDR-mediated gene replacement or knock-in is much lower than gene knock-out 40, 41. NHEJ can also be applied to mediate genome integration in the presence of a donor vector that contains the desired transgene flanked by a CRISPR target site (Figure 1C) 42-44. Besides the classical NHEJ pathway, there are other alternative error-prone repair mechanisms of end joining, such as microhomology-mediated end joining (MMEJ) (Figure 1C). MMEJ repairs DSBs by annealing microhomologies, which are 2 to 20-bp stretches of overlapping bases flanking the DSB 45. MMEJ is highly mutagenic with a high frequency of missense and indels in the franking sequences 46. At the same time, MMEJ can also be utilized to mediate precise integration of exogenous DNA as another powerful complementary strategy to the HDR-based knock-in strategy 47. In the presence of a donor template harboring microhomology arms (5-40 bp) flanking the genomic target locus, MMEJ could be used to mediate precise insertion of exogenous DNA (Figure 1C) 48-51. In addition, homology-mediated end joining (HMEJ)-based strategy with long homology arms (~800 bp) was reported to achieve precise gene integration with greater knock-in efficiency than MMEJ-based strategy (Figure 1C) 52-54.

A very necessary prerequisite for CRISPR/Cas9 complex to function is the efficient delivery of the complex into nucleus of target cells. The CRISPR/Cas9 complex could be delivered in the forms of plasmid DNA (pDNA), messenger RNA (mRNA) or ribonucleoprotein (RNP, Cas9 protein complexed with sgRNA) 55. Direct delivery of RNP complex avoids many of pitfalls associated with pDNA or mRNA delivery 56-59. RNP delivery enables the swiftest genome editing by reason of eliminating the need for intracellular transcription and translation. Meanwhile, the transient genome editing not only permits high editing efficiency, but also reduces off-target effects, insertional mutagenesis, and immune responses 60, 61. What's more, RNP delivery offers a robust platform for cells with low transcription and translation activity, and also enables advances in genome-editing efficacy in multiple contexts including embryonic stem cells, induced pluripotent stem cells, and tissue stem cells 62. These advantages of RNP delivery make it a promising platform in the field of CRISPR/Cas genome editing.

The strategies and materials for intracellular delivery of proteins and nucleic acids have been well developed and reviewed 6, 63-70. However, considering the unique characteristics of RNP complexes, i.e. the complex composition and charge property, there are specific requirements when developing delivery systems for RNP when compared with proteins and nucleic acids. In this review, we systematically summarize the delivery strategies of Cas9 RNP for genome editing. Methods for RNP delivery including physical approaches such as microinjection, electroporation, biolistic and microfluidic techniques, and synthetic carriers such as lipid nanoparticles and cell-derived vesicles, polymers, nanogels, inorganic nanoparticles and DNA nanoclews were reviewed. The principles and advantages of these strategies and materials in RNP delivery are discussed. We hope to provide a comprehensive review on the rational design of materials and techniques for Cas9 RNP delivery and genome editing.

## Physical approaches for RNP delivery

### Direct penetration

#### Microinjection

**Figure 2:**
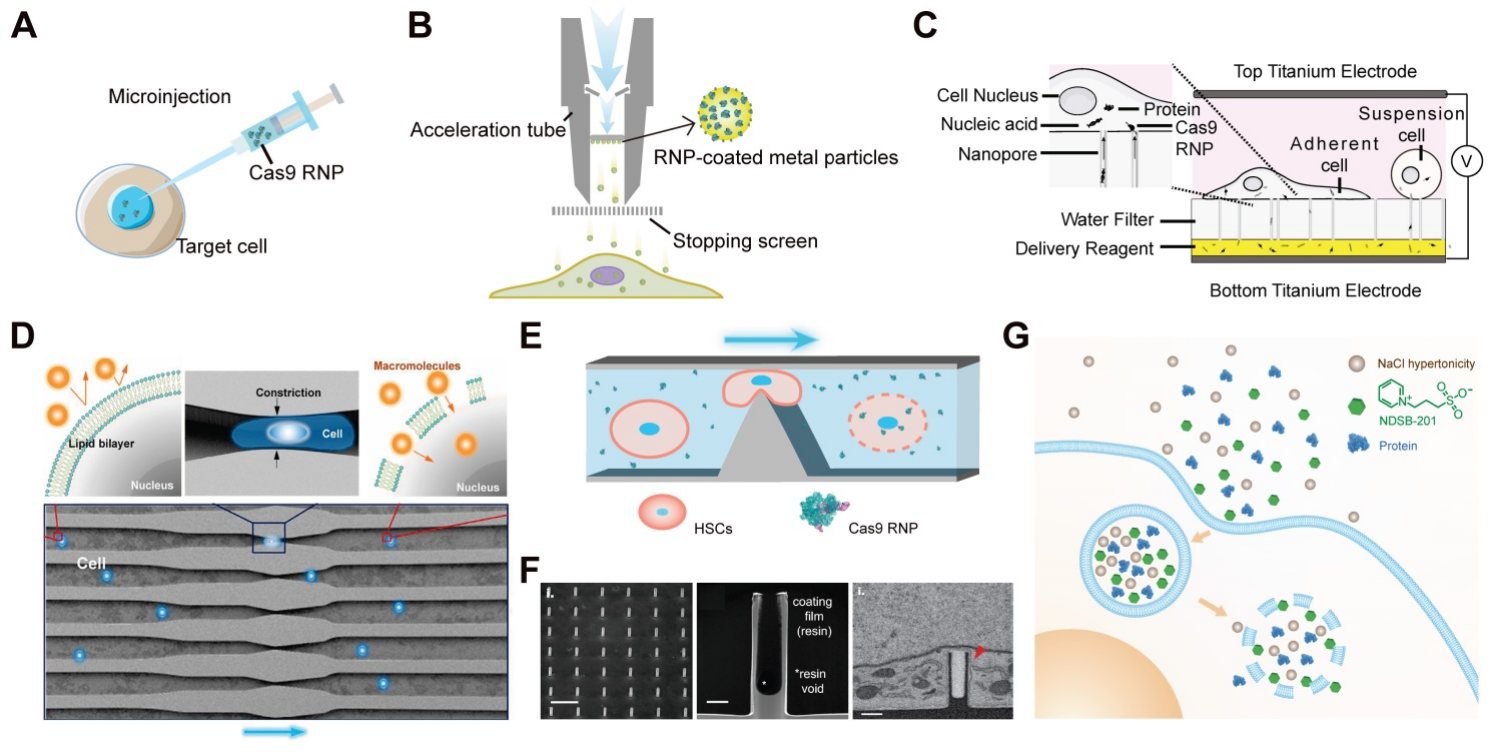
The physical approaches for Cas9 RNP delivery. **A** and **B**. Schematic diagrams of microinjection (**A**) and biolistics (**B**) for RNP delivery. **C**. Schematic of the NanoEP electroporation device. Reduced with permission from 119. Copyright 2019, National Academy of Sciences. **D**. Illustration of the original microfluidic device for macromolecules delivery via cell squeezing. Adapted with permission from 122. Copyright 2013, National Academy of Sciences. **E.** Workflow of the silicon microfluidic chip.** F.** Images showing the nanostructures of silicon nanotube. Reduced with permission from 127. Copyright 2020, Wiley-VCH. **G**. Schematic of the iTOP system.

Microinjection is a direct physical method to deliver RNP into living cells through a glass micropipette (Figure 2A). Microinjection allows quantitative control of injected Cas9/sgRNA complex and break through the limitation of molecular weight 71. Up to now, injection of RNP has been successfully implemented in embryos of various organisms, such as Zebrafish 72-74, mouse 75, rabbit 76 , axolotl 77, reef-building corals 78, spider mite 79, and olive fruit fly 80. However, embryo microinjection may cause inevitable damage to cells and therefore requires highly skilled manipulation and expensive equipment, which is difficult to implement for non-specialist laboratories. In addition, some species are recalcitrant to the embryonic microinjection for their fragile eggs, or because they are not oviparous 28. To circumvent the obstacle of embryo microinjection, researchers developed a method for delivering oocyte-targeted RNP into the arthropod germline by injection into adult female mosquitoes 81, 82, and silverleaf whitefly 83, which resulted in efficient and heritable genome editing of the offspring. A 41 aa peptide (P2C) derived from *D. melanogaster* Yolk Protein 1 (DmYP1) was fused with Cas9 for targeted delivery of RNP into the ovaries via receptor-mediated endocytosis. The injection of P2C-Cas9 RNP into the germline tissue of adult female mosquitoes led to efficient genome editing when coupled with an endosomolytic regent chloroquine.

#### Biolistics

Biolistics, short for “biological ballistics”, is another direct physical method used to deliver biomacromolecules into cells, mainly plant cells. The biomolecules were coated onto gold or tungsten microparticles, which could be accelerated to high velocity by pressurized gas, chemical explosion, high-voltage electronic discharge or helium shock 67. As a result, the bound biomolecules could be shot into target cells through cell walls and membranes (Figure 2B). By this biolistic strategy, pre-assembled RNP were delivered into maize embryo cells which demonstrated efficient gene mutation and recovery of maize with mutated alleles at high frequencies 84. Efficient genome editing was achieved in maize 84, wheat 85, 86, rice 87, and diatom 88, potato 89, cryptococcus neoformans 90, marine microalga 91 and etc. by biolistic delivery of Cas9 RNP complexes.

### Membrane disruption by electroporation

Electroporation can disturb the phospholipid bilayer of cell membranes via an electrical pulse to produce temporary nanopores on membranes through which biomacromolecules such as proteins, nucleic acids and RNPs can transport across 92. Electroporation offers a transient and stable transfection of RNP for different types of cells, such as human CD34^+^ hemopoietic stem/progenitor cells (HSPCs) 60, 93-100, human embryonic stem cells (hESCs) 101, human primary neonatal fibroblast cells 101, human induced pluripotent stem cells (iPSCs) 102, human B cells 103, human CD4^+^ T cells 104, CAR-T cells 105, human embryonic kidney (HEK) 293T cells 106, 107, mouse CD8^+^ T cell 108, mouse neural stem cells 109, mouse skin stem cells 110, mouse pronuclear-stage embryos 111, mature primary mouse innate lymphocyte cells 112, rabbit fibroblast cells 102, green alga *Chlamydomonas reinhardtii* 113, 114, *Trypanosoma cruzi* 115. However, the high voltage pulses during electroporation usually cause substantial cell death 116, 117. An alternate approach is to introduce a single-cell electroporation using a nanofountain probe system, which allows efficient transfection of precise amount of RNP with high cell ability 118. The nanofountain probe contains a silica cantilever with microchannel-embedded and a pyramidal tip with an opening of 500 nm, which enables a localized and well-controlled electric filed upon lower voltage. Another study reported a nanopore-electroporation (NanoEP) platform with high delivery efficiency and cell viability for RNP delivery 119. The NanoEP platform built on two flat titanium electrodes and a polydimethylsiloxane (PDMS) scaffold with a track-etched polycarbonate water-filter membrane embedded with nanopores 100 nm (±10 nm) in diameter. These nanopores allowed local electric field upon low-voltage and resulted in a small number of nanochannels on the cell membrane with efficient RNP delivery into both suspension and adherent cells (Figure 2C). It is worth noting that enhancing sgRNA stability by chemical modification can further improve the genome editing efficiency of electroporation-mediated RNP delivery 96, 97.

To increase RNP-mediated HDR efficiency in diverse clinically relevant primary cell types, the addition of 16 bp truncated Cas9 target sequences (tCTSs) at the end of donor DNA template homology arms enabled Cas9-mediated RNP binding to shuttle the donor DNA template to the nucleus. Before electroporation, the RNP complexes were stabilized by an anionic polymer such as polyglutamic acid, which h further increased the HDR efficiency. Combining these two strategies, the HDR efficiency by RNP electroporation was improved approximately 2- to 6-fold on various primary cells 120.

### Membrane deformation

#### Microfluidics

Microfluidics is a technique that manipulates small amounts of fluids in channels with dimensions of a micrometer or tens of micrometers. The cell membranes will experience a rapid mechanical deformation when passing through the microfluidic channels, generating transient membrane holes once the compressive and shear forces exceed the phospholipid bilayer stress limitation, though which biomolecules can enter into the cytoplasm via passive diffusion (Figure 2D) 121-123. Microfluidics-based delivery has the advantage of high-throughput delivery of almost all macromolecules into a wide variety of cells. The microfluidic strategy was used to deliver Cas9 RNP into cells for genome editing 124. The microfluidic chip consists of 10 arrays of micro-constrictions though which curved tunnels were formed. Cas9 RNP complexes targeting p38 mitogen-activated protein kinases (MAPKs) were efficiently delivered into human MDA-MB-231 and SUM-159 breast cancer cells and primary human CD4^+^ T cells, resulting in indels frequencies of 43%, 47% and 33%, respectively. Cas9 RNP delivery induced lower off-target mutations frequency (0.8%) than plasmid transfection (4.7%) with comparable on-target mutations frequency. The microfluidic chip also induced HDR-mediated knock-in efficiency of 7.8% in human primary T cells. Similarly, microfluidic strategy mediated RNP delivery achieved high genome editing efficiency in human hematopoietic stem cells (HSCs) (Figure 2E) 121. The silicon microfluidic chip was fabricated by photolithography and reactive ion etch (RIE) technologies with several parallel nano-silicon-blade structures. The repeats of nano-silicon-blade structure, cell concentration and fluid rate were optimized for HSCs. The microfluidic chip mediated RNP delivery resulted in the decrease of EGFP expression by ~80% and efficiently disrupted the p42 isoform in *C/EBPα*. In addition, the HSCs delivered by the microfluidic chip kept inherent pluripotency for longer time than those by electroporation.

#### Filtroporation

Filtroporation is a technique that forces cell suspensions through uniformly sized micropores in a filter membrane to generate mechanical deformation and transient membrane holes just like microfluidics 67, 125. Filtroporation was demonstrated to be applicable for RNP-mediated genome editing in HSCs 126. The filtroporation device consists of a silicone washer, a stainless-steel mesh, a hydrophilic track-etched polycarbonate filter membrane and a polytetrafluoroethylene (PTFE) washer. The filter holder is connected to a syringe, which serves as a reservoir of the RNP and HSC mixture solution. The mixture can be pushed through the filter membrane by nitrogen pressure and collected into a tissue culture plate. After treatment, the expression of β2-microglobulin (β2M) was reduced by 63.1% with a cell recovery of 63.7%. The filtroporation system also induced 44% indels on the γ-globin (HBG) gene in HSCs. In addition, filtroporation did not impair multilineage potential and engraftment of HSCs in sub-lethally irradiated non-obese (NOD)/severe combined immunodeficiency (SCID)/*Il2rg*^-/-^ (NSG) mice.

#### Nanotube

A mechanotransfection platform comprising vertically aligned silicon nanotube (VA-SiNT) arrays was reported for intracellular RNP delivery (Figure 2F) 127. It is a new type of nanowire-mediated delivery system that doesn't need further surface functionalization, local electric field, or complicated microfluidic integration. The programmable SiNT arrays were fabricated via a combination of direct e-beam lithography (EBL) and deep reactive ion etching (DRIE), which offered high levels of reproducibility and flexibility. The hollow structure inside SiNTs allowed effective loading of various biomolecule cargoes, and these cargoes could be delivered into GPE86 mouse embryonic fibroblast cells (MEFs) via deformation-related active endocytosis pathway and/or passive diffusion with minimal impact on cell viability. SiNT arrays successfully induced intracellular RNP delivery and genome editing in the cells.

### Induced transduction by osmocytosis and propanebetaine (iTOP)

iTOP is a transduction method mediated by a combination of NaCl-mediated hypertonicity and a propanebetaine 62. For the iTOP delivery system, NaCl-mediated hypertonicity induced efficient internalization of RNP via macropinocytosis. Osmoprotectants such as glycerol and glycine were added to rescue the hypertonicity-induced cytotoxicity, and the endosomolytic reagent propanebetaine is responsible for releasing the internalized RNP complexes from endolysosomes (Figure 2G). After two-round iTOP transduction, Cas9 RNP was effectively delivered into KBM7 cells and hESCs with genome editing efficiencies of 56.1% and 26.3%, respectively. This method allows proteins for cell manipulation in a non-integrated manner, suited for binary systems, where individual transient cell manipulation results in permanent changes in cell function identity or epigenetic state.

### Protoplast transformation

Polyethylene glycol (PEG)-mediated transformation is a common and efficient strategy for genome editing of plant or fungi cells 128. PEG can cause the protoplasts to clump together and induce interaction consequent between DNA and cell surface 129, 130. It's a method that suitable for delivering various molecules into protoplast cells without the need of any carrier. The critical process of PEG-mediated transformation is the isolation and culture of plant protoplasts due to the existence of cell walls on plant cells 131. Biomacromolecules can be delivered into the plant protoplast in the presence of PEG. A large number of works have been performed to optimize PEG-mediated RNP delivery into various plant and fungi protoplast cells 85, 132-141. In allusion to the plant species whose protoplasts are difficult to be isolated and cultured, researchers bypassed the problem by direct delivery of RNP into plant zygotes which were produced by *in vitro* fertilization of isolated gametes 142.

## Materials for RNP delivery

### Virus-like particles

**Figure 3:**
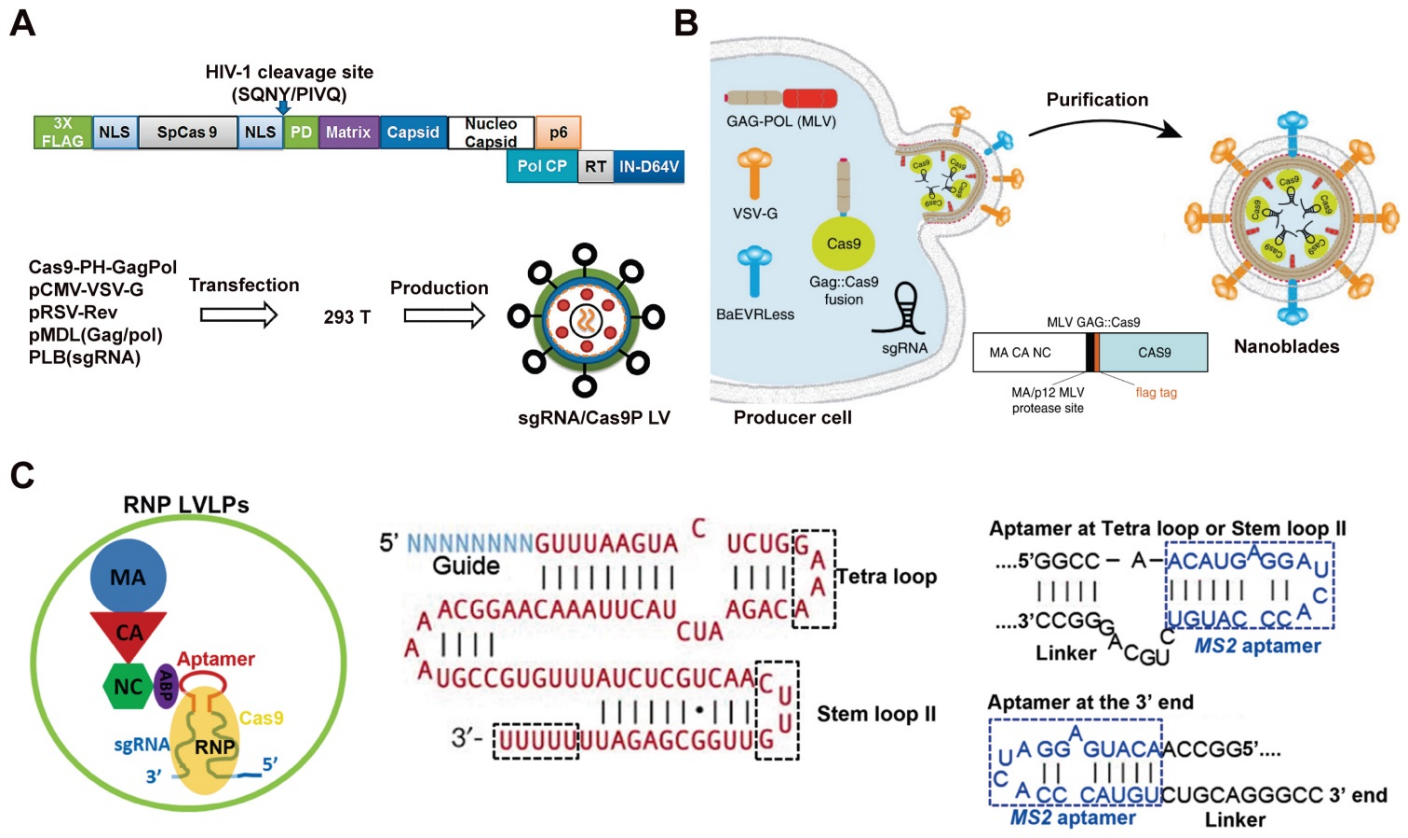
Virus-like particles for Cas9 RNP delivery. **A**. Schematic of 'all in one virus' production. Adapted with permission from 143. Copyright 2016, Springer Nature. Creative Commons CC BY. **B**. Scheme describing the production of MLV-like particles. Reduced with permission form 144. Copyright 2019, Springer Nature. Creative Commons CC BY. **C**. Illustration of a lentivirus-like RNP delivery system. Adapted with permission from 145. Copyright 2019, Oxford University Press. Creative Commons CC BY.

The direct use of virus vectors to deliver Cas9 plasmid may lead to off-target effects and unexpected immunoreaction. To resolve this problem, lentivirus (LV) vectors were pre-packed with Cas9 protein for safer genome editing 143. Cas9 protein was incorporated into lentiviral particles by fusing FLAG-tagged Cas9 sequence to the N terminus of Gag (Cas9-PH-GagPol). The human immunodeficiency virus-1 (HIV-1) protease cleave site between Cas9 and Gag domain was introduced to enable the release of Cas9 protein during particle maturation (Figure 3A). To build an “all-in-one virus” vector (sgRNA/Cas9P LV), a designed sgRNA was cloned into the pLB lentiviral vector (pLB/sgRNA) under the U6 promoter, then 293T cells were co-transfected with Cas9-PH-GagPol, pLB/sgRNA, helper pMDL (Gag/Pol), pRSV-Rev and pCMV-VSV-G plasmids to produce lentiviral particles containing sgRNA-expressing vectors (Figure 3A). As a result, no detectable off-target effect was achieved by this technique whereas a 2.1% off-target cleavage was observed by Cas9 plasmid encoding LV. The gene knock-out efficacy of the virus-like particles was confirmed by targeting *CD4* gene in TZM-bl cells (16% indels) and HIV LTR (long terminal repeat) to disrupt HIV provirus in J-Lat cells (28% indels).

Some cells such as HSCs are difficult to transduce with vesicular stomatitis virus glycoprotein (VSV-G) pseudotyped LVs due to the lack of LDL receptors. Researchers have developed murine leukemia virus-like particles with both baboon retroviral envelope glycoprotein (BaEV) and VSV-G envelopes (Figure 3B). The Cas9 protein was fused on the C-terminal end of the murine leukemia virus (MLV) Gag protein with a proteolytic site, which can be cleaved by the MLV protease to release the Flag-tagged Cas9. The virus-derived particles were produced by transfecting HEK 293T cells with plasmids coding Gag::Cas9, Gag-Pro-Pol, a sgRNA, and viral envelopes (Figure 3B) 144. In the presence of polybrene, the donor DNA complex with the virus-derived particles to form an “all-in-one” vector to mediate HDR in target cells. These MLV-like particles induced efficient genome editing in various cell lines including iPSCs, mouse bone-marrow cells, and HSCs.

Besides packaging the Cas9 protein into virus-like particles by fusion expression, another research reported a lentivirus-like system that allows efficient packaging of RNP by utilizing the specific interaction between aptamer and aptamer-binding protein (ABP) 145. Aptamer are short stretches of nucleotides with a specific three-dimensional structure, which can be selected *in vitro* by artificial combination method or systemic evolution of ligands by exponential enrichment (SELEX), and the specific aptamer can bind ABP with high affinity and specificity. It was found that replacing the tetraloop of sgRNA scaffold with a *com* aptamer could preserve the function of sgRNA, and therefore the Cas9/*com*-sgRNA RNP were efficiently packed into LV-like particles through the specific interaction of *com* aptamer with ABP, which was incorporated into LV-like particles by fusing with the lentiviral nucleocapsid protein (Figure 3C).

### Lipid nanoparticles

#### Cell-derived extracellular vesicles (EVs)

**Figure 4:**
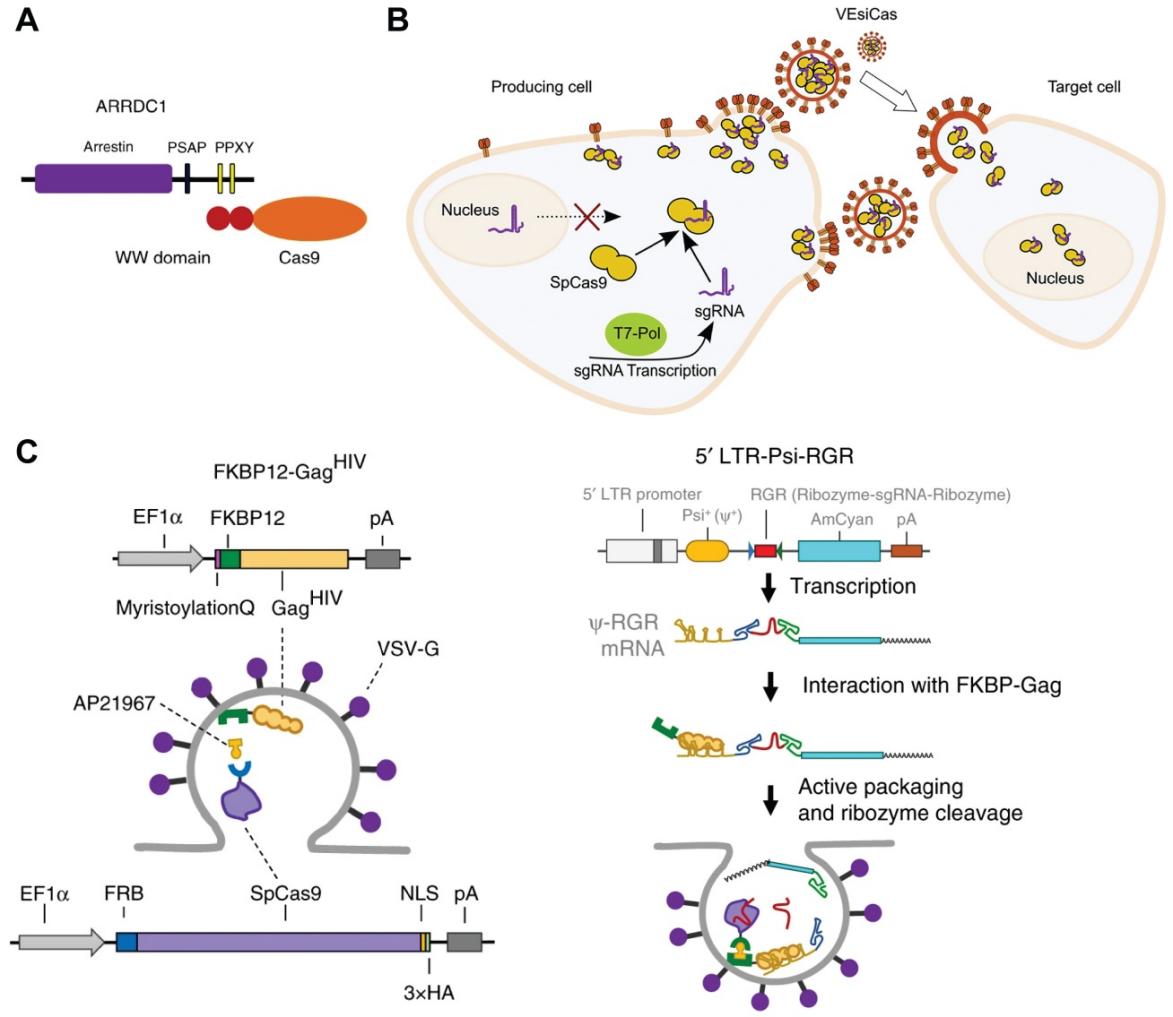
Cell-derived extracellular vesicles for Cas9 RNP delivery. **A**. Packing strategy of recruiting Cas9 into ARMMs via specific interaction between WW domain and PPXY motifs of ARRDC1. Reprinted with permission from 149. Copyright 2018, Springer Nature. Creative Commons CC BY. **B**. Schematic of the production of RNP-packaging fusogenic VSV-G vesicles. Reprinted with permission from 153. Copyright 2018, Elsevier. Creative Commons CC BY-NC-ND. **C**. Selective packaging of Cas9 and sgRNA into extracellular nanovesicles. Adapted with permission from 155. Copyright 2020, Copyright Springer Nature. Creative Commons CC BY.

Cells exchange information through several mechanisms such as secretion of growth factors and chemokines. EVs secreted by most eukaryotic cells are described as important vehicles for intercellular communication 146. According to the biogenesis, EVs can be classified into three main classes: exosomes, microvesicles and apoptotic bodies 147. These EVs possess the inherent ability of delivering functional proteins, nucleic acids and RNP into different cells 148. For example, arrestin domain containing protein 1 [ARRDC1]-mediated microvesicles (ARMMs) have been developed for Cas9-sgRNA RNP delivery 149. ARRDC1 located on plasma membrane can recruit TSG101 from endosomes to the membrane and mediate the release of ARMMs 150. Proteins could be packaged into ARMMs by direct fusion to ARRDC1 or fusion to WW domains which could specifically interact with ARRDC1 (Figure 4A). 2WW-Cas9-sgRNA or 4WW-Cas9-sgRNA was conducted into a px330 vector and co-transfected with ARRDC1-expression pcDNA3.1 vector into cells. Then, 2WW-Cas9 or 4WW-Cas9 and sgRNA were incorporated into ARMMs via molecular recognition between WW and ARRDC1. The ARMMs successfully delivered Cas9 RNP into recipient U2OS cells and induced genome editing 149. CD63 is a member of tetraspanin family and expressed on the inner surface of exosome membrane. By fusing CD63 protein with GFP and Cas9 protein with a GFP-binding nanobody, respectively, Cas9 protein and RNP could be encapsulated into exosomes specifically 151. Overexpression of the spike VSV-G in human cells promotes the release of fusogenic vesicles, which incorporate proteins in the plasma of producer cells and deliver them into recipient cells in virtue of the binding and fusion properties 152. Expressions of Cas9 protein and sgRNA together with VSV-G in HEK 293T cells could produce fusogenic VSV-G vesicles (VEsiCas) (Figure 4B). VEsiCas achieved ~60% and ~30% indels on *CXCR4* and *VEGFA* in HEK293T cells, respectively. In addition, multiplexed VEsiCas targeting genomic deletions induced ~17% efficiency in the *EGFP* locus of HEK293-EGFP reporter cells 153. Further co-expression with CherryPicker Red resulted in fluorescence labeling of VSV-G vesicles 154.

Rapamycin can simultaneously bind to the 12-kDa FK506-binding protein (FKBP12) and the FKBP-rapamycin binding domain (FRB). Cas9 protein can be selectively packaged into budding EVs using the dimerization of FKBP12 and FRB. FKBP12 fused membrane-anchoring protein Gag and FRB-fused Cas9 could form a dimer in the presence of AP21967, a rapamycin analog (Figure 4C). To further incorporate sgRNA into the EVs, an expression vector containing a Tat activation response element (TAR) in the 5′ LTR promoter region and an extended Psi (Ψ^+^) packaging signal with specifically-binding ability to nucleocapsid of Gag was constructed. Hence, sgRNA could be selectively and actively packaged into the EVs loaded with Cas9 protein. The prepared EVs mediated efficient genome editing in iPSCs, iPSC-derived cortical neurons, myoblast cells and induced sustained genomic exon skipping in mouse models (Figure 4C) 155.

#### Synthetic lipid nanoparticles

**Figure 5:**
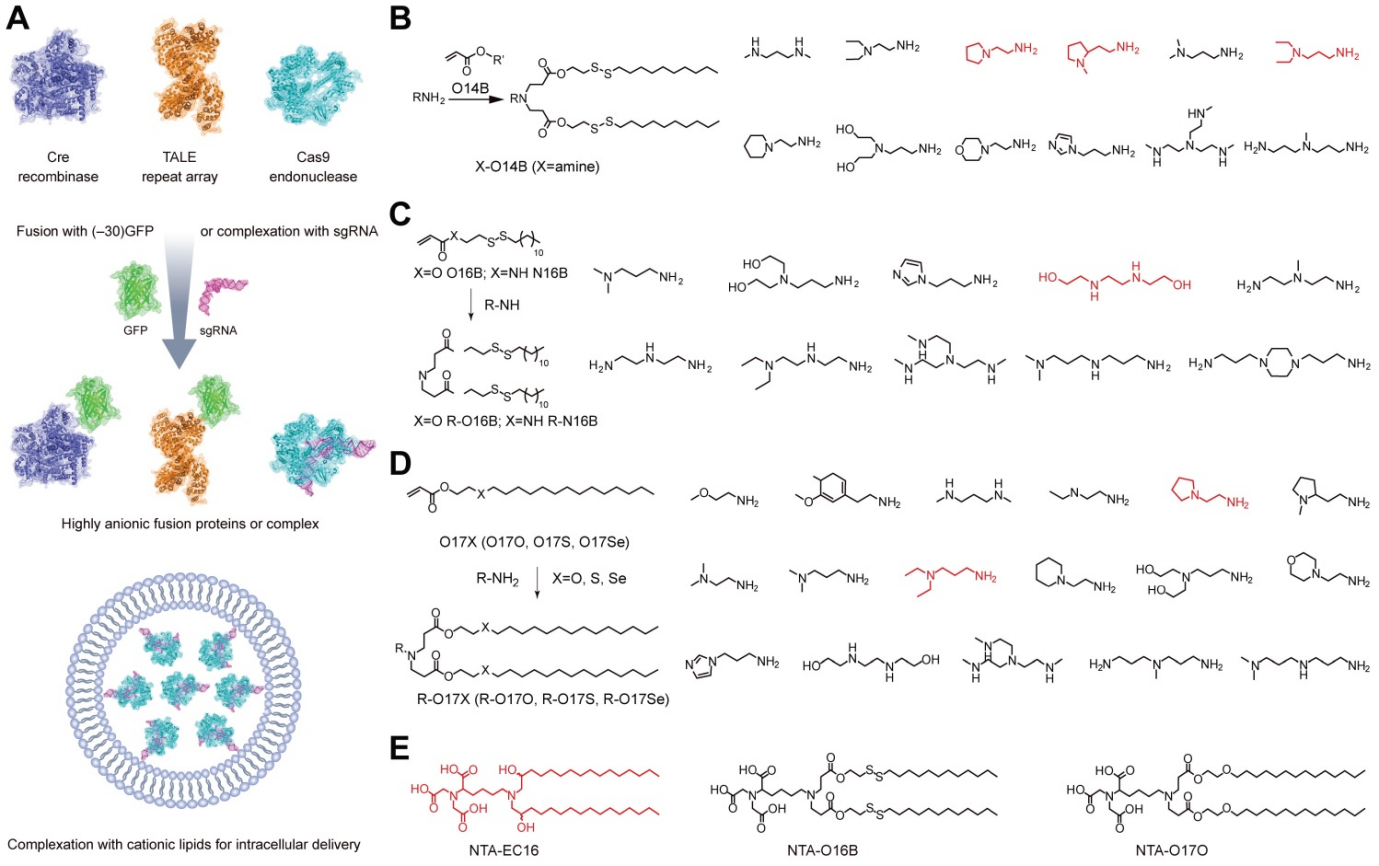
Intracellular delivery of Cas9 RNP by lipids. **A**. Cationic lipid-mediated delivery of CRISPR system by RNP complex or fusing Cas9 protein with anionic GFP. **B**. Bioreducible cationic lipid library for the delivery of genome editing systems 170. **C**. Expansion of bioreducible cationic lipid library for Cas9 RNP delivery 171.** D**. Synthesis of cationic chalcogen-containing lipids for Cas9 RNP delivery 172. **E**. Non-cationic NTA-containing lipidoids for Cas9 RNP delivery. Red color identifying the leading amine heads or lipidoid for the intracellular delivery of Cas9 RNP 173.

Cationic lipid nanoparticles are the most commonly used materials for transferring exogenous genetic materials into cells 6. Cationic lipids consist of three structural domains: a cationic headgroup, a hydrophobic portion, and a linker between the two domains. The uptake mechanism of cationic lipid-nucleic acid complexes (lipoplexes) has been systematically reviewed 156. Early work indicated that the intracellular delivery of lipoplexes was mediated by direct membrane fusion 157, but it is now agreed upon that the internalization occurs mainly through endocytosis 158, 159. After internalization, lipoplexes disrupt the endosomal membrane, resulting in a flip-flop reorganization of phospholipids. These phospholipids then diffuse into the lipoplexes and interact with the cationic lipids which leads to the release of nucleic acids into the cytoplasm 6. Distinct from nucleic acids with high density of negative charges, Cas9 protein is highly cationic (theoretical net charge: +22) and thus cannot directly complex with cationic lipids via electrostatic interaction. However, the Cas9/sgRNA RNP is negatively charged and could be delivered into mammalian cells by using commercial cationic lipids such as Lipofectamine RNAi^MAX^, Lipofectamine 2000 and Lipofectamine 3000 etc. In addition, the cationic Cas9 could be fused with a negatively charged GFP (-30) to increase its binding to cationic materials (Figure 5A) 56, 160. Delivery of Cas9/sgRNA RNP by cationic lipids allowed genome editing in serum containing medium and induced up to 80% gene disruption efficiency. The high genome editing efficiencies of cationic lipid mediated RNP delivery were confirmed at different targets on various mammalian cells 160 and plant protoplast cells 161. SaCas9 is a Cas9 nuclease form *Staphylococcus aureus* that recognizes a longer PAM 5′‐NNGRRT‐3′. The SaCas9/sgRNA RNP could be also delivered by cationic lipids with high efficiency 162. The commercial lipids such as Lipofectamine 2000 also allowed *in vivo* delivery of RNP to edit a pathogenesis-related gene for the treatment of nongenetic degenerative diseases 163. These lipid materials were also applied for the co-delivery of RNP and template DNA for HDR 56, 164, 165.

Based on the remarkable efficiency of cationic lipids in RNP delivery, Thermo-Fisher developed a new transfection reagent termed CRISPR^MAX^ for RNP delivery. Upon optimization of transfection conditions, the genome editing efficiencies achieved 55%, 75% and 85% in human iPSCs, mouse ES cells and HEK293FT cells, respectively 166. Researchers utilized CRISPR^MAX^ to deliver RNP and templated DNA into p53^+/+^ and p53^-/-^ human retinal pigment epithelial cells to investigate the relationship between p53-mediated DNA damage response and Cas9-mediated genome editing 167. They found that Cas9-induced DSBs lead to a transient, p53-dependent cell cycle arrest at G1 through p53-p21-pRB axis independent of the locus targeted, and inhibition of p53 can improve the HDR efficiency. Furthermore, CRISPR^MAX^ efficiently delivered RNP targeting p53 into dog oviductal epithelia cells cultured in a dynamic microfluidic chip, and successfully created an *in vitro* model that recapitulated human tubal intraepithelial carcinoma (STIC) 168. To expand the applications of RNP-mediated genome editing, researchers developed a scaffold-mediated delivery platform for CRISPR/Cas9 genome editing 169. Complexes of RNP and CRISPR^MAX^ were adhered onto the electrospun fiber scaffolds which were coated with polyDOPA-melanin and laminin. U2OS cells took up these complexes directly from the scaffold via reverse transfection. As expected, effective genome editing was detected in the cultured cells.

**Figure 6:**
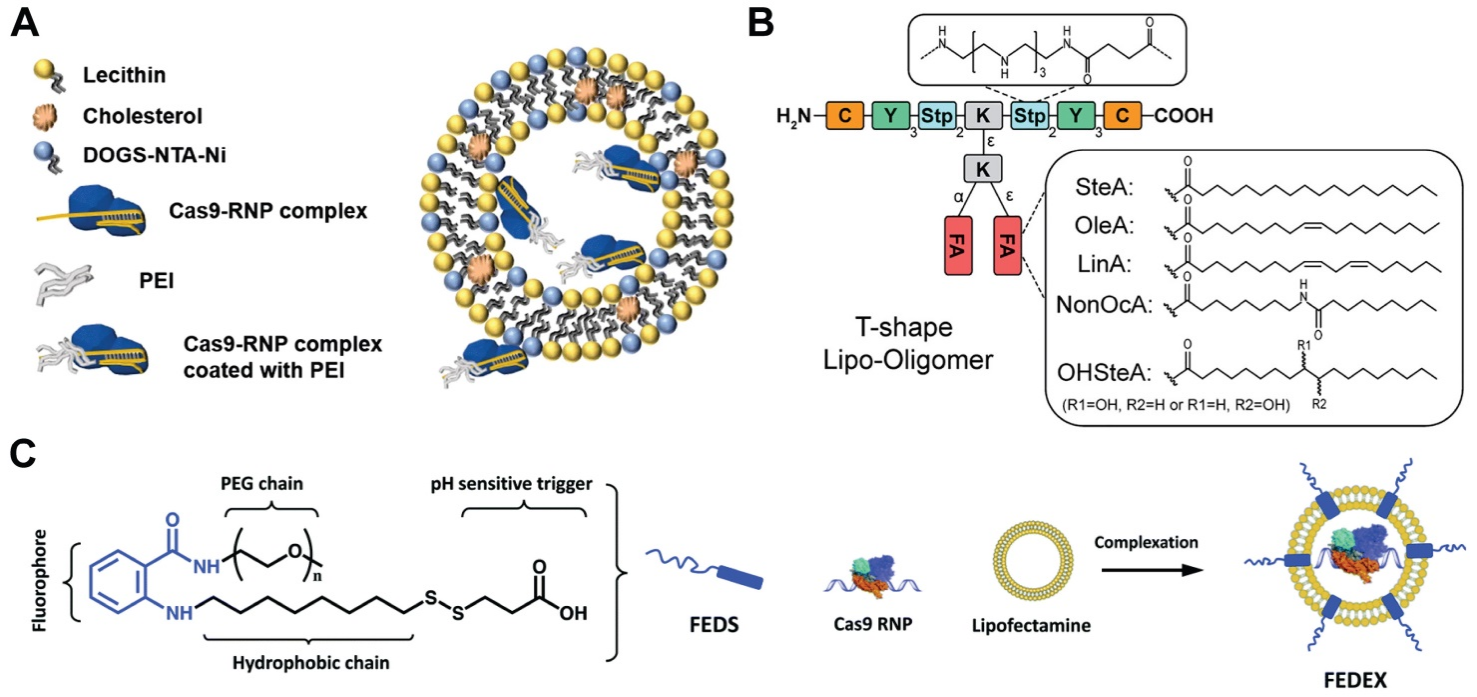
Lipid vehicles for Cas9 RNP delivery. **A**. Lecithin-based liposomal delivery system for Cas9 RNP delivery. Reduced with permission from 174. Copyright 2019, Springer Nature. Creative Commons CC BY. **B**. Illustration of T-shape lipo-OAAs with different fatty acids, in which lipo-OAA-containing OHSteA was superior to others in higher genome editing efficiency. Reduced with permission form 175. Copyright 2020, American Chemical Society. **C**. A fluorescent surfactant used to enhance the Cas9 RNP delivery of lipofectamine. Adapted with permission from 176. Copyright 2019, Royal Society of Chemistry.

Besides commercial lipid reagents, Xu et al. used a combinatorial library strategy to discover novel and efficient lipid materials for intracellular RNP delivery. They synthesized 12 bioreducible lipids by Michael addition reactions between compounds bearing primary or secondary amines and an acrylate containing a disulfide bond and a 14-carbon hydrophobic tail (Figure 5B) 170. All lipidoids were formulated with 1,2-dioleoyl-*sn*-glycero-3-phosphoethanolamine (DOPE), C16-PEG_2000_-ceramide and cholesterol to stabilize the lipid nanoparticles. These bioreducible lipidoids were used to deliver Cas9/sgRNA RNP targeting genomic EGFP reporter gene. The lead material discovered in the library showed 70% genome editing efficiency. The further expand the bioreducible cationic lipidoid library by introducing an amide linker between the hydrophilic amine heads and aliphatic tail groups for Cas9 RNP delivery (Figure 5C) 171. In a separate study, a library of cationic chalcogen-containing lipids were designed as candidates to deliver Cas9 RNP 172. The chalcogen-containing lipids were synthesized by the reaction of lipophilic tails containing O, S and Se ethers (O17O, O17S and O17Se) with amine bearing compounds (Figure 5D). The results indicate that lipids with O17Se tails are more likely to form efficacious lipidoid nanoparticles (LNPs) for Cas9 RNP delivery. Besides cationic lipids, a library of noncationic ones were designed to deliver His-tagged proteins (Figure 5E) 173. The noncationic lipids were synthesized by conjugating a nitrilotriacetic acid (NTA) group onto a hydrophobic tail. The addition of nickel ions could mediate the binding of His-tagged proteins onto NTA-conjugated lipids. These lipidoids demonstrated high efficiency in the delivery of His-tagged Cas9 RNP into mammalian cells. Similarly, a lipid nanoparticle consisting of lecithin, cholesterol and 1,2-dioleoyl-*sn*-glycerol-3-[(N-(5-amino-1-carboxylpentyl)iminodiacetic acid)succinyl] (nickel) (DOGS-NTA-Ni) was developed for RNP delivery 174. DOGS-NTA-Ni was used to load His-tagged Cas9 protein by the lipid nanoparticles, and the prepared nanoformulations were further coated with a cationic polymer polyethyleneimine (PEI) to increase the Cas9 RNP loading efficiency (Figure 6A). The liposomal nanoparticle induced a prominent reduction in mRNA (67%) and protein (87%) expression of *DPP‑4* in SNU398 cells *in vitro* and efficiently disrupted the expression of *DPP‑4* gene in diabetic mice with a comparable therapeutic efficacy to sitagliptin, a clinically used antidiabetic drug.

A library of sequence-defined oligo(ethylenamino) amides (OAAs) containing structural motifs were reported for Cas9 RNP delivery 175. Among the designed OAAs, lipid-containing OAAs (lipo-OAAs) possess superior efficiency in Cas9 RNP delivery. Interestingly, a single hydroxy group on the lipid dramatically affected the performance of lipo-OAAs in Cas9 RNP delivery. Lipo-OAAs bearing hydroxy-stearic acid (OHSteA) showed much higher efficiencies than analogue materials without hydroxylation (Figure 6B). OHSteA formed smaller nanoparticles with Cas9 RNP (168 nm) than other lipid materials in the library (247-293 nm), and the hydroxylation of the fatty acid exhibited higher membrane lytic potential. OHSteA achieved GFP genome editing efficiencies up to 40% and 89% on Neuro2a eGFP-Luc and HeLa GFP-Tub cells, respectively. Besides development of new lipids, a fluorescent lipid FEDS was developed as a helper lipid to increase the RNP delivery efficiency of Lipofectamine 2000 176. FEDS has a membrane disruptive amphiphilic structure similar to Triton X, and the hydrophobic alkyl group on FEDS is terminated with a carboxyl group which allowed FEDS to conceal its membrane disruptive ability at pH7.4 and disrupt the endosomal membrane at an acidic pH (Figure 6C). The fluorescent property of FEDS could be used to monitor the intracellular trafficking of lipid/RNP nanoparticles.

### Cell penetrating peptides (CPPs)

**Figure 7:**
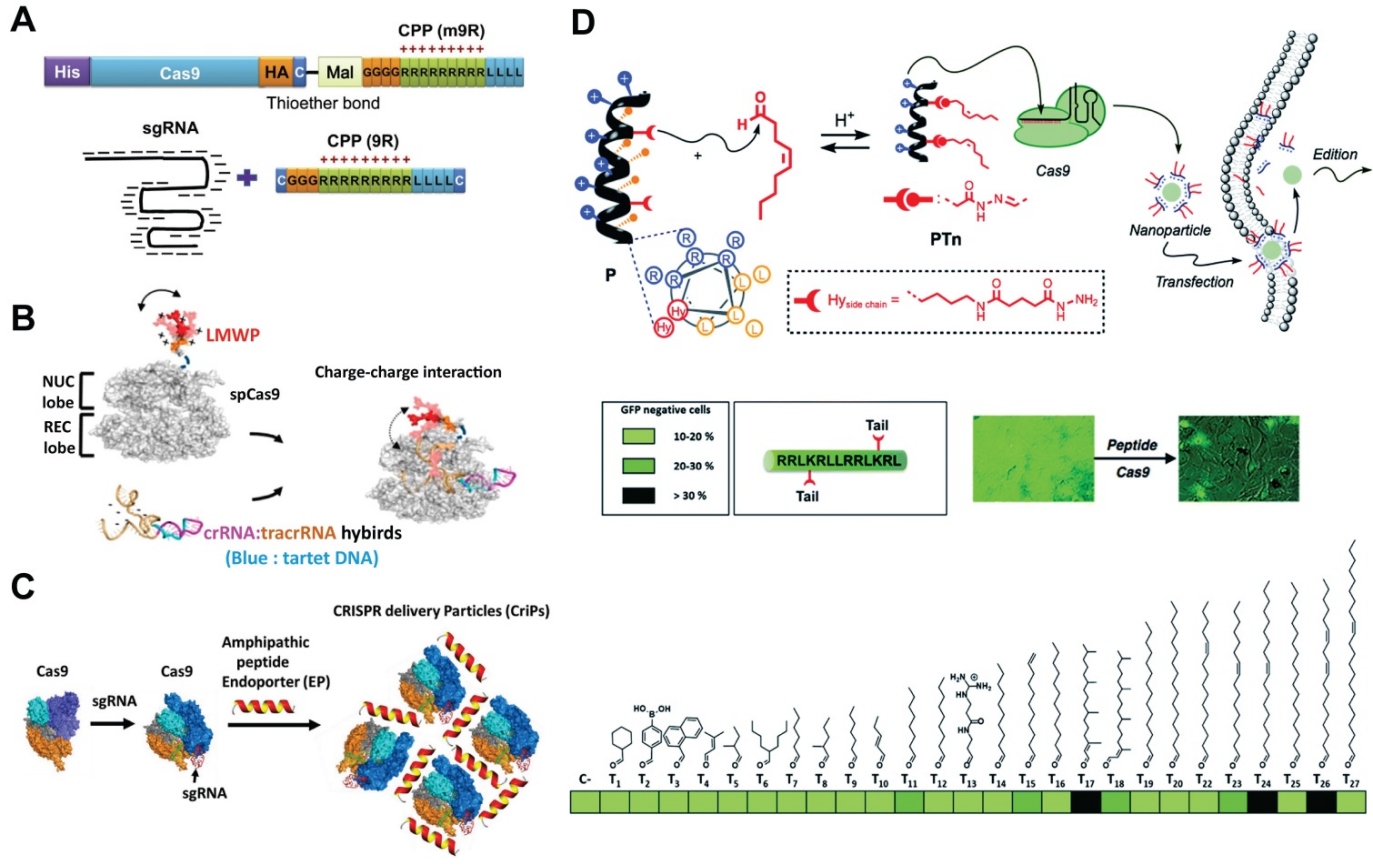
CPP- and lipopeptide-based delivery systems. **A**. CPP-conjugated Cas9 protein and CPP complexed sgRNA for intracellular delivery. Reduced with permission from 177. Copyright 2014, Cold Spring Harbor Laboratory Press. Creative Commons CC BY. **B**. Schematic of chimeric Cas9-LWMP complexed with dual RNAs. Reduced with permission from 180. Copyright 2018, American Chemical Society. **C**. Amphipathic α-helical peptides for the intracellular delivery of Cas9 RNP without covalent conjugation. Reduced with permission from 183. Copyright 2018, American Society for Biochemistry and Molecular Biology. Creative Commons CC BY. **D**. Illustration of the lipopeptide formed via a supramolecular strategy for the screening of Cas9 RNP delivery. Adapted with permission from 188. Copyright 2017, Royal Society of Chemistry. Creative Commons CC BY-NC.

CPPs enable the delivery of cargo proteins or nucleic acids into the cytosol by passive or active endocytic pathways. These peptides can be either covalently conjugated to Cas9 protein or complexed with RNP via ionic interactions for genome editing. In a pioneer study, CPP was covalently conjugated to Cas9 protein, and further complexed with CPP/sgRNA to yield RNP nanoparticles (Figure 7A). Treatment of cells with the prepared nanoparticles led to efficient gene disruptions with lower off-target effects than pDNA transfection 177. Similarly, a supercharged peptide (SCP) with the ability to directly bind to the nuclear import protein importin β1 and get access to the nucleus was screened out from a library of 12-aa peptides containing randomized sequences. The discovered SCP could effectively internalize into cells, escape form the endosomes and translocate into the nucleus. Cas9 protein fused with the SCP was then complexed with sgRNA targeting *CCR5* gene to prepare the RNP, which resulted in 15.2% editing efficiency in HeLa cells 178. Further incorporation of a dithiocyclopeptide linker containing matrix metalloproteinase 2 (MMP-2) sensitive sequence and an intramolecular disulfide bond between Cas9 and SCP could increase its editing efficiency in tumor cells 179. In a separate study, Cas9 protein was fused with a nuclear location sequence (NLS) and a low-molecular-weight protamine (LMWP) on the C-terminus (Figure 7B) 180. LMWP is a nature-sourced cell-penetrating peptide that has been widely used for gene delivery 181, while the NLS can promote the nuclear localization of Cas9 protein. The ternary complex of Cas9 fusion protein, crRNA and tracrRNA induced up to 43.9% indels in *KRAS* gene in A549 cells *in vitro* and also showed extensive synergistic anti-KRAS therapy *in vivo* 180. What's more, these Cas9-NLS-LMWP RNP system enabled simultaneous disruption of two programed cell death 1 ligands on suspension cancer cells (PD-L1 and PD-L2), leading to significantly enhanced cytotoxicity on CD8^+^ T cells 182. The Cas9 fusion protein containing arrays of Simian vacuolating virus 40 nuclear localization sequences (SV40-NLS) on the N terminus was also proved to enable Cas9 RNP-mediated genome editing in neural progenitor cells *in vitro* and neurons in distinct brain regions *in vivo* 5.

CPPs can also induce efficient CRISPR RNP delivery via non-covalent interactions. For example, an amphipathic α-helical peptide composed of leucine and histidine residues was designed for RNP delivery 183. The cationic peptide could assemble with RNP via ionic interactions and facilitate the endosomal escape of bound RNP (Figure 7C). Efficient genome editing by using this peptide was achieved in GFP-J774A.1 cells (40.4% indels), primary peritoneal exudate cells (32.8% indels) and primary pre-adipocytes (14.4% indels). Similarly, a cationic helical amphiphilic peptide for the direct cytosolic delivery of spCas9 or AsCas12a RNP was reported 184. The peptide consists of a 6× histidine-rich domain, an endosomal leakage domain and a CPP domain. The endosomal leakage domain is a cationic amphiphilic α-helical endosomolytic peptide ELD CM18 that bind and destabilize the endosomal membranes. The CPP domain is a HIV-TAT variant PTD4. This 6His-CM18-PTD4 peptide enabled robust genome editing with a less than two-minute co-incubation with spCas9 or AsCas12a RNP.

### Lipopeptides

Peptides can be decorated with lipid moieties to yield a class of lipopeptides with self-assembly behaviors and increased membrane permeability 185-187. For example, a helical amphiphilic peptide consisting of arginine, leucine and two reactive hydrazide moieties was used as the scaffold to design lipopeptides 188. A library of lipids bearing an aldehyde group was mixed with the scaffold peptide to fabricate lipopeptides via the formation of hydrazone bond. Among the candidates in the library, an oleic aldehyde based lipopeptide PT_24_ showed the highest efficiency in Cas9 RNP delivery (Figure 7D). The PT24/Cas9 RNP complexes were prepared by simply mixing the lipopeptides and Cas9 RNP together without the requirement of protein engineering or covalent fusion, and could be efficiently delivered into cells via a micropinocytosis mechanism. PT_24_ showed comparable efficiency with Lipofectamine 2000 in editing the *HPRT1* gene in several cell lines. Similarly, a blood-brain barrier permeable peptide dNP2 was conjugated with three different saturated fatty acids including caprylic acid (C8), decanoic acid (C10) and myristic acid (C14) to yield lipopeptides for Cas9 RNP delivery 189. HypaCas9 is a hyper-accurate SpCas9 with improved targeting accuracy produced by targeted mutagenesis within the REC3 domain 190. The caprylic acid-modified peptide C8dNP2 exhibited the highest ability to form homologous nanosomes, and efficiently delivered HypaCas9 RNP into HEK and GBM cells with efficiencies higher than Lipofectamine 2000 and CRISPR^MAX^ 189. In another study, modification of lipopeptides with targeting peptides enabled cell-selective gene editing 191.

### Polymers

Polymers possess the advantages of facile synthesis, flexible structures and components, ease of functionalization, and degradability, and hence have been extensively used for gene and protein delivery 65, 192-200. Up to now, polymers such as dendrimers, poly(β-amino ester)s (PBAEs), polylysine (PLL) and chitosan nanoparticles have been developed for intracellular RNP delivery.

#### Dendrimers

**Figure 8:**
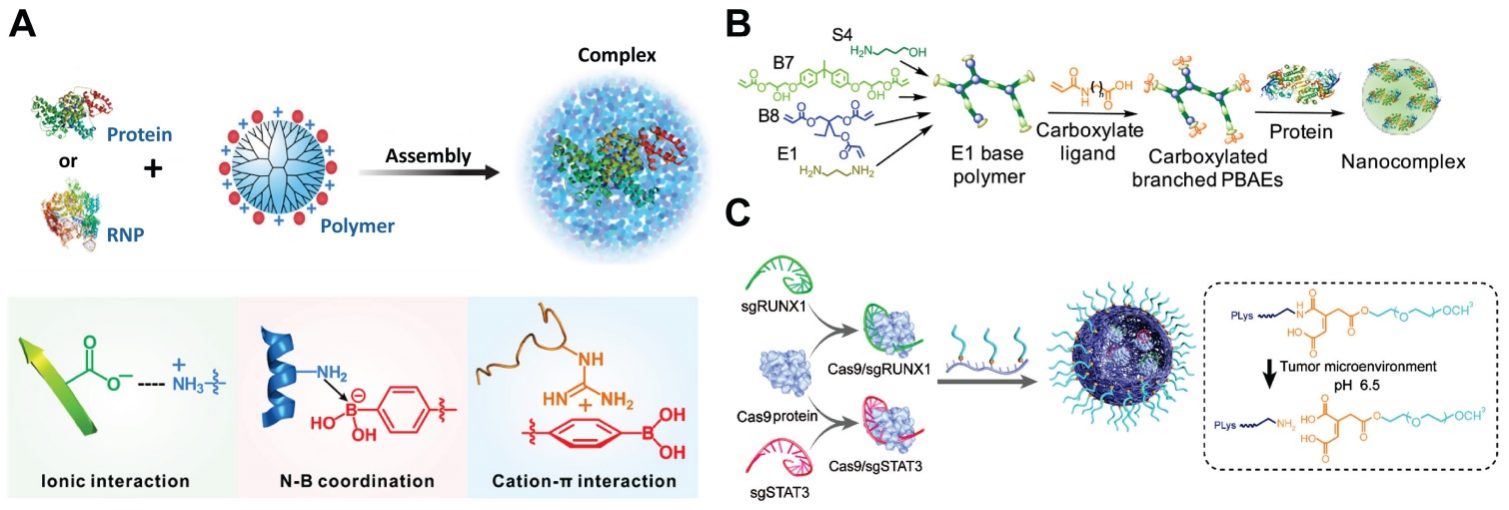
Polymers for Cas9 RNP delivery. **A**. PBA-rich dendrimer used for the intracellular delivery of protein and Cas9 RNP. Adapt with permission from 57. Copyright 2019, The Authors, some rights reserved. Creative Commons CC BY-NC. **B**. Carboxylated branched PBAEs used for the intracellular delivery of protein and Cas9 RNP. Reprinted with permission from 212. Copyright 2019, The Authors, some right reserved. Creative Commons CC BY. **C**. Illustration of the assembly of pH-responsive PEGylated PLL and double targeted Cas9 RNPs. Reduced with permission from 218. Copyright 2019, American Chemical Society.

Dendrimers are a class of synthetic polymers with spherical and hyperbranched structures as well as a high density of surface functional groups 201, 202. These polymers have been widely used as carriers for the delivery of drugs, nucleic acids and proteins 65, 203-206. To ensure efficient RNP binding to the dendrimer scaffold, the polymer was functionalized with a high density of phenylboronic acid (PBA) moieties on the surface 57. PBA is an electron-deficient group that is capable of binding amine and imidazole groups on proteins via nitrogen-boronate complexation 207, 208. The residual amine groups on a generation 5 (G5) polyamidoamine (PAMAM) dendrimer could bind with anionic groups on proteins via electrostatic interactions. Thus, the boronic acid-rich dendrimer complexed with proteins of different isoelectric points to yield uniform nanoparticles (Figure 8A). Because the designed polymer could bind with both Cas9 protein and sgRNA, it efficiently delivered RNP into various cell lines and showed higher editing efficiencies than CRISPR^MAX^ on different target genes. In a separate study, 6-*O*-α-(4-*O*-α-D-glucuronyl)-D-glucosyl-β-cyclodextrin (GUG-β-CD) was conjugated onto a G3 PAMAM dendrimer for RNP-mediated genome editing 209. The synthesized polymer showed genome editing in human neuroblastoma SH-SY5Y cells and in the brain tissue of mouse after intraventricular administration.

#### PBAEs

PBAEs are a class of amphiphilic and pH-sensitive polymers that have been widely used for gene delivery 210, 211. The amine groups on PBAEs can be protonated or deprotonated when the solution pH was below or above the p*K*_b_ values of PBAEs, resulting in the change of polymer hydrophobicity. A hyperbranched PBAE polymer was recently developed for intracellular RNP delivery 212. The polymer was synthesized via a stepwise copolymerization and accomplished via end-capping with carboxylate ligands containing different number of carbon atoms between the amide and carboxylic acid groups (Figure 8B). The PBAEs could efficiently bind cargo proteins via a combination of hydrogen bonding, hydrophobic and ionic interactions. One of the PBAEs terminated with a carboxylate ligand C5 (C5 PBAE) showed the highest efficiency among the synthesized polymers. It could induce 77% GFP knockout in HEK 293T cells and 47% GFP knockout in GL261 murine glioma cells with an indels quantification value of 26%. Furthermore, the co-delivery of RNP and donor ssDNA into HEK 293T cells by C5 PBAE resulted in 4% HDR efficiency and over 50% total editing. In addition, PEAEs/RNP nanoparticles also enabled genome editing *in vivo* using a CRISPR-stop reporter system.

#### PEGylated PLL

The positive charges on cationic polymers usually cause problems when applied *in vivo* due to rapid clearance by the reticuloendothelial system (RES). These polymers were usually modified with biocompatible units such as PEG 213, polyglutamic acid 214, and polysaccharides 215, 216 to shield the positive charges and increase the complex stability *in vivo* 217. PEGylated PLL containing a pH-responsive linker was reported for the delivery of RNP into tumor cells 218. The polymer formed core-shell structured nanoparticles with RNP, and the PEG shell on nanoRNP could be detached under acidic tumor microenvironment which facilitates tumor accumulation and cell internalization (Figure 8C). The nanoRNP targeting activator of transcription 3 (STAT3) achieved 39.1% indels in U87MG cells at pH 6.5, and the polymer nanoparticles targeting both STAT3 and Runt-related transcription factor 1 (RUNX1) efficiently suppressed the proliferation and induced cell apoptosis against the heterogeneous tumors *in vivo*.

#### Chitosan (CS) nanoparticles

CS is a cationic and naturally occurring polymer that has been widely used for biomedical applications due to its bio-adhesive property, low toxicity, and biodegradability. In a recent study, CS was proposed as a polymeric carrier to deliver RNP and donor DNA for HDR 219. Free CS failed to efficiently encapsulate and deliver the Cas9 RNP complexes into cells, and thus a negatively charged red fluorescence protein (RFP) was firstly complexed with CS to prepare RFP@CS nanocomplexes. Cas9 protein fused with twenty glutamate residues at the *N*-terminus and donor DNA were then complexed with RFP@CS to form nanoassemblies. The prepared materials achieved a comparable HDR efficiency to CRISPR^MAX^ in HEK 293 cells with a knock-in frequency of 12.5 ± 3.0%. Cas9 RNP targeting *PRDX4* gene delivered by RFP@CS resulted in 48.7%, 24.4%, 32.6%, 55.8% and 16.9% indels in HEK293T, RAW264.7, HeLa, U2OS and A549 cells, respectively. In addition, RFP in the nanoparticles provides a fluorescent probe to monitor the intracellular RNP delivery. Besides, other polymers such as supramolecular polymers 220 and reduction-sensitive polymers 221-222 were designed for Cas9 RNP delivery, and these materials will be discussed in the section of Responsive delivery systems for Cas9 RNP delivery.

### Nanogels

**Figure 9:**
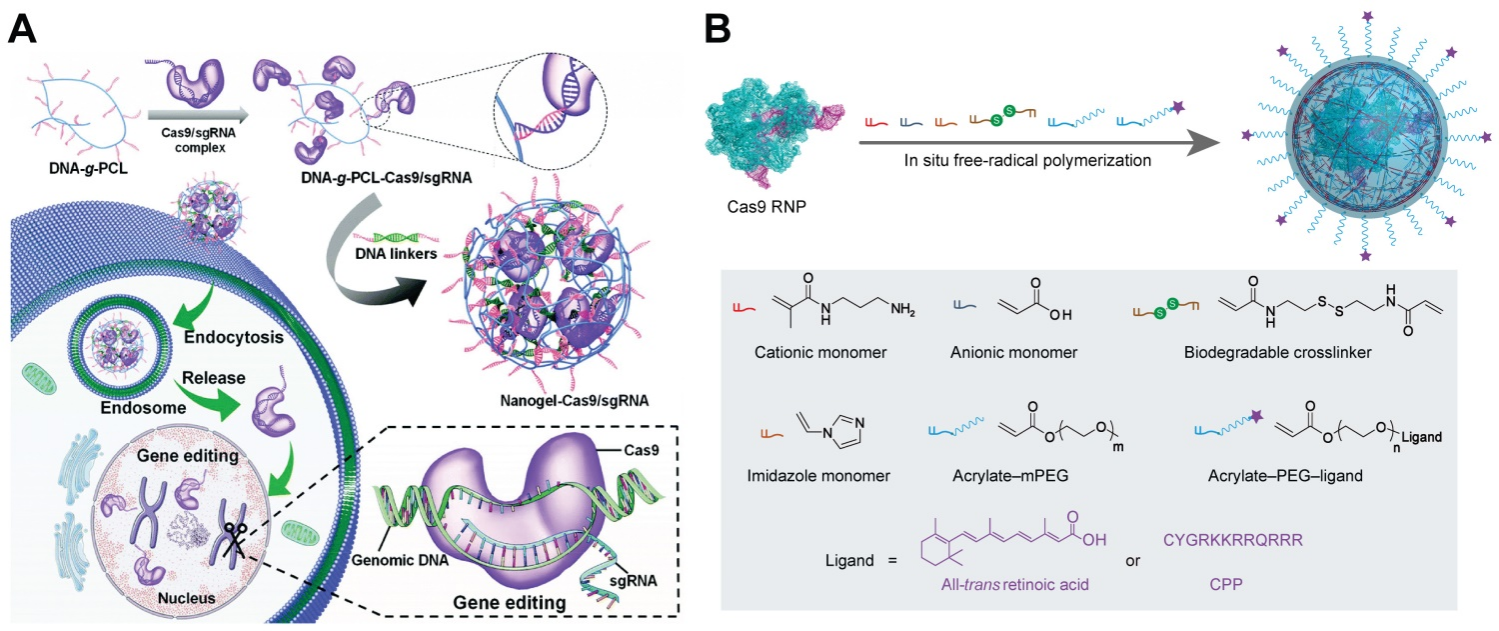
Nanogels for the intracellular delivery of Cas9 RNP. A. Schematic illustration of the RNP-embedded nucleic acid nanogel formation and intracellular delivery. Reduced with permission from 226. Copyright 2019, Royal Society of Chemistry. B. Image of design and preparation of reduction-responsive nanogel for Cas9 RNP delivery.

Nanogels are submicron hydrogels with three-dimensional networks through physical or chemical crosslinking. Owing to their stability, high loading capacity, stimuli responsiveness and biocompatibility, nanogels are promising platform for drug delivery, diagnostics, and catalysis 224, 225. A non-cationic DNA-crosslinked and Cas9 RNP-embedded nanogel was proposed by Zhang et al. for intracellular Cas9 RNP delivery 226. DNA-grafted polycaprolactone brush (DNA-*g*-PCL) was complexed with RNP through complementary base pairing between brushed DNA and sgRNA. The remaining DNA brushes were then crosslinked by DNA linkers via hybridization to form an RNP-embedded nanogel (Figure 9A). The nanogel could protect the embedded Cas9 RNP complex against enzymatic degradation, and induced an indels frequency of 18.7% in HeLa-EGFP cells. Another nanogel formulation for Cas9 RNP delivery was prepared by *in situ* free-radical polymerization of monomers around Cas9 RNP, forming a reduction-responsive nanocapsule with a hydrodynamic diameter around 25 nm 223. Cationic and anionic monomers were coated on the Cas9 RNP through electrostatic interactions, and the other monomers such as imidazole-containing monomer, reduction-sensitive crosslinker, and acrylate PEG were attached to the surface of RNP by hydrogen bonding and van der Waals interactions (Figure 9B). The prepared RNP nanogel induced efficient genome editing, resulting in about 80% mCherry negative HEK293 cells. Further decoration of the nanogel with CPPs can further enhance the genome editing efficiency in HEK293 cells and hESCs. In addition, the nanogel with all-*trans* retinoic acid (ATRA) induced robust genome editing in mouse retinal pigment epithelial (RPE) tissue and skeletal muscles after local administration.

### Inorganic nanoparticles

#### Gold nanoparticles (GNPs)

**Figure 10:**
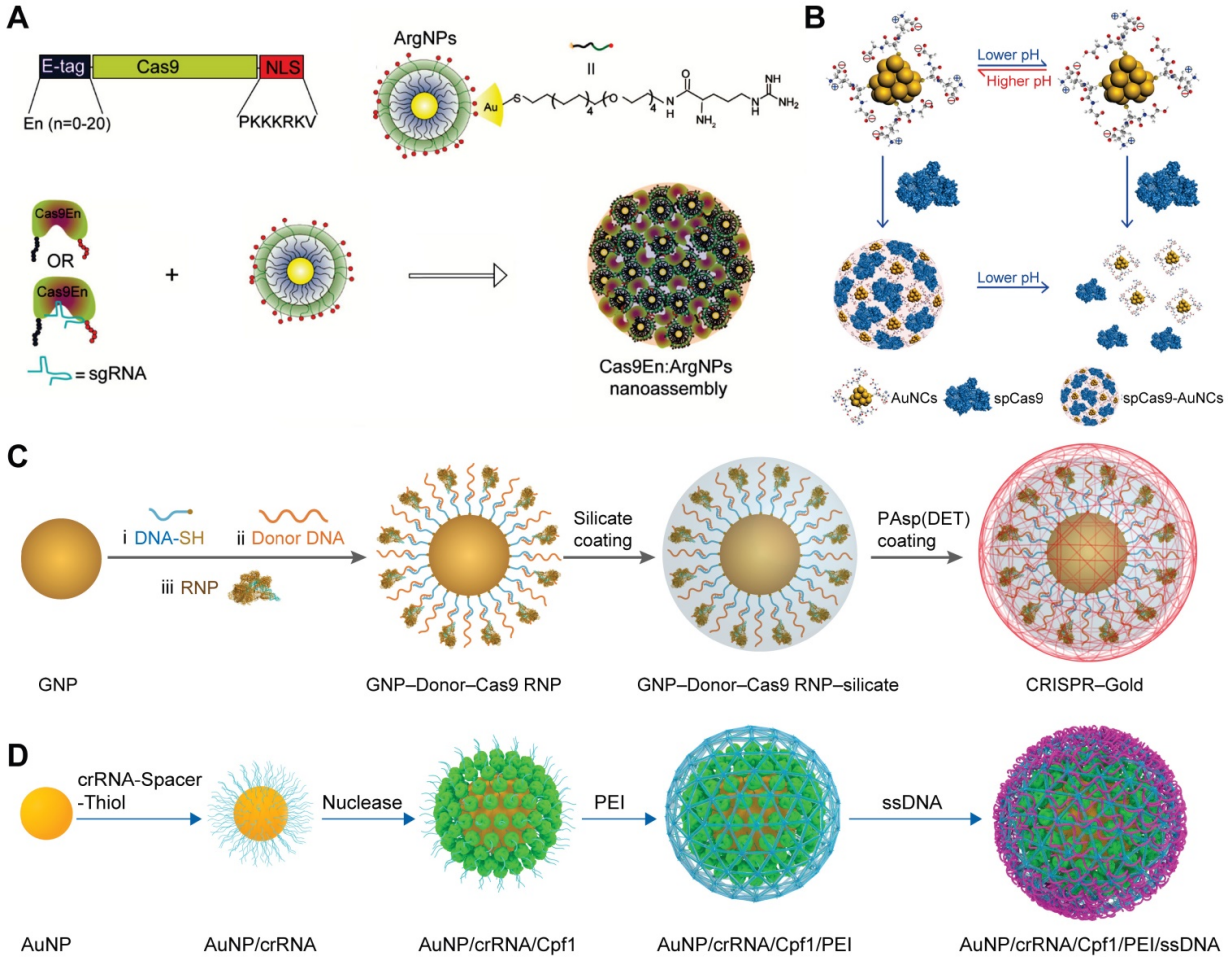
GNP-based delivery platforms for Cas9 RNP. **A**. Rational design of arginine-functionalized GNPs for the intracellular delivery of E-tagged Cas9 or RNP. Adapted with permission from 59. Copyright 2017, American Chemical Society. **B**. Schematic illustration of pH-induced assembly of GSH-modified GNPs with Cas9 protein. Reduced with permission from 233. Copyright 2019, American Chemical Society. **C**. PAsp(DET) coated SNAs for the delivery of Cas9 RNP. **D**. Schematic illustration of GNP-based RNP nanoformulation for genome editing.

Owing to the inherent low-toxicity and the ease of functionalization, GNPs offer a promising platform for the delivery of biomacromolecules 68. Rotello et al. developed a series of arginine-functionalized GNPs (ArgNPs) for cytosolic protein and siRNA delivery 59, 227-229. Since Cas9 is a positively charged protein and may repulse ArgNPs, an anionic glutamate tag (E-tag) was fused to the *N*-terminus of Cas9 protein (Cas9En) to ensure efficient loading of Cas9 RNP to the ArgNPs. (Figure 10A) After careful screening, E-tagged Cas9 with 15 or 20 repeated glutamate units showed optimal co-assembly and intracellular protein delivery. The nanoassemblies of ArgNPs and Cas9E15 RNP showed efficiency of ~30% on both *AAVS1* and *PTEN* genes in HeLa cells. ArgNPs have also been utilized in cancer immunotherapy. CD47 is a cell surface protein overexpressed on most cancer cells to protect themselves from eating by macrophages. The interaction between macrophage signal regulatory protein-α (SIRP-α) and CD47 leads to inhibition of phagocytosis even if phagocytic signals are present. Knocking out *SIRP-α* gene in macrophages via the Cas9E20 RNP/ArgNPs system greatly enhanced the innate phagocytic capability of macrophages by 4-fold 230. Similarly, TAT-functionalized GNPs was developed for the delivery of Cas9 protein and sgRNA encoding plasmid 231. Gold nanoclusters modified with cationic TAT peptide were used to form a ternary complex with Cas9 proteins and sgRNA plasmids via electrostatic interactions, and the ternary complex was further coated with an anionic lipid shell consisting of 1,2-dioleoyl-3-trimethylammoniumpropane (DOTAP), DOPE, and 1,2-distearroyl-*sn*-glycero-3-phosphoethanolamine-PEG (DSPE-PEG), and cholesterol, yielding a hybrid nanoparticle termed LGCP. Polo-like kinase 1 (Plk1) is a highly conserved serine-threonine kinase that is overexpressed in many tumors. Inhibition of Plk1 expression can induce the apoptosis of tumor cells. LGCP induced 26.2% indels at *Plk1* locus and resulted in more than 70% down-regulation of Plk1 protein expression in A357 cells. The inner GNPs could serve as photothermal agents to facilitate the cargoes release in cells under laser irradiation 232. In a separated study, glutathione (GSH) functionalized ultrasmall gold nanoclusters achieved efficient Cas9 protein delivery 233. The assembly and disassembly of nanoclusters and Cas9 protein could be modulated by solution pH. When the pH decreased from 7.4 to 4.5, the amount of negative charges on gold nanoclusters was decreased due to the protonation of carboxylic groups on GSH (Figure 10B). The gold nanocluster/Cas9 protein nanoassemblies achieved an indels frequency of 34% in HeLa cells when sgRNA was transfected by Lipofectamine RNAi^MAX^, resulting in restoration of p53 function and inducing apoptosis in HeLa cells with little effect on normal human cells.

GNPs-based spherical nucleic acids (SNAs) have been widely developed for gene delivery during the past decade 234-243. These anionic charged nanoparticles are highly biocompatible and could be efficiently internalized by cells via scavenger receptor. SNAs could be incorporated with various functional moieties such as template DNA via complementary base pairing with the oligonucleotides on the surface of GNPs. Therefore, GNPs-based SNAs could be used for the co-delivery of Cas9 RNP and template DNA for HDR 244. Thiol-modified oligonucleotide was conjugated onto GNPs via gold-thiol bond and further hybridized with donor DNA. Cas9 RNP was then attached onto GNPs via base-pairing between Cas9 RNP and donor DNA. Following a layer of silica deposited on the nanoparticles to increase the negative charge density, a cationic endosomal disruptive polymer poly(N-(N-(2-aminoethyl)-2-aminoethyl) aspartamide) PAsp(DET) was coated on the complex nanoparticles (Figure 10C). This hybrid nanoparticle, named as CRISPR-Gold, can simultaneously deliver Cas9 RNP and donor DNA into various cells and efficiently correct the DNA mutation both* in vitro* and *in vivo* 245*.* Another study reported a similar material designed for HDR-mediated genome editing in HSPCs 246. crRNA synthesized with an oligo(ethylene glycol) (OEG) spacer and a terminal thiol linker (crRNA-OEG-SH) was attached to the surface of 19 nm GNPs via gold-thiol linkage. The addition of OEG spacer reduced the electrostatic repulsion between crRNA strands, thus increasing the loading capacity of GNPs. Cas9 proteins were attached to the 5ʹ handle of crRNA by the natural affinity of Cas9 protein to the three-dimensional structure of crRNA, resulting in nanoparticles around 22 nm. The RNP-loaded GNPs were further coated with branched low-molecular-weight PEI to load ssDNA template (Figure 10D). The final GNPs possessed an average size of 64 nm. PEI-induced proton sponge effect could promote the escape of GNPs from lysosomes. As a result, the developed material produced up to 17.6% total genome editing with 13.4% HDR at the *CCR5* locus in HSPCs, which were comparable to the results of electroporation-mediated RNP delivery.

#### Metal-organic frameworks (MOFs)

**Figure 11:**
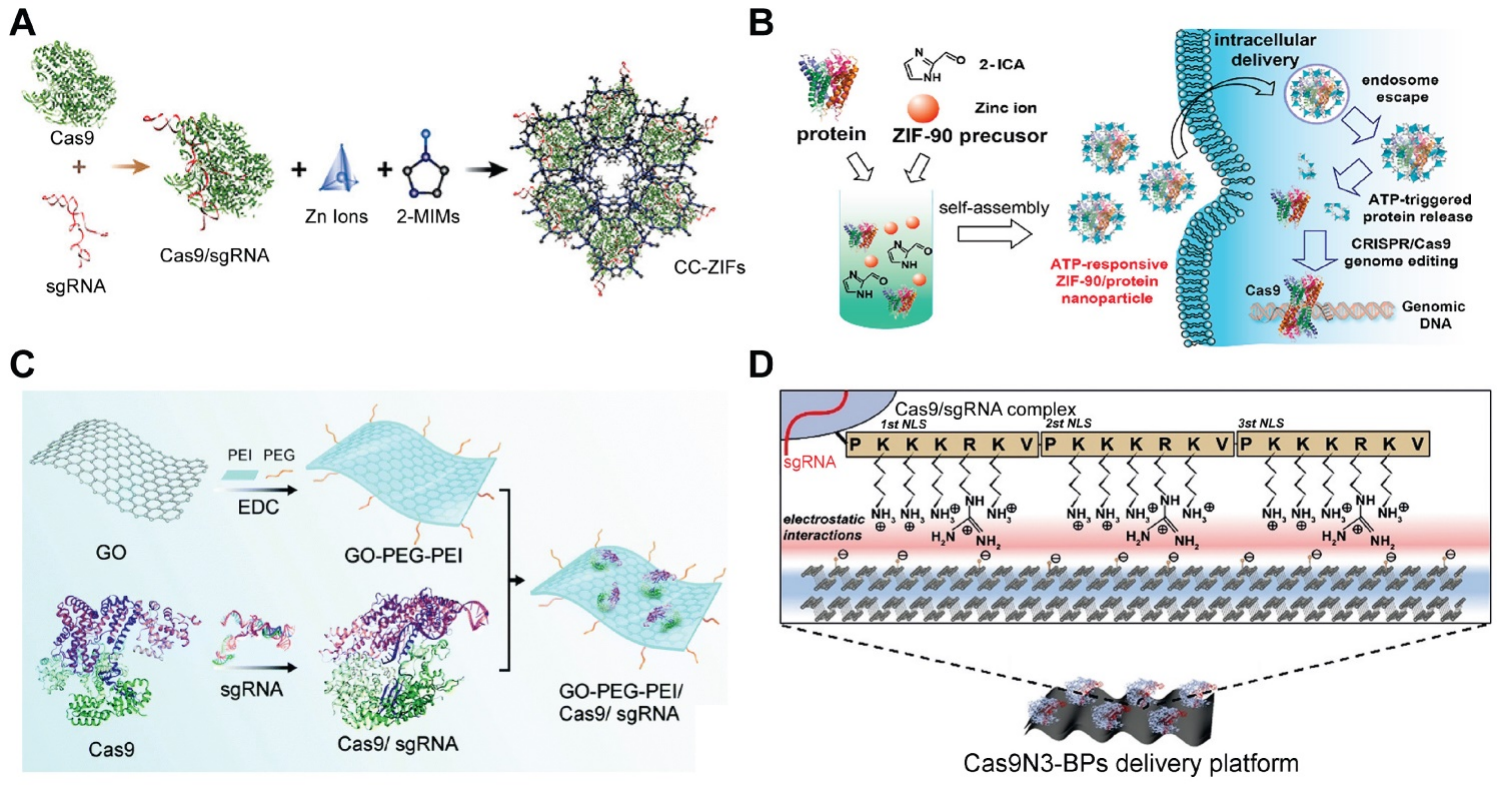
Inorganic materials for the intracellular delivery of Cas9 RNP. **A**. Illustration of the encapsulation of Cas9 RNP into ZIF-8. Reprinted with permission from 249. Copyright 2017, American Chemical Society. **B**. Schematic illustration of the self-assembly and ATP-triggered release of ZIF-90/RNP complex. Reprinted with permission from 251. Copyright 2019, American Chemical Society. **C**. Schematic diagram of the GO-PEG-PEI based Cas9 RNP delivery system. Adapted with permission from 256. Copyright 2018, Royal Society of Chemistry. **D**. Image of the complexation of BP nanosheets and Cas9-3NLS RNPs for genome editing. Adapted with permission from 260. Copyright 2018, Wiley-VCH.

MOFs are organic-inorganic hybrid crystalline porous materials composed of inorganic metal ions and organic molecules 247. Zeolitic imidazolate frameworks (ZIFs) are a class of MOFs comprised of tetrahedrally-coordinated transition metal ions and imidazolate linkers 248. The metal ions in MOFs could interact with proteins or Cas9 RNP via a combination of coordinative and ionic interactions. Up to now, ZIF-8 249, 250 and ZIF-90 251 have been used for intracellular RNP delivery. ZIF-8 is formed by coordination between Zn^2+^ ions and 2-methylimidazole (2-MIM), while ZIF-90 consists of Zn^2+^ and imidazole-2-carboxaldehyde (2-ICA) (Figure 11A-B). Cas9 RNP could be encapsulated in ZIFs during MOF formation. The imidazole moieties in ZIFs may facilitate the endosomal escape of RNP complexes via the pH-buffering mechanism. Furthermore, the competitive binding of Zn^2+^ ions in ZIFs with abundant ATP molecules inside cells is beneficial for intracellular RNP release. ZIF-8/RNP nanoparticles achieved 30% indels targeting *EGFP* in Chinese hamster ovary (CHO) cells and ZIF-90/RNP complexes resulted in ~40% GFP-negative HeLa cells. Hybrid nanoparticles of ZIF and silica also exhibited effective RNP delivery both *in vitro* and *in vivo* 252.

#### Graphene oxide (GO)

GO is a chemically modified graphene containing multiple oxygen functional groups 253. Duo to its excellent cell penetration, high drug loading, optical properties, low toxicity and easy of functionalization, GO has been intensively used as nanotheranostics 254, 255. The large surface area of GO is beneficial for loading biomacromolecules such as proteins. PEG and PEI functionalized graphene oxide (GO-PEG-PEI) was proposed for Cas9 RNP delivery 256. (Figure 11C) The complexation of GO-PEG-PEI with RNP yielded ~220 nm nanoparticles through physical adsorption, π-stacking and ionic interactions. PEI modified on the GO contributed to efficient endosomal escape, and the GO-PEG-PEI successfully delivered Cas9 RNP into human AGS cells with a genome editing efficiency of ∼39%.

#### Black phosphorus (BP) nanosheets

BP nanosheets are a new class of two-dimensional (2D) materials with a natural bandgap that holds unique anisotropy and extraordinary physical properties 257-259. As a stable allotrope of elemental phosphorus, BP nanosheets have excellent element biocompatibility and can be degraded into low toxic phosphite/phosphate ions under physiological conditions. In addition, there are periodic atomic grooves on surfaces of BP providing ideal anchoring sites for protein loading. Taking advantages of extraordinary physical properties, BP nanosheets were employed as a biodegradable platform for Cas9 RNP delivery 260. The Cas9 protein were fused with three NLSs at C-terminus to enhance its electrostatic interaction with BPs and improve the nuclear transportation of Cas9 RNP after internalization (Figure 11D). Due to the enhanced electrostatic interaction provided by NLSs and the 2D puckered honeycomb structure of BP nanosheets, the material exhibited a remarkable Cas9 RNP loading capacity of up to 98.7%. The complexes of BP and Cas9 RNP were delivered into cells by direct membrane penetration and endocytosis pathways. The degradations of BP in the acidic vesicles promoted endosomal escape and intracellular Cas9 RNP release. As a result, Cas9 RNP delivered by BP nanosheets induced indels frequencies of 32.1% in human breast carcinoma MCF-7 cells, 22.8% in human bone marrow derived mesenchymal stem cells (hBMSCs), and 17.2% in mouse macrophage RAW264.7 cells.

#### Calcium phosphate nanoparticles

Calcium phosphate nanoparticles are usually used as non-viral vectors for gene therapy due to their biocompatibility and strong binding affinity with nucleic acids 261, 262. The Cas9 RNP complexes were *in situ* mineralized by calcium phosphate under physiological conditions 263. Calcium phosphate mineralization efficiently increases the RNP stability and cell internalization with maintained the bioactivity. The mineralized Cas9 RNP nanoparticles were efficiently delivered into protoplast cells of a model plant pathogenic fungus, *Magnaporthe oryzae*, and achieved 20% Scytalone dehydratase genome editing efficiency.

### DNA nanoclews

**Figure 12:**
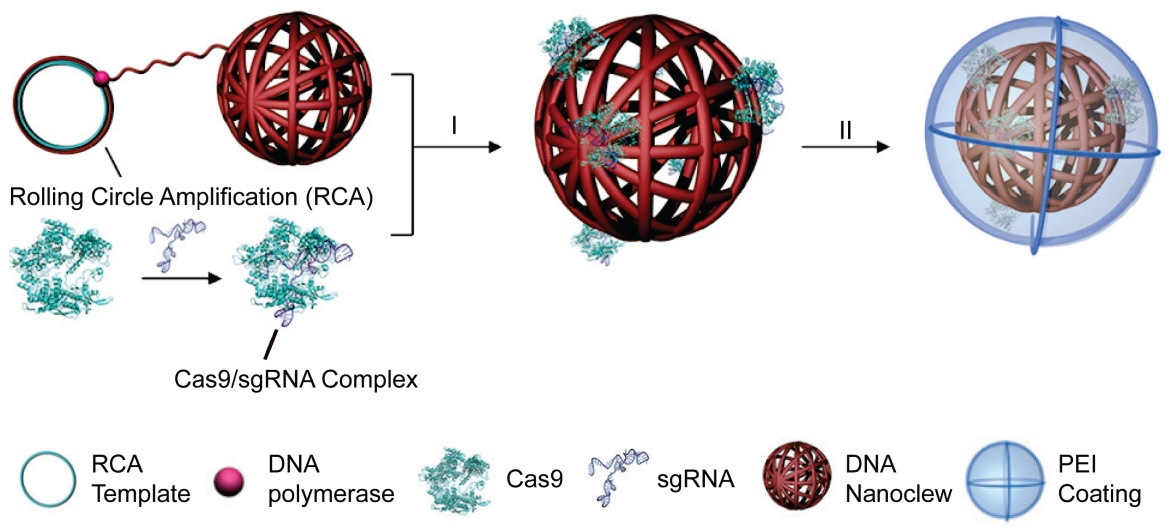
DNA nanoclews for the delivery of Cas9 RNP. Adapted with permission from 266. Copyright 2015, Wily-VCH.

DNA nanoclews are a class of nucleic acid nanostructures synthesized by rolling circle amplification (RCA). Because of its high biocompatibility, predictability, programmability and simplicity to functionalization, DNA nanoclews have been developed as vehicles for drug delivery 264 and gene delivery 265. A yarn-like DNA nanoclew was synthesized by RCA with palindromic sequences encoded to be partially complementary to sgRNA in the RNP complex. After complexation of DNA nanoclews with RNP, a cationic polymer PEI was coated on the nanoclew to facilitate its cellular uptake and endosomal escape 266. (Figure 12) DNA nanoclews with 12 nucleotides complementary to sgRNA (NC-12) resulted in a higher genome editing efficiency than NC-0 and NC-23, which may be attributed to the balanced RNP binding and intracellular release via complementary base pairing. The NC-12/RNP/PEI complexes induced 25% gene disruption of the U2OS cells in the frozen tumor sections around the intratumoral injection site. A hepatocyte-targeted charge reversal polymer was coated on RNP nanoclews for targeted delivery of Cas12a/crRNA RNP *in vivo* 267. Similarly, a polymeric sgRNA/siRNA nanoparticle was prepared by rolling circle transcription for intracellular RNP delivery 268. The siRNA was incorporated as a Dicer substrate sequence that would induce endogenous specific ribonuclease to cleave the double-strand RNA for RNP release. The polymeric sgRNA/siRNA was loaded with Cas9 protein and further co-formulated with cationic lipids to prepare RNP nanoparticles. Such polymeric RNP nanoparticles were more stable than monomeric RNP and showed high serum stability during genome editing. The lipid-encapsulated polymeric RNP resulted in more than 60% indels frequency in HeLa cells. The *in vivo* gene disruption assay also showed that poly-RNP with Dicer siRNA can cause higher gene disruption than mono-RNP.

## Responsive delivery systems for RNP delivery

Responsive delivery systems provide several benefits for genome editing such as improved editing efficiency and reduced off-target effects. RNP delivery materials responsive to external stimuli such as light and ultrasound can initiate genome editing with precise spatiotemporal control, which is critical for *in vivo* genome editing applications. In addition, materials responsive to endogenous triggers such as pH, redox potential, enzymes and ATP may promote intracellular release of RNP molecules and increase editing efficiency. These responsive RNP delivery systems are discussed in detail below.

### Light-responsive materials

**Figure 13:**
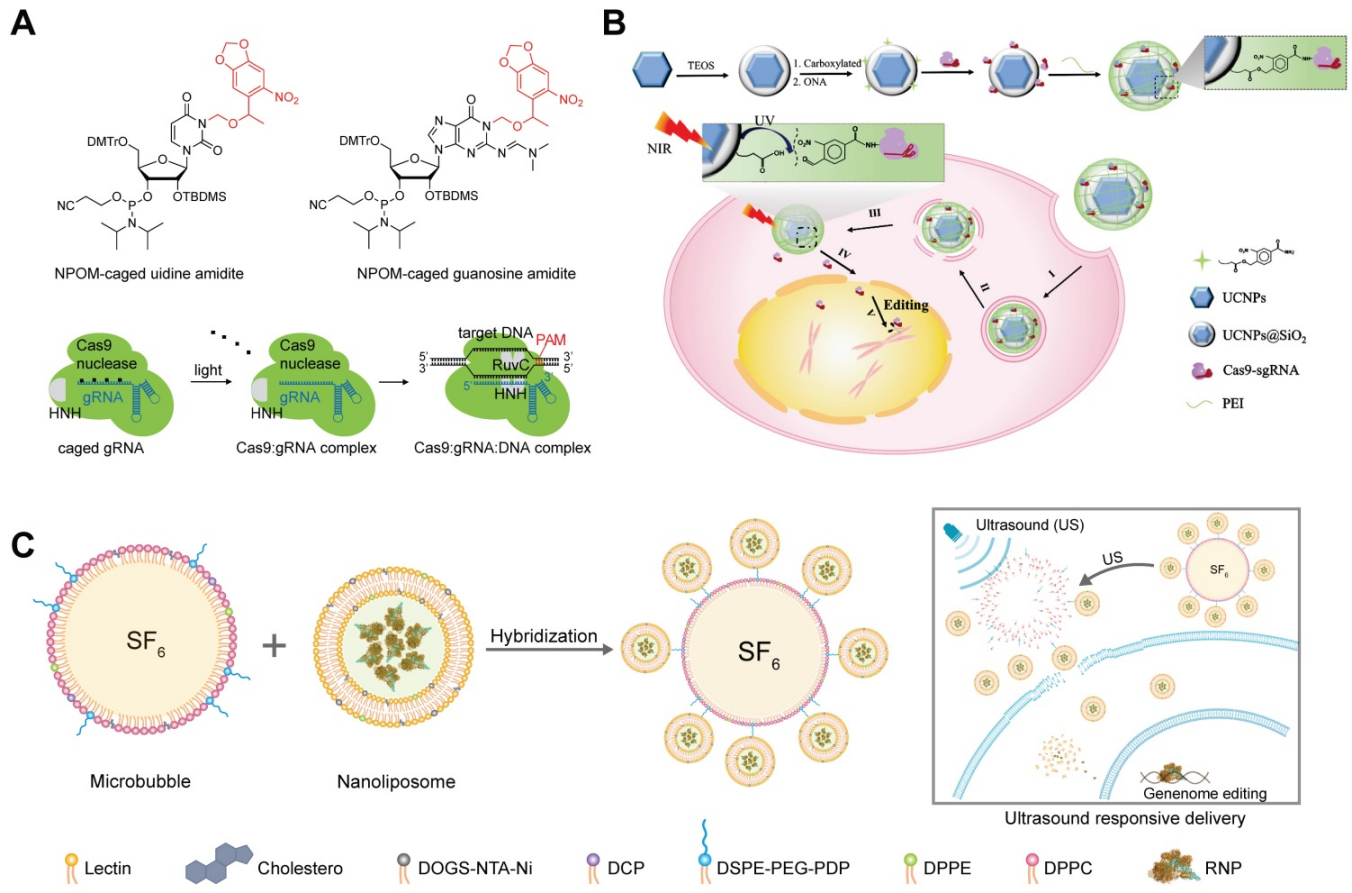
Responsive delivery systems for RNP delivery. **A**. NPOM-caged sgRNA for spatiotemporal control of Cas9 RNP function. Adapted with permission from 269. Copyright 2020, Wiley-VCH. **B**. UCNP-based NIR-responsive Cas9 RNP delivery system. Reduced with permission from 272. Copyright 2019, The Authors, some rights reserved. Creative Commons CC BY-NC. **C**. Schematic illustration of US-activatable microbubbles as Cas9 RNP delivery system for androgenic alopecia therapy.

Researchers developed a photocaged sgRNA to regulate the interactions between RNP and dsDNA and demonstrated the feasibility of light-activatable genome editing in zebrafish embryos 269. By replacing normal nucleobases with 6-Nitropiperonyloxymethylene (NPOM)-caged nucleobases within the protospacer region of sgRNA, the formation of RNP/dsDNA complex was inhibited until the restoration of base-pairing capability of sgRNA via ultraviolet (UV) light-mediated photolysis (Figure 13A). The Cas9/caged sgRNA RNP complex was delivered into cells by Lipofectamine 3000, and off-on switching of genome editing function was successfully controlled by UV light exposure.

Though UV light-responsive materials showed promising features in cell level studies, the applications of such systems *in vivo* are hindered by the poor tissue penetration of UV light and its phototoxicity. Upconversion nanoparticles (UCNPs) are anti-Stokes type materials in which rare earth atoms are embedded in a crystalline matrix. UCNPs can convert near-infrared (NIR) light radiation with lower energy to visible or UV light 270, 271. A recent study reported a NIR light-responsive genome editing nanoparticle based on UCNPs and photo-cleavable ligands 272. To improve the water solubility and biocompatibility, a silica shell was coated on the surface of UCNPs, and then Cas9 RNP complexes were conjugated to the UCNPs@SiO_2_ by using an UV-cleavable 4-(hydroxymethyl)-3-nitrobenzoic acid (ONA) linker to obtain a light-cleavable Cas9 conjugate (UCNPs-Cas9). Finally, a cationic polymer PEI was coated on the UCNPs-Cas9 to facilitate cellular uptake and endosomal escape (Figure 13B). The UCNPs-Cas9 nanoparticles could efficiently release Cas9 RNP after intracellular delivery controlled by an NIR light. As a result, the developed nanoparticles achieved on-demand release of Cas9 RNP and reduced off-target effects. By using this strategy, the proliferation of tumor cells was successfully inhibited via NIR light-activated genome editing both *in vitro* and *in vivo*.

### Ultrasound (US)-responsive materials

US-activatable microbubbles were incorporated with lipid nanoparticles for spatiotemporally controlled RNP delivery 273 (Figure 13C). The lipid nanoparticles consisting of cholesterol, lecithin, 1,2-dipalmitoyl-*sn*-glycero-3-phosphoethanolamine (DPPE), and DOGS-NTA-Ni was prepared by a thin-film hydration method, and further loaded with His-tagged Cas9 RNP via metal affinity between immobilized Ni ions and His-tag. A cationic polymer PEI was further added for charge compensation, and the Cas9 RNP encapsulation efficiency was improved from 42% to 82%. The nanoparticles were further conjugated to microbubbles brimming with sulfur hexafluoride (SF_6_) via a disulfide linkage. The yielding microbubbles effectively facilitated local delivery of RNP complex upon ultrasound activation, resulting in spatiotemporally controlled genome editing. Steroid type II 5-alpha-reductase (SRD5A2) is an enzyme that converts testosterone into dihydrotestosterone, which may cause the damage of dermal papilla cells (DPCs) and hair loss. US-activated microbubbles induced an indel frequency of 67.1% on *SRD5A2* gene in DPCs under US treatment. Cas9 RNP targeting mouse *SRD5A2* gene was successfully delivered into DPCs of androgenic alopecia mice via microbubble cavitation, and the treatment successfully recovered hair growth *in vivo*.

### Reduction-sensitive materials

**Figure 14:**
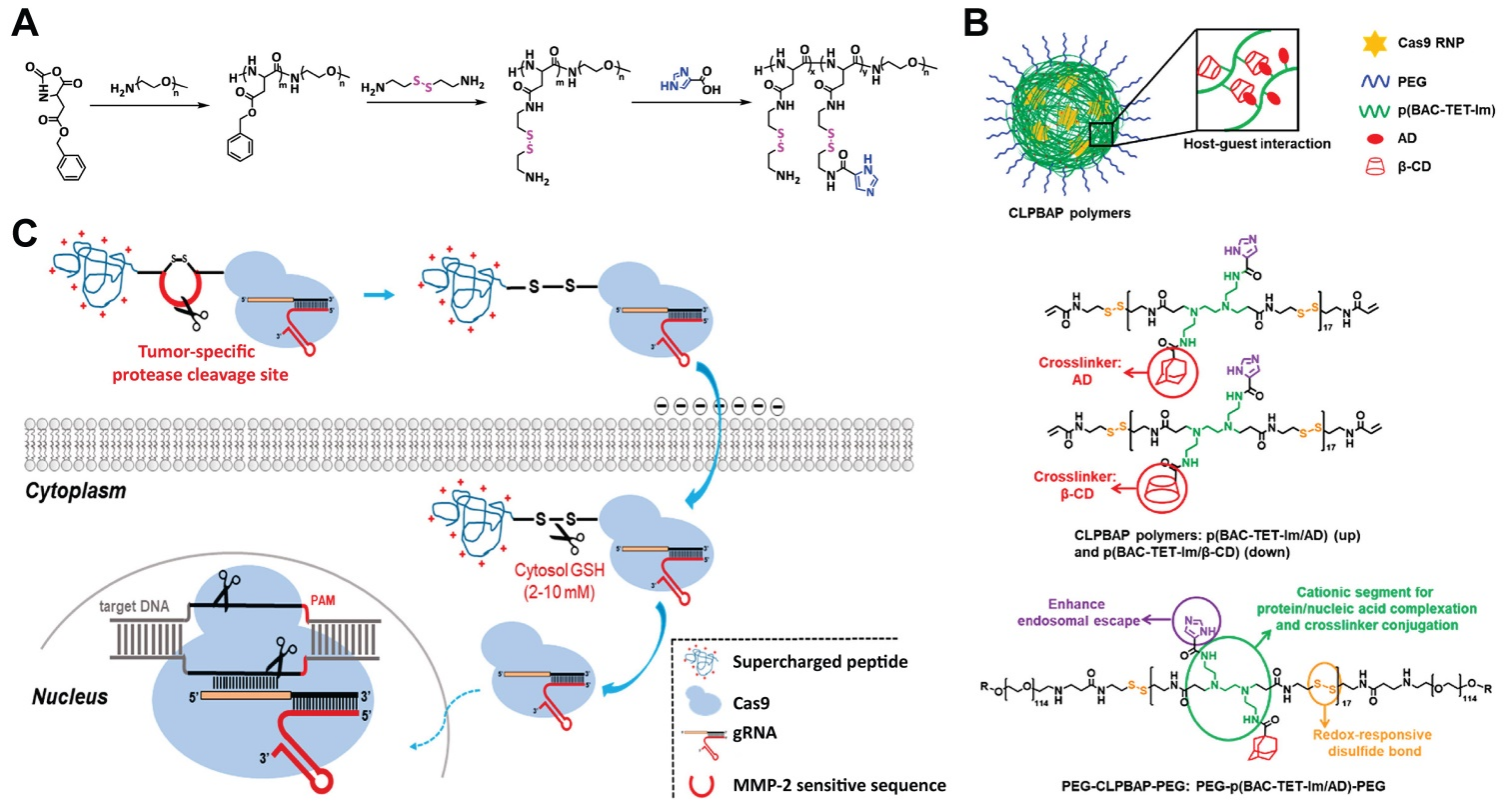
Reduction-sensitive Cas9 RNP delivery systems. **A**. Synthesis of GSH-responsive cationic block copolymer for the delivery of CRISPR/Cas9 system. Reduced with permission from 221. Copyright 2018, American Chemical Society. **B**. Redox-responsive cross-linked polymers for the delivery of Cas9 RNP. Adapted with permission from 222. Copyright 2018, American Chemical Society. **C.** Schematic illustration on microenvironment-responsive delivery of Cas9 RNP. Reduced with permission from 179. Copyright 2019, American Chemical Society.

Disulfide bond containing materials are responsive to GSH. Since the intracellular GSH concentration is much higher than the extracellular one, the use of disulfide bond containing materials for intracellular delivery of biomacromolecules could efficiently release the bound cargoes after cell internalization. A cationic block copolymer, poly(aspartic acid-(2-aminoethyl disulfide)-(4-imidazolecarboxylic acid))-PEG (P(Asp-AED-ICA)-PEG) was synthesized for Cas9 RNP delivery. The polymer could efficiently complex with RNP and showed genome editing efficiency comparable to that of Lipofectamine 2000 (Figure 14A) 221. PEG chains on the polymer offers a neutral shell and thus enhances the stability of RNP complexes, while the imidazole residues enable a rapid endosomal escape behavior. Once delivered into cytosol, the disulfide linkage in the polymer could be cleaved by GSH, degrading the polymer into segments and releasing loaded Cas9 RNP molecules. In a separate study, they synthesized several polymers containing disulfide bonds in the backbone and imidazole groups on the side chains 222. The polymers were cross-linked into nanoparticles through the host-guest interaction between adamantane (AD) and β-cyclodextrin (β-CD) (Figure 14B). The developed polymers successfully induced NHEJ- and HDR-mediated genome editing and maintained high stability in the presence of polyanions. In further studies, they adapted this “cross-linked” strategy to deliver Cas9 RNP *in vivo* by a GSH-cleavable polymer coating 223. SCP-Cas9 fusion protein complexed with sgRNA were proved to be effective in genome editing 178. By ulteriorly connecting SCP and Cas9 protein by a dithiocyclopeptide containing MMP-2 sensitive sequence and an intramodular disulfide bond, the yielding Cas9-linker-SCP RNP could induce higher genome editing efficiency in tumor cells compared with normal cells (Figure 14C). This is due to the cleavage of the linker by MMP-2 in the extracellular matrix of tumor and the disulfide bond by intracellular GSH, leading to the efficient release of Cas9 protein 179.

### pH-responsive materials

pH-responsive materials can respond to solution pH by undergoing structural and property changes, such as surface activity, solubility, chain conformation, and configuration 274. The pH-responsive materials are typically designed using ionizable acidic or basic residues. The structural and property changes depend on selective protonation and deprotonation of these weak acidic/basic pendant groups. The pH value of extracellular fluid is kept constant at 7.4 while the cellular cytosol is at 7.2. In addition, the pH values of most solid tumors (6.5-7.2) are lower than normal tissues, and the pH values of endosome and lysosome are maintained at a much lower level, which are ~6.3 for early endosome, ~5.5 for late endosome, and 4-5 for lysosome 275. The pH-responsive materials can be designed according to these physiological differences. In the intracellular delivery of Cas9 RNP complexes, pH-responsive materials are widely used to facilitate endosomal escape and intracellular release. An amphiphilic molecule FEDS was formulated with lipids to disrupt the endosomal membrane as introduced earlier (Figure 6C) 176. The pH-sensitive polymer PBAEs (Figure 8B) 212, PEGylated PLL with a pH-responsive linker (Figure 8C) 218, and GSH-functionalized GNPs (Figure 10B) 233 are also used for intracellular Cas9 RNP as described above.

## Targeted delivery systems for RNP delivery

### Galactose-based targeting

**Figure 15:**
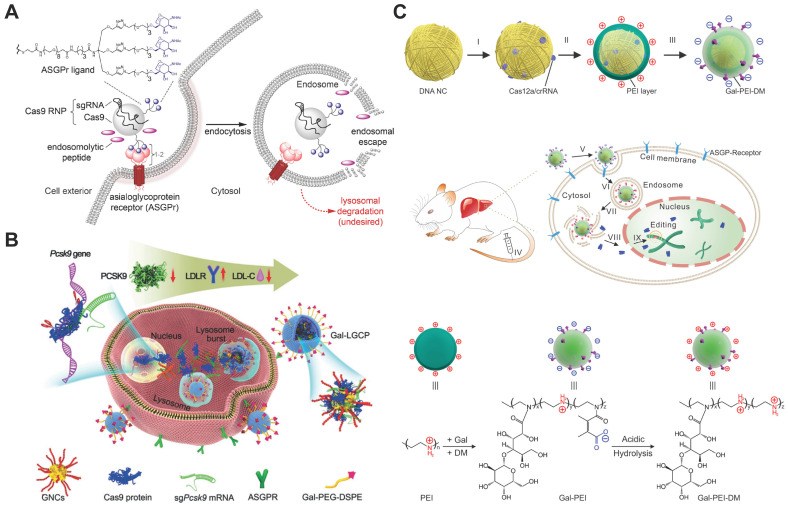
Gal-mediated targeted Cas9 RNP delivery. **A**. Receptor-mediated delivery of Cas9 RNP. Reduced with permission from 276. Copyright 2018, American Chemical Society. **B**. Schematic diagram of Gal-conjugated gold nanoclusters for Cas9 RNP delivery. Reduced with permission from 278. Copyright 2019, Wiley. **C**. Schematic illustration of the Gal-targeted PEI nanoparticles for genome editing. Adapted with permission from 267. Copyright 2020, The Authors, some rights reserved. Creative Commons CC BY-NC.

Targeted RNP delivery is critical for the translation of genome editing technologies into medicine. Targeted genome editing can be achieved by the specific recognition between ligand and related receptors overexpressed on target cells. For example, Cas9 RNP decorated with galactose (Gal) enabled selective delivery into human hepatocytes overexpressing asialoglycoprotein receptor (ASGPr) via a receptor-mediated endocytosis mechanism (Figure 15A) 276, 277. The endosomal escape of the RNP complexes were promoted by the addition of an endosomolytic peptide ppTG21. Subsequent nuclear localization was then induced by the NLS sequence on Cas9 protein, and finally realizing efficient genome editing in target cells. Similarly, TAT-modified gold nanoclusters were loaded with RNP, and further coated with a lipid shell bearing 4-aminophenyl β-D-galactopyranoside on the surface for targeted delivery (Figure 15B). The targeted nanoparticles showed an *in vitro* genome editing efficiency of ~60% and a reduction of ~30% plasma LDL-C in mouse after treatment 278. By modifying PEI with Gal and 2,3-dimethylmaleic anhydride (DM), a charge reversal polymer Gal-PEI-DM was synthesized and coated on the surface of DNA nanoclews for *in vivo* delivery of Cas12a/crRNA RNP 267. The negatively charged Gal-PEI-DM layer on nanoclews enables long blood circulation and selective hepatocyte uptake. The acidic endosomal environment could trigger the charge conversion of the nanoclews, facilitating the escape of Cas12a/crRNA RNP from endosomes (Figure 15C). Proprotein convertase subtilisin/kexin type 9 (PCSK9) is a liver-secreted protease that degrades low-density lipoprotein receptor (LDLR), a key receptor that mediates the endocytosis of cholesterol. The targeted nanoclews delivering Cas12a/crRNA achieved 75% indel formation and induced efficient Pcsk9 disruption in 3T3-L1 cells *in vivo* (~48% by deep sequencing). The nanoclews-mediated genome editing led to ~45% cholesterol reduction after treatment.

### RGD-based targeting

**Figure 16:**
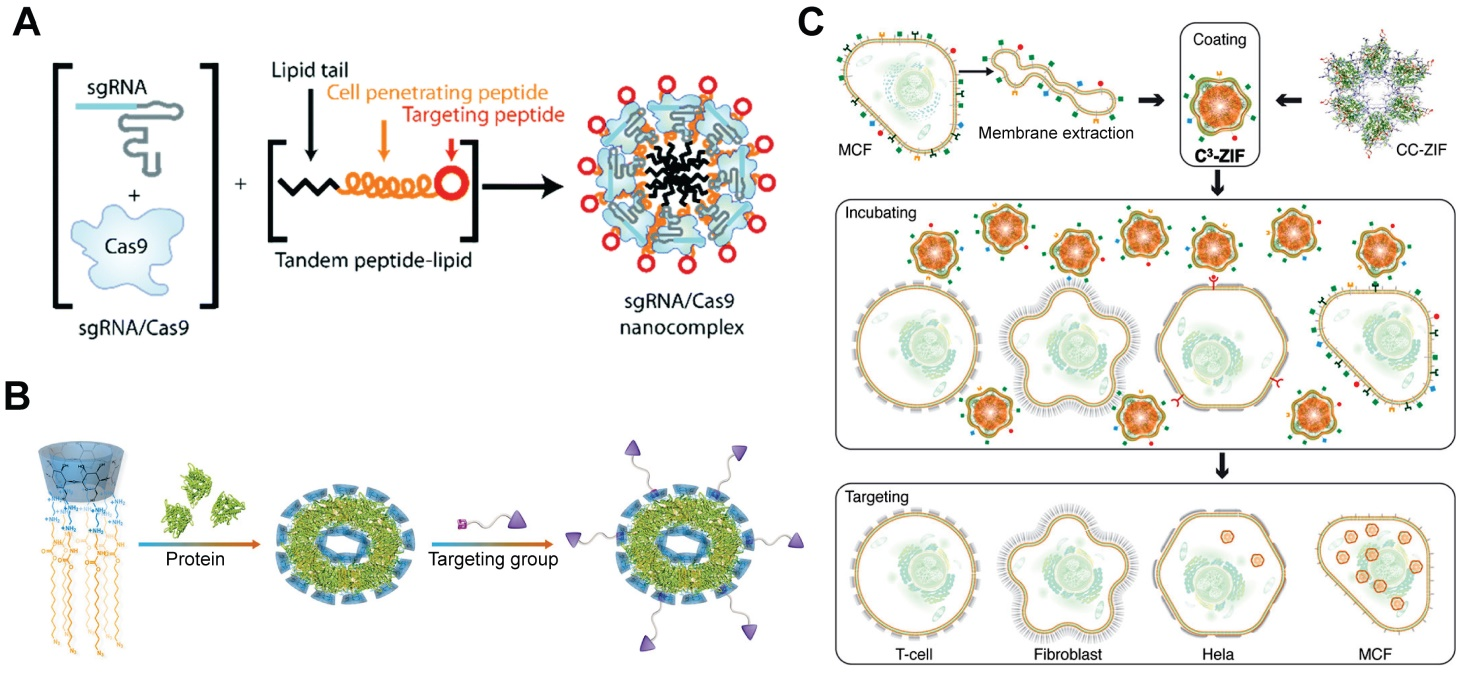
Targeted delivery systems for Cas9 RNP delivery.** A**. iRGD-containing lipopeptide for targeted Cas9 RNP delivery. Reduced with permission from 191. **B**. Folate-based targeted delivery system for Cas9 RNP delivery. Reduced with permission from 280. Copyright 2019, Wiley-VCH. Copyright 2019, Royal Society of Chemistry. Creative Commons BY-NC. **C**. Schematic illustration of the cell-specific delivery system. Reduced with permission from 250. Copyright 2020, American Chemical Society.

Tripeptide RGD (Arg-Gly-Asp) peptide has high binding affinity with integrins α_v_β_3_, which are overexpressed on most cancer cells. An RGD analogue (iRGD) with high affinity to integrins and neuropilin-1 was used to develop cancer-targeted RNP delivery systems. The iRGD-containing tandem lipopeptide, palmitoyl-TP-iRGD, was co-assembled with Cas9 RNP for targeted RNP delivery (Figure 16A). The targeted lipopeptide exhibited higher efficiencies than Lipofectamine RNAi^MAX^ in various cell lines 191. In a separate study, nanoparticles consisting of PEI-CD and PEI-AD were used for RNP delivery and the particles were further coated with DOTAP lipids bearing two peptides 279. iRGD and a CPP mHph3 were conjugated on the nanoparticles for targeted RNP delivery. The Cas9 loading efficiency of DOTAP lipids was increased from 6.3% to 62.8% when the PEI-CD/PEI-AD nanoparticles were used. The iRGD-containing nanoparticles showed efficient genome editing in human brain tumor U87 cells and GS5 cells on* PLK1* gene, and effectively inhibited tumor growth *in vivo*.

### Other ligand based targeting

Targeting ligands could be decorated onto RNP delivery systems via host-guest recognition. For example, an amphiphilic β-CD modified with multiple hydrophobic chains were co-assembled with cargo proteins or RNP into nanoparticles 280, and the assembled materials were modified with targeting ligands such as AS1411 aptamer targeting nucleolin receptors or folate with high binding affinity to folate receptors via β-CD/AD host-guest chemistry. The aptamer-targeted nanoparticles could efficiently deliver cargoes into MDA-MB-231 breast tumor with overexpressed nucleolin receptors, and folate-decorated nanoparticles delivering RNP targeting *Plk1* exhibited significant gene disruption and tumor growth inhibition *in vivo* (Figure 16B). In a separate study, a disulfide-bridged guanidyl AD was complexed with β-CD-conjugated low-molecular-weight PEI to form a supramolecular polymer via β-CD/AD host-guest interaction 220. A biocompatible and negatively charged hyaluronic acid (HA) was used to shield the positive charge on the polymer/RNP complexes, and the hyaluronic acid-coated nanocomplexes efficiently edited mutant *KRAS* in colorectal cancer cells, and inhibited tumor growth and metastases *in vivo*.

MOF nanoparticles such as ZIF-8 249 and ZIF-90 251 have shown high efficiency in Cas9 RNP delivery. Coating ZIFs/RNP nanoparticles with cancer cell membranes endows these nanoparticles with cell-selective properties 250. After incubation with various types of cells, ZIFs/RNP nanoparticles coated with MCF-7 cell membranes showed the highest uptake by MCF-7 cells but negligible uptake by normal cells (Figure 16C). *In vivo* experiments further demonstrated selective accumulation of membrane-coated ZIF/RNP nanoparticles in MCF-7 tumors. ATRA is a targeting ligand that can bind to the inter-photoreceptor retinoid-binding protein (IRBP), a major protein in the inter-photoreceptor matrix that selectively transports all-*trans*-retinol to the RPE and 11-cis-retinal to photoreceptor outer segments. Hybrid ZIF/silica nanoparticles was coated with ATRA for targeted genome editing 252, and the hybrid nanoparticles induced efficient genome editing in mouse RPE after subretinal injection. In a similar study, ARTA-modified nanocapsule with reduction-responsiveness also induced efficient genome editing in RPE cells *in vivo* 223.

### Selective organ targeting (SORT)

**Figure 17:**
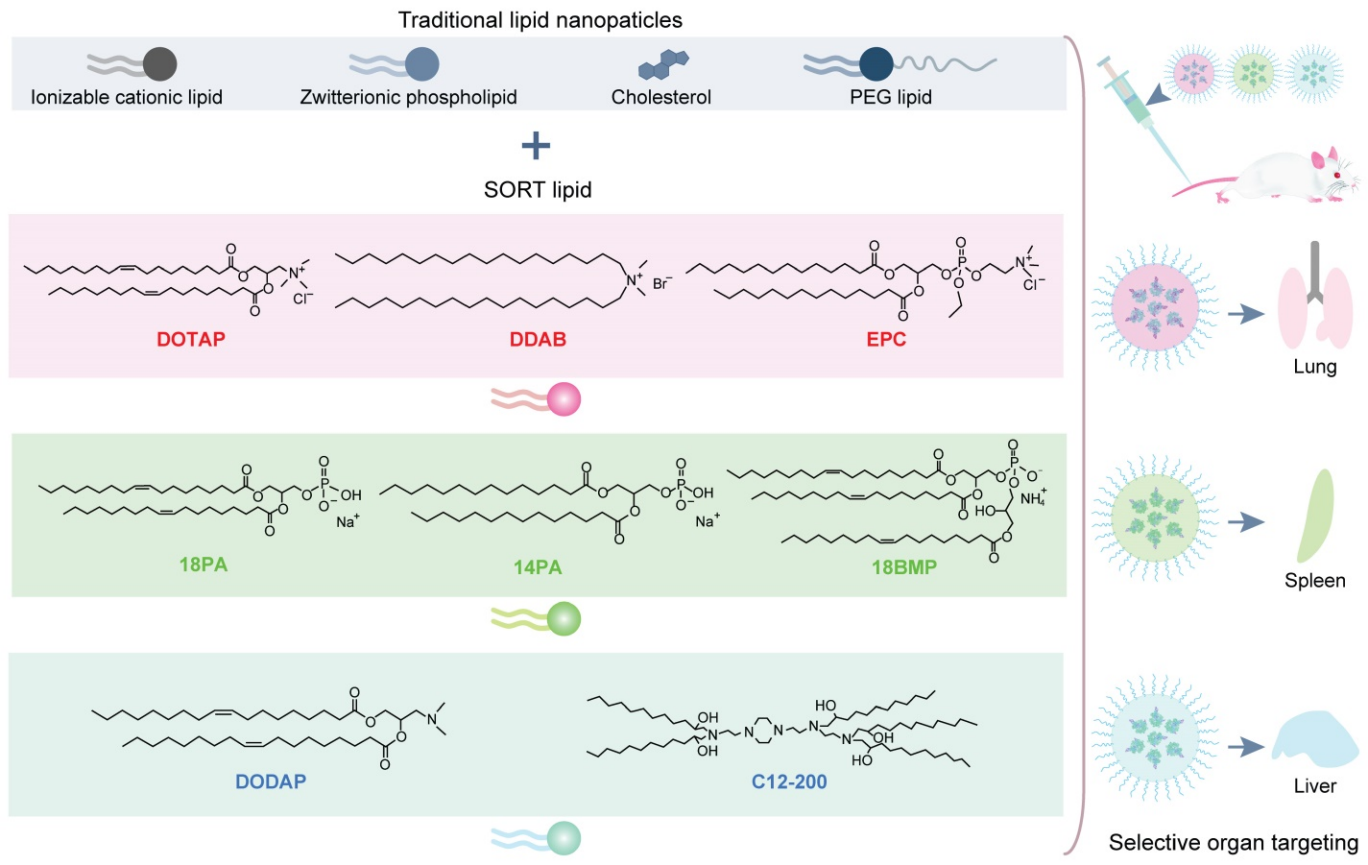
Selective organ targeting systems for the delivery of CRISPR/Cas9 system 281.

Besides active targeting, researchers recently reported a novel strategy termed SORT for CRISPR/Cas9 delivery 281. They found that the regulation of components in lipid nanoparticles can achieve tissue-specific delivery (Figure 17). Lipid materials including ionizable cationic lipid, zwitterionic phospholipid, cholesterol and PEGylated lipid and a fifth SORT molecules were used to prepare the lipid nanoparticles. Different percentages of permanently cationic (defined as positively charged without pKa or pKa > 8), anionic, zwitterionic, and ionizable cationic lipids resulted in a series of lung-, spleen- and liver-targeted SORT lipids, which mediated tissue-specific genome editing in tdTom transgenic mice and C57/BL6 wild-type mice via the delivery of Cas9 RNP.

## Conclusions and perspectives

Genome editing has entered a blooming period of development in recent years due to its extensive and effective application promise for scientific researches and disease treatment 282, 283. The widely-used CRPSPR/Cas9 system is by far the most flexible and convenient genome-editing tool. In the past few years, the revolutionary CRISPR/Cas9 system showed the great promise of treating genetic disorder-induced diseases, monogenic diseases, cancers, cardiovascular diseases, inflammations, neurodegenerative diseases, infectious diseases, and some of these applications have entered clinical trials (Table 1). What's more, the use of programmable nucleases for targeted mutagenesis of plants is advancing rapidly and has great potential for the next generation of plant breeding. It is worth noting that the CRISPR/ Cas9 system paves the way for the development of rapid and cost-effective ways to create new mutant populations in plants. And the CRISPR/ Cas9 system, which can edit plant genomes without introducing foreign DNA into cells may alleviate regulatory concerns related to genetically modified plants. Development of safe and effective delivery systems is the primary issue for the applications of CRISPR/Cas9 systems. Direct delivery of the CRISPR/Cas9 RNP takes advantages in transient function, higher genome-editing efficiency and lower off-target effect when compared with the delivery of Cas9 plasmid and mRNA. To date, a variety of RNP delivery systems such as physical approaches and synthetic carriers have been developed (Table 2). Physically-induced intracellular delivery are mainly mediated by membrane disruption 67. Physical approaches like microinjection, electroporation, microfluidics, nanotube spearing, biolistics, iTOP, and PEG-mediated transformation have been used for cytosolic delivery of macromolecules for decades. Microinjection, electroporation and microfluidics can generally achieve high RNP delivery efficiency and are suitable for almost all cell types but might induce cell damages and require highly skilled manipulation and/or expensive equipment. Meanwhile, biolistic and PEG-mediated transformation are two proven technologies mainly applied on plant cells. The action mechanisms and defects of these physical approaches critically limit their clinical applications *in vivo*. iTOP is a novel delivery method for native proteins and Cas9 RNP, but it is also difficult to be used for therapeutic purpose.

The development of carriers for RNP delivery is similar to those for gene and protein delivery 6, 194 . Unlike nucleic acids, the large-sized and cationic charged Cas9 protein is problematic to form stable nanoparticles via electrostatic interactions. The Cas9 protein and sgRNA can be packaged into virus-like particles or extracellular vesicles through protein engineering. The virus-like particles are usually conducted by directly or indirectly incorporating the Cas9 protein to the structural protein of a viral vector, while the sgRNA is co-packaged into the particles by coexpression or utilizing the interaction between aptamer and ABP 143-145. Virus-like particles have been demonstrated to have efficient genome-editing effects in a variety of primary cells. However, virus-like particles have the potential risk of immune responses and difficulty in further functionalization. Among the nonviral vectors, lipid-based vectors are the most investigated materials for RNP delivery so far. Cas9 protein is a highly cationic and large-sized protein that is recalcitrant to be complexed by the carriers via electrostatic interaction. The pre-assembly of negatively charged RNP with sgRNA or further modification of Cas9 with negatively charged GFP or glutamate tags enables Cas9 to form more stable nanoparticles with cationic materials 56, 59. By virtue of electrostatic interactions, hydrophobic interactions, hydrogen bonding, physical encapsulation and coordinative binding, Cas9 RNP can form particles with a variety of synthetic carriers. The formation of nanoparticles is insufficient to deliver RNP into the cells, because the physicochemical properties of the nanoparticles are critical to efficient endocytosis. Therefore, efficient RNP carriers need to go through a series of screening processes to obtain optimal candidates with suitable RNP binding capacity, internalization, intracellular trafficking and cargo release. A simple and effective solution is using multiple materials to form multifunctional nanoparticles containing RNP, and then coating the nanoparticles with well-established cationic polymers or lipids. The strategies to enhance the serum stability and endosomal escape capability in RNP delivery are common to those in gene and protein delivery. Nonviral vectors take advantages in the diversity and maneuverability of functionalization, improved resistance of RNP to protease and nuclease, and limited immunogenicity, but it is not as effective as electroporation and other physical methods in RNP delivery. The recombinant Cas9 protein introduced in this review are basically fused with NLS peptides to enhance its nuclear entry. Furthermore, peptides fused to Cas9 such as CPPs may enable efficient intracellular RNP delivery by direct membrane penetration or endocytosis. Such CPP-Cas9 fusion proteins are simple and convenient than polymer and lipid nanoparticles, nonetheless, the genome editing efficiency mediated by this vector-free strategy is relatively low, and the naked RNP complexes are hard to resist nuclease and protease degradations during delivery.

Spatiotemporally control of CRISPR/Cas9 function is critical for precise genome editing and reducing the off-target effects. Several strategies have been recently developed to render Cas9 RNP responsive to external stimuli, such as light exposure and ultrasound. The responsive delivery systems such as enzyme-responsive and redox-responsive systems also have the advantages of selective and more efficient genome editing. The responsive systems that have been developed for gene and protein delivery provide various referential platforms for controllable RNP delivery. Another burning issue that must be mentioned is how to improve the efficiency of HDR-driven gene alternations. In general, although some progresses have been made by improving the carrier system 244, 246 or the CRISPR/Cas9 system itself 101, 120, 164, 165, 284, or by inhibiting the expression of p53 99, 167, the frequency of HDR/NHEJ/HMEJ/MMEJ-mediated gene correction and addition is still lower than that of NHEJ/MMEJ-mediated gene knockout. Therefore, it's necessary to develop novel delivery systems to enhance the efficiency of gene alternations.

Nonviral carrier based Cas9 RNP delivery systems have shown great promise in scientific and therapeutic application for the high efficiency, biosecurity and multifunction. The prerequisite for carrier-mediated intracellular delivery is the formation of a stable complex with the cargo molecule. The nucleic acid components in the RNP complex provide abundant negative charges and hydrogen bond binding sites for the carriers. The current nonviral carriers mainly interact and form nanoparticles with RNP complexes via electrostatic interactions or hydrogen bonding, and are further stabilized though the interactions between carriers and RNP complexes, or the interactions between vectors themselves. Multiple interactions including hydrophobic interaction, electrostatic interaction, π-π stacking, hydrogen bonding, host-guest interaction, coordination interaction, cation-π interaction and directly covalent cross-linking have been utilized to maintain the stability of formed nanoparticles. To enhance the binding affinity, Cas9 protein can be engineered with negatively-charged GFP or polyglutamic acid tags. Designing specific supramolecular interactions via protein engineering also contributes to resist competition from other proteins. An alternative strategy is to assemble RNP complexes and carriers into particles first, and then coating them with well-developed cationic polymers or lipids. The outer coating material not only improves the stability, but also helps to promote endocytosis and endosomal escape. It is an effective strategy except for requiring complexed preparation. What kinds of particle properties can promote endocytosis needs further study. Usually, nanoparticles with positive charges and/or ligands that interact with the membrane components are more likely to be internalized into cells. After endocytosis, endosomal escape, intracellular release and nuclear translocation of RNP complexes are essential for the genome editing efficiency. The strategies for endosomal escape have been systemically reviewed 275, 285-287. The strong interactions between cargoes and carriers may result in the disfunction of RNP complexes and the difficulty of intracellular release. Reduction responsive and pH-responsive carriers are benefit for the intracellular release of RNP complexes. The released RNP complexes could entry the nuclear with the help of NLS peptides engineered on Cas9 protein and then induce the genome editing. Up to now, abundant efficient nonviral carriers of nucleic acids and proteins have been developed. The RNP complex as a complex of nucleic acid and protein, the similarities and differences of RNP delivery with nucleic acid and protein delivery need further investigations. Then, more efficient delivery carriers may be rationally designed by taking advantages of the experiences and results in these studies. Due to the explosively development of RNP-based genome editing system and the clinical requirement of gene therapy, future efforts may focus on the rational design of delivery vectors to endow the RNP-delivery systems with stimulus responsiveness and higher efficiency on the premise of safety. We believe that the rapid development of CRISPR/Cas9 system will bring unprecedented opportunities for disease treatment.

## Figures and Tables

**Table 1:** CRISPR-Cas9 system based clinical trials

Intervention/treatment	Target gene	Cells	Disrupt/Correct (Insert)	Condition or disease	Phase	Status	Year	ClinicalTrials.gov identifier
Genetic: Edited T cells Drug: CTX	*PD-1*	T cells	Disrupt	Metastatic non-small cell lung cancer	Phase 1	Active, not recruiting	2016	NCT02793856
Biological: PD-1 knockout T cells Drug: CTX/IL-2	*PD-1*	T Cells	Disrupt	Muscle-invasive bladder cancer	Phase 1	Withdrawn	2016	NCT02863913
Biological: PD-1 knockout T cells Drug: CTX/IL-2	*PD-1*	T Cells	Disrupt	Hormone refractory prostate cancer	-	Withdrawn	2016	NCT02867345
Biological: PD-1 knockout T cells Drug: CTX/IL-2	*PD-1*	T Cells	Disrupt	Metastatic renal cell carcinoma	Phase 1	Withdrawn	2016	NCT02867332
Drug: CTX Other: PD-1 knockout T cells	*PD-1*	T Cells	Disrupt	Metastatic non-small cell lung cancer	Phase 1	Active, not recruiting	2016	NCT02793856
Drug: Fludarabine/ CTX Drug: Interleukin-2	*PD-1*	EBV-CTL cells	Disrupt	Advanced stage Epstein-Barr virus (EBV) associated malignancies	Phase 1/2	Recruiting	2017	NCT03044743
Biological: TALEN Biological: CRISPR/Cas9	*E6*/*E7*	HPV16 and HPV18	Disrupt	HPV-related malignant neoplasm	Phase 1	Unknown	2017	NCT03057912
Genetic: CCR5 gene modification	*CCR5*	CD34^+^ hHSPCs	Disrupt	HIV-1-infection	Not Applicable	Recruiting	2017	NCT03164135
UCART019	*TCR* and *β2M*	CAR-T Cells	Disrupt	B Cell leukemia B Cell lymphoma	Phase 1/2	Recruiting	2017	NCT03166878
PD-1 knockout T cells	*PD-1*	T Cells	Disrupt	Esophageal cancer	Not Applicable	Completed	2017	NCT03081715
Anti-mesothelin CAR-T cells	*PD-1* and *TCR*	CAR-T Cells	Disrupt	Mesothelin positive multiple solid tumors	Phase 1	Recruiting	2018	NCT03545815
Biological: NY-ESO-1 redirected autologous T cells Drug: CTX/Fludarabine	*TCR* and *PD-1*	T cells	Disrupt	Multiple myeloma Melanoma Synovial sarcoma Myxoid/Round cell liposarcoma	Phase 1	Terminated	2018	NCT03399448
CTX001	*BCL11A*	CD34^+^ hHSPCs	Disrupt	β-thalassemia	Phase 1/2	Recruiting	2018	NCT03655678
CTX001	*BCL11A*	CD34^+^ hHSPCs	Disrupt	Sickle cell disease Hematological diseases Hemoglobinopathies	Phase 1/2	Recruiting	2018	NCT03745287
iHSCs treatment group	*HBB*	iHSCs	Correct	Thalassemia	Early Phase 1	Not yet recruiting	2018	NCT03728322
Mesothelin-directed CAR-T cells	*PD-1*	CAR-T cells	Disrupt	Mesothelin positive multiple solid tumors	Phase 1	Recruiting	2018	NCT03747965
Genetic: CD7.CAR/28zeta CAR T cells Drug: Fludarabine/Cytoxan	*CD7*	T cells	Disrupt	T-cell acute lymphoblastic Leukemia T-cell acute lymphoblastic Lymphoma T-non-Hodgkin lymphoma	Phase 1	Not yet recruiting	2018	NCT03690011
Universal Dual Specificity CD19 and CD20 or CD22 CAR-T Cells	Unknown	CAR-T cells	Unknown	B cell leukemia B cell lymphoma	Phase 1/2	Recruiting	2018	NCT03398967
AGN-151587	*CEP290*	Retinal cells	Disrupt	Leber congenital amaurosis 10	Phase 1/2	Recruiting	2019	NCT03872479
Genetic: XYF19 CAR-T cells Drug: CTX/Fludarabine	*HPK1*	CAR-T Cells	Disrupt	Relapsed or refractory CD19^+^ leukemia or lymphoma	Phase 1	Recruiting	2019	NCT04037566
CTX110	*TRAC* and *β2M*	T cells	Disrupt	B-cell malignancy Non-Hodgkin lymphoma B-cell lymphoma	Phase 1/2	Recruiting	2019	NCT04035434
Intervention on primary cultured cells	*KMT2D*	Mesenchymal stem cells	Disrupt	Kabuki syndrome 1	-	Active, not recruiting	2019	NCT03855631
Drug: CTX/Fludarabine/IL-2 TIL	*CISH*	TIL	Disrupt	Gastro-intestinal cancer	Phase 1/2	Recruiting	2020	NCT04426669
CTX120	*TRAC* and *β2M*	T Cells	Disrupt and insert	Relapsed or refractory multiple myeloma	Phase 1	Recruiting	2020	NCT04244656
CTX130	*TCR, MHC I*	T cells	Disrupt and insert	Renal cell carcinoma	Phase 1	Recruiting	2020	NCT04438083
TACE, PD-1 knockout engineered T cells	*PD-1*	T Cells	Disrupt	Advanced hepatocellular carcinoma	Phase 1	Recruiting	2020	NCT04417764

CTX: Cyclophosphamide, TIL: Tumor-infiltrating lymphocytes, HPV: Human papillomavirus, TACE: Transcatheter arterial chemoembolization, IL-2: Interleukin-2,

**Table 2:** Characteristics of CRISPR RNP delivery systems.

Delivery system		Cell Type	Efficiency	Advantages	Disadvantages	Refs
**Physical approaches**	Microinjection	Embryo cells	80% - 100% editing by deep sequencing	● High efficiency ● High specificity	○ Low throughput ○ Require highly skilled manipulation ○ Need specialized equipment	72-83
	Biolistics	Plant cells	~0.7% mutation frequencies by deep sequencing	● High throughput ● Ease of use	○ Low frequency	84-91
	Electroporation	Almost all cell types	24% - 98% indels by T7E1 assay	● High efficiency ● Widely used ● Suitable for most cells	○ May induce cell death ○ Nonspecific ○ Hard to directly use* in vivo*	60, 93-115, 118-120
	Microfluidics	Suspension cells	33% - 47% indels by T7E1 assay 7.8% knock-in efficiency	● High throughput ● Tolerable cell damage ● Rapid delivery ● Suitable for most cells	○ Require specialized equipment to fabricate ○ Susceptible to plugging	121, 124
	Filtroporation	HSCs	44% - 59% indels on by T7E1 assay			126
	Nanotube	MEFs	~14% editing efficiency by flow cytometry	● Minimal cell damage	○ Relatively low efficiency ○ Require specialized equipment to fabricate	127
	iTOP	KBM7 cells hESCs	56.1% editing efficiency by flow cytometry 26.3% editing efficiency by flow cytometry	● Low cost ● Ease of use	○ Not suitable for* in vivo* applications ○ Need two-round transduction	62
	Protoplast transformation	Protoplast cells	20% - 90% indels by T7E1 assay or deep sequencing	● Well developed ● High efficiency ● Low cost	○ Limited cells type ○ Need isolation of protoplasts	85, 132-142
**Virus-like particles**		TZM bl cells Jurkat T cells J-Lat cells HEK293T cells Mouse primary BM cells Human iPSCs B lymphoblastoid cells	16% indels in *CD4* gene by T7E1 assay 14% indels in *CD4* gene by T7E1 assay 28% indels in HIV LTR by T7E1 assay 71% editing efficiency on *EMX1* by T7E1 assay 76% editing efficiency on *Fto* by TIDE analysis 67% editing efficiency on *EMX1* by deep sequencing 18.3% indels in *IL2RG* by deep sequencing	● Reachable to high efficiency ● Effective to difficult-to-transfect cells ● Feasible of *in vivo* application	○ Potential immune response ○ Tedious preparation	143 144 145
**Lipid nanoparticles**	**Extracellular vesicles**	U2OS cells HEK293T cells CHME-5 cells Jurkat T cells Human iPSCs HEK293T cells	13.4% GFP editing efficiency by flow cytometry ~60% editing in *CXCR4* and ~30% editing in *VEGFA* by TIDE analysis 8% editing efficiency in HIV LTR by TIDE analysis 48% indels in *CCR5* by T7E1 assay ~50% indels targeting exon 45 SA and SD site by T7E1 assay 32.5% indels in *VEGFA* by T7E1 assay	● Reachable to high efficiency ● Feasible of *in vivo* application	○ Tedious preparation	149 151 153 154 155
**Lipid nanoparticles**	**Commercial lipids**	U2OS cells HEK293T cells HEK293FT cells U2OS cells Mouse ESCs Human iPSCs N2A cells A549 cells Bright Yellow-2 protoplast cells HEK293 cells Mouse NIH3T3 cells Human ARPE-19 cells Mouse REP cells HEK293T HEK293T HEK293FT Mouse ESCs Human iPSCs P53^-/-^ RPE cells Dog oviductal epithelia cells U2OS cells	60% indels in *EGFP* by T7E1 assay ~8-11% *EGFP* HDR frequencies 38% indels in *CLTA*, 36% indels in *EMX* and 38% indels in *VEGF* by T7E1 assay 51% indels in *HPRT* by T7E1 assay 18% indels in *HPRT* by T7E1 assay 25% indels in *Rosa 26* by T7E1 assay 5% indels in *HPRT* by T7E1 assay 66% indels in *Rosa 26* by T7E1 assay 20% indels in *HPRT* by T7E1 assay ~60% editing efficiency in *pporRFP* reporter by analysis of GFP-positive cells 16.0% indels in *OPA1*, 12.7% indels on *RS1* and 31.4% indels in *VEGFA* by T7E1 assay 82% indels in *Vegfa* by T7E1 assay 57% indels in *VEGFA* by T7E1 assay 22% and 24% indels* in vivo* in *Vegfa* and* Rosa26* in injected area 8% HDR frequency by deep sequencing when inserting HiBiT into *GAPDH* ~16% EGFP HDR frequency by flow cytometry 85% indels by T7E1 assay 75% indels by T7E1 assay 55% indels by T7E1 assay 2% *GFP* HDR frequency by flow cytometry 47% indels in *TP53* by deep sequencing 21.5% indels in *EGFP* by T7E1 assay	● High efficiency ● Well developed ● Serum tolerance ● Feasible of *in vivo* application	○ Low payload capacity	56 160 161 162 163 164 165 166 167 168 169
	**Lipidoids**	HEK cells HEK cells HEK cells HEK cells SNU398 cells Neuo2a cells HeLa cells HEK cells Liver cells Lung cells	70% editing efficiency in *GFP* by flow cytometry 68.6% editing efficiency in *GFP* by flow cytometry 54.7% editing efficiency in *GFP* by flow cytometry 14.4% editing efficiency in *GFP* by flow cytometry ~31% indels on DPP-4 by T7E1 assay ~30% editing efficiency in *FolR1* by flow cytometry ~40% editing efficiency in GFP by flow cytometry 23% indels in *FolR1* by T7E1 assay ~89% editing efficiency in *GFP* by flow cytometry 24.2% editing efficiency in *GFP* by flow cytometry 2.7% editing in liver by T7E1 and TIDE assay 5.3% editing in lung by T7E1 and TIDE assay			170 171 172 173 174 175 176 281
**CPPs**		HEK293T cells HeLa cells L02 cells HeLa cells A549 cells A549 cells EG7 suspension cells Neural progenitor cells J774A.1 cells Mouse pre-adipocytes Mouse primary PECs Jurkat cells NK cells	16% indels in *CCR5* by T7E1 assay 9.7%-29% indels in *ABCC11* by T7E1 assay 15.2% indels in *CCR5* by T7E1 assay 13.4% indels in *CCR5* by T7E1 assay 31.9% indels in *CCR5* by T7E1 assay 28.3% indels in *CCR5* by T7E1 assay 43.9% indels in *KRAS* by T7E1 assay 73% indels in *PD-L1* by T7E1 assay 83% indels in *PD-L2* by T7E1 assay 13% deletion edits by genomic DNA PCR 40.4% indels in *Gfp* by T7E1 assay 14.4% indels in *Gfp* by T7E1 assay 32.8% indels in *Gfp* by T7E1 assay 13% indels in *HPRT* and 12% indels in *DNMT1* by T7E1 assay 18% indels in *HPRT* and 27% indels in *DNMT1* by T7E1 assay	● Reachable to high efficiency ● Safe ● Small particle size ● Serum tolerance ● Feasible of *in vivo* application	○ Need protein engineering ○ Limited functionalization ○ Sensitive to nuclease and protease	177 178 179 182 5 183 184
**Lipopeptides**		HeLa cells A549 cells DF1 cells HEK cells GBM cells 3TZ cells Human OVCAR8 cells	38% indels in *HPRT1* by T7E1 assay 21% indels in *HPRT1* by T7E1 assay 39% indels in *HPRT1* by T7E1 assay 27.6% indels in *EGFP* by T7E1 assay 19.7% indels in *EGFP* by T7E1 assay 12% editing efficiency by flow cytometry ~12% editing efficiency by flow cytometry	● No need of protein engineering	○ Medium efficiency	188 189 191
**Polymers**	**Dendrimers**	HEK293T cells HCT-116 cells HT-29 cells SH-SY5Y Cells	39.7% indels in *EGFP* by T7E1 assay 23.1% indels in *AAVS1* by T7E1 assay 21.1% indels in *HBB* by T7E1 assay 29.6% indels in *AAVS1* by T7E1 assay 22.5% indels in *HBB* by T7E1 assay 23.9% indels in *AAVS1* by T7E1 assay 14.9.5% indels in *HBB* by T7E1 assay ~6% indels in *AAVS1* by TIDE analysis ~5% indels in *Rosa26* around injection site of brain by TIDE analysis	● Low cost ● Ease of use ● Ease of functionalization ●Safe ● Reachable to high efficiency	○ Relatively low efficiency	57 209
	**PBAEs**	HEK293T cells GL261 cells	77% editing efficiency in *GFP* by flow cytometry 4% HDR frequency by quantifying HindIII cleavage 47% editing efficiency in *GFP* by flow cytometry			212
	**PLL**	U87MG cells	39.1% indels in *STAT3* by T7E1 assay 32.1% editing efficiency in *EGFP* by flow cytometry Down-regulated expression of STAT3 by 48% and RUNX1 by 50% in the heterogeneous tumors via Western blot			218
	**CS nanoparticles**	HEK293T cells RAW264.7 cells HeLa cells U2OS cells A549 cells HEK293 cells	46.7% indels in *RRDX4* by T7E1 assay 24.4% indels in *RRDX4* by T7E1 assay 32.6% indels in *RRDX4* by T7E1 assay 55.8% indels in *RRDX4* by T7E1 assay 16.9% indels in *RRDX4* by T7E1 assay 12.5% *GFP* HDR frequency by flow cytometry			219
	**Supramolecular polymers**	SW-480 cells	Induced a total apoptotic rate of 48.0% by editing *KRAS*			220
	**Reduction-sensitive polymers**	HEK293 cells	~50% editing efficiency in *mCherry* and *GFP* by flow cytometry			221, 222
**Nanogels**		HEK293 cells HeLa cells	~80% editing efficiency in *mCherry* by flow cytometry ~30% indels in *APP* by deep sequencing 18.7% indels in EGFP by T7E1 assay	● Serum tolerance ● Safe ● High loading capacity ● Feasible of *in vivo* application	○ Relatively low efficiency	223 226
**Inorganic nanoparticles**	**GNPs**	HeLa cells RAW264.7 cells A375 cells HeLa cells Human ESCs Human iPSCs Mouse primary myoblasts Muscle fibrosis Brain striatum cells HSPCs	29% indels in *AAVS1* by T7E1 assay 30% indels in *PTEN* by T7E1 assay 27% indels in *SIRP-α* by T7E1 assay 26.2% indels in *Plk1* by T7E1 assay 34% indels in *E6* by T7E1 assay ~3.5% *CXCR4* HDR frequency by quantifying HindIII cleavage ~3.5% *CXCR4* HDR frequency by quantifying HindIII cleavage 3.3% HDR frequency in dystrophin gene 5.4% HDR frequency in dystrophin gene by deep sequencing 14.6% indels in *mGluR5* around injection site by TIDE analysis 13.4% *CCR5* HDR frequency and 8.8% HDR at γ-globin promoter locus by TIDE analysis	● Ease of preparation ● Ease of use ● Ease of functionalization ● Safe	○ Potential toxicity *in vivo*	59 230 231, 232 233 244 245 246
	**MOFs**	CHO cells MCF-7 cells HeLa cells HEK293 cells	30% indels in *EGFP* by T7E1 assay ~60 editing in *EGFP* by flow cytometry ~40% editing in *EGFP* by flow cytometry Over 40% editing in *GFP* by flow cytometry Over 20% *GFP* HDR frequency by flow cytometry	● Ease of preparation ● Low cost ● Fast endosomal escape	○ Potential toxicity of metal ions	249 250 251 252
	**GO**	AGS cells	~39% editing in EGFP by flow cytometry ~33% reduction of *CXCR-4* mRNA by RT-PCR analysis	● High payload capacity ● Ease of functionalization	○ Relatively low efficiency	256
	**BP nanosheets**	MCF-7 cells hBMSCs RAW264.7 cells A549 cells	32.1% indels in *GRIN2B* by T7E1 assay 22.8% indels in *GRIN2B* by T7E1 assay 17.2% indels in *Grin2b* by T7E1 assay 23.7% indels in *EGFP* by T7E1 assay	● Excellent biocompatibility ● Biodegradable ● High payload capacity	○ Relatively low efficiency ○ Rapid degradation	260
	**Calcium phosphate**	Protoplast cells	20% indels in *SDH* by T7E1 assay	● Stable	○ Low efficiency	263
**DNA nanoclews**		U2OS cells 3T3-L1 cells U2OS cells U2OS cells HeLa cells	28% indels in *EGFP* by T7E1 assay ~75% indels in *Pcsk9* by T7E1 assay ~78% indels in *EGFP* by T7E1 assay 63.9% indels in *GFP* by T7E1 assay	● Controllable size and architecture ● High efficiency	○ Poor stability of DNA carrier	266 267 268
**Light-responsive materials**		HEK293T cells Embryo cells A549 cells	26.2% indels in *CTNNb1* by TIDE analysis 69.4% indels in *EGFP* by TIDE analysis 32.2% indels in *PLK*-1 by T7E1 assay	● Precise spatiotemporal control ● Reduce the off-target effect	○ Non-repeatable control	269 272
**US-responsive materials**		Human DPCs	67.1% indels in *SRD5A2* by T7E1 assay			273
**Targeted delivery systems**	**Galactose based targeting**	HepG2 cells HepG2 cells Hepa 1-6 cells 3T3-L1 cells U2OS cells	4.8% indels in *EMX1* by deep sequencing 9% - 16% indels in *EMX1* by T7E1 assay 57% indels in *Psck9* by T7E1 assay ~75% indels in *Pcsk9* by T7E1 assay ~78% indels in *EGFP* by T7E1 assay	● Specific delivery ● Reduce the off-target effect	○ Fewer targeting types	276 277 278 267
	**Folate based targeting**	HeLa cells	47.1% indels in *PLK1* by T7E1 assay			280
	**HA based targeting**	SW-480 cells	Induced a total apoptotic rate of 48.0% by editing *KRAS*			220
	**RGD based targeting**	3TZ cells Human OVCAR8 cells U87 cells GS5 cells	12% editing efficiency by flow cytometry ~12% editing efficiency by flow cytometry Inhibition of cell proliferation by 52.8% via editing *PLK1* Inhibition of cell proliferation by 58.3% via editing *PLK1*			191 279
	**Cell membrane-based targeting**	MCF-7 cells	~60 editing in *EGFP* by flow cytometry			250
	**ARTA based targeting**	HEK293 cells HEK293 cells	Over 40% editing in *GFP* by flow cytometry Over 20% *GFP* HDR frequency by flow cytometry ~80% editing efficiency in *mCherry* by flow cytometry ~30% indels in *APP* by deep sequencing			252 223
	**SORT based targeting**	Liver cells Lung cells	2.7% editing in liver by T7E1 and TIDE assay 5.3% editing in lung by T7E1 and TIDE assay			281

## Abbreviations

CRISPR: clustered regularly interspaced short palindromic repeat
RNP: ribonucleoprotein
sgRNA: single guide RNA
CASCADE: CRISPR-associated complex for antiviral defense
PAM: protospacer adjacent motif
DSB: double-stranded break
NHEJ: nonhomologous end joining
HDR: homology directed repair
pDNA: plasmid DNA
mRNA: messenger RNA
DmYP1: *D. melanogaster* Yolk Protein 1
HSPCs: hemopoietic stem/progenitor cells
HSCs: hemopoietic stem cells
hESCs: human embryonic stem cells
iPSCs: human induced pluripotent stem cells
HEK: human embryonic kidney
PDMS: polydimethylsiloxane
tCTSs: truncated Cas9 target sequences
MAPKs: mitogen-activated protein kinases
MFEs: mouse embryonic fibroblast cells
PEG: Polyethylene glycol
iTOP: induced transduction by osmocytosis and propanebetaine
LV: lentivirus
LTR: long terminal repeat
HIV: human immunodeficiency virus
ABP: aptamer-binding protein
TIDE: Tracking Indels by Decomposition
SELEX: systemic evolution of ligands by exponential enrichment
EVs: extracellular vesicles
PECs: peritoneal exudate cells
ARRDC1: arrestin domain containing protein 1
DOPE: 1,2-dioleoyl-*sn*-glycero-3-phosphoethanolamine
NTA: nitrilotriacetic acid
OAAs: oligo(ethylenamino) amides
CPPs: cell penetrant peptides
SCP: supercharged peptide
MMP-2: matrix metalloproteinase 2
NLS: nuclear location sequence
LMWP: low-molecular-weight protamine
PEI: polyethyleneimine
SV40: Simian vacuolating virus 40
PBAEs: poly(β-amino ester)s
PLL: polylysine
PBA: phenylboronic acid
PAMAM: polyamidoamine
CS: chitosan
RFP: red fluorescence protein
RPE: retinal pigment epithelial
GNPs: gold nanoparticles
SIRP-α: signal regulatory protein-α
DOTAP: 1,2-dioleoyl-3-trimethylammoniumpropane
DSPE: 1,2-distearroyl-*sn*-glycero-3-phosphoethanolamine
Plk1: polo-like kinase 1
SNAs: spherical nucleic acids
PAsp(DET): polymer poly(N-(N-(2-aminoethyl)-2-aminoethyl) aspartamide)
OEG: oligo(ethylene glycol)
MOFs: metal-organic frameworks
ZIFs: zeolitic imidazolate frameworks
CHO: Chinese hamster ovary
GO: graphene oxide
2D: two-dimensional
BP: black phosphorus
hBMSCs: human bone marrow derived mesenchymal stem cells
NPOM: 6-Nitropiperonyloxymethylene
UV: ultraviolet
UCNPs: upconversion nanoparticles
DPPE: 1,2-dipalmitoyl-*sn*-glycero-3-phosphoethanolamine
DPCs: dermal papilla cells
AD: adamantane
β-CD: β-cyclodextrin
Gal: galactose
ASGPr: asialoglycoprotein receptor
DM: 2,3-dimethylmaleic anhydride
PCSK9: proprotein convertase subtilisin/kexin type 9
LDLR: low-density lipoprotein receptor
HA: hyaluronic acid
ATRA: All-*trans* retinoic acid
IRBP: inter-photoreceptor retinoid-binding protein
SORT: selective organ targeting

## References

[1] Doudna JA, Charpentier E (2014). The new frontier of genome engineering with CRISPR-Cas9. Science.

[2] Wilson RC, Gilbert LA (2018). The promise and challenge of in vivo delivery for genome therapeutics. ACS Chem Biol.

[3] Cox DBT, Platt RJ, Zhang F (2015). Therapeutic genome editing: prospects and challenges. Nat Med.

[4] Eid A, Mahfouz MM (2016). Genome editing: the road of CRISPR/Cas9 from bench to clinic. Exp Mol Med.

[5] Staahl BT, Benekareddy M, Coulon-Bainier C, Banfal AA, Floor SN, Sabo JK (2017). Efficient genome editing in the mouse brain by local delivery of engineered Cas9 ribonucleoprotein complexes. Nat Biotechnol.

[6] Mintzer MA, Simanek EE (2009). Nonviral vectors for gene delivery. Chem Rev.

[7] Hussain W, Mahmood T, Hussain J, Ali N, Shah T, Qayyum S (2019). CRISPR/Cas system: A game changing genome editing technology, to treat human genetic diseases. Gene.

[8] Khadempar S, Familghadakchi S, Motlagh RA, Farahani N, Dashtiahangar M, Rezaei H (2019). CRISPR-Cas9 in genome editing: Its function and medical applications. J Cell Physiol.

[9] Ishino Y, Shinagawa H, Makino K, Amemura M, Nakata A (1987). Nucleotide sequence of the iap gene, responsible for alkaline phosphatase isozyme conversion in Escherichia coli, and identification of the gene product. J Bacteriol.

[10] Jansen R, Embden JDAv, Gaastra W, Schouls LM (2002). Identification of genes that are associated with DNA repeats in prokaryotes. Mol Microbiol.

[11] Mojica FJM, Díez-Villaseñor Cs, García-Martínez J, Soria E (2005). Intervening sequences of regularly spaced prokaryotic repeats derive from foreign genetic elements. J Mol Evol.

[12] Bolotin A, Quinquis B, Sorokin A, Ehrlich SD (2005). Clustered regularly interspaced short palindrome repeats (CRISPRs) have spacers of extrachromosomal origin. Microbiology.

[13] Barrangou R, Fremaux C, Deveau H, Richards M, Boyaval P, Moineau S (2007). CRISPR provides acquired resistance against viruses in prokaryotes. Science.

[14] Jinek M, Chylinski K, Fonfara I, Hauer M, Doudna JA, Charpentier E (2012). A programmable dual-RNA-guided DNA endonuclease in adaptive bacterial immunity. Science.

[15] Cong L, Ran FA, Cox D, Lin S, Barretto R, Habib N (2013). Multiplex genome engineering using CRISPR/Cas systems. Science.

[16] Mali P, Yang L, Esvelt KM, Aach J, Guell M, DiCarlo JE (2013). RNA-guided human genome engineering via Cas9. Science.

[17] Hwang WY, Fu Y, Reyon D, Maeder ML, Tsai SQ, Sander JD (2013). Efficient genome editing in zebrafish using a CRISPR-Cas system. Nat Biotechnol.

[18] Wang H, Yang H, Shivalila CS, Dawlaty MM, Cheng AW, Zhang F (2013). One-step generation of mice carrying mutations in multiple genes by CRISPR/Cas-mediated genome engineering. Cell.

[19] Niu Y, Shen B, Cui Y, Chen Y, Wang J, Wang L (2014). Generation of gene-modified cynomolgus monkey via Cas9/RNA-mediated gene targeting in one-cell embryos. Cell.

[20] Bassett AR, Liu JL (2014). CRISPR/Cas9 and genome editing in Drosophila. J Genet Genomics.

[21] Oh JH, van Pijkeren JP (2014). CRISPR-Cas9-assisted recombineering in Lactobacillus reuteri. Nucleic Acids Res.

[22] Belhaj K, Chaparro-Garcia A, Kamoun S, Patron NJ, Nekrasov V (2015). Editing plant genomes with CRISPR/Cas9. Curr Opin Biotechnol.

[23] Li JF, Zhang D, Sheen J (2014). Cas9-based genome editing in Arabidopsis and tobacco. Methods Enzymol.

[24] Li D, Qiu Z, Shao Y, Chen Y, Guan Y, Liu M (2013). Heritable gene targeting in the mouse and rat using a CRISPR-Cas system. Nat Biotechnol.

[25] Makarova KS, Wolf YI, Alkhnbashi OS, Costa F, Shah SA, Saunders SJ (2015). An updated evolutionary classification of CRISPR-Cas systems. Nat Rev Microbiol.

[26] Mashimo T (2014). Gene targeting technologies in rats: Zinc finger nucleases, transcription activator-like effector nucleases, and clustered regularly interspaced short palindromic repeats. Dev, Growth Differ.

[27] Sander JD, Joung JK (2014). CRISPR-Cas systems for editing, regulating and targeting genomes. Nat Biotechnol.

[28] Liu C, Zhang L, Liu H, Cheng K (2017). Delivery strategies of the CRISPR-Cas9 gene-editing system for therapeutic applications. J Controlled Release.

[29] van der Oost J, Westra ER, Jackson RN, Wiedenheft B (2014). Unravelling the structural and mechanistic basis of CRISPR-Cas systems. Nat Rev Microbiol.

[30] Garneau JE, Dupuis M, Villion M, Romero DA, Barrangou R, Boyaval P (2010). The CRISPR/Cas bacterial immune system cleaves bacteriophage and plasmid DNA. Nature.

[31] Jinek M, Chylinski K, Fonfara I, Hauer M, Doudna JA, Charpentier E (2012). A programmable dual-RNA-guided DNA endonuclease in adaptive bacterial immunity. Science.

[32] Jinek M, Jiang F, Taylor DW, Sternberg SH, Kaya E, Ma E (2014). Structures of Cas9 endonucleases reveal RNA-mediated conformational activation. Science.

[33] Liang F, Han M, Romanienko PJ, Jasin M (1998). Homology-directed repair is a major double-strand break repair pathway in mammalian cells. Proc Natl Acad Sci U S A.

[34] Moehle EA, Rock JM, Lee Y-L, Jouvenot Y, DeKelver RC, Gregory PD (2007). Targeted gene addition into a specified location in the human genome using designed zinc finger nucleases. Proc Natl Acad Sci U S A.

[35] Li L, Hu S, Chen X (2018). Non-viral delivery systems for CRISPR/Cas9-based genome editing: Challenges and opportunities. Biomaterials.

[36] Wilson JH, Berget PB, Pipas JM (1982). Somatic cells efficiently join unrelated DNA segments end-to-end. Mol Cell Biol.

[37] Roth DB, Wilson JH (1985). Relative rates of homologous and nonhomologous recombination in transfected DNA. Proc Natl Acad Sci U S A.

[38] Capecchi MR (1989). Altering the genome by homologous recombination. Science.

[39] Jensen RB, Rothenberg E (2020). Preserving genome integrity in human cells via DNA double-strand break repair. Mol Biol Cell.

[40] Thomas KR, Folger KR, Capecchi MR (1986). High frequency targeting of genes to specific sites in the mammalian genome. Cell.

[41] Smithies O, Gregg RG, Boggs SS, Koralewski MA, Kucherlapati RS (1985). Insertion of DNA sequences into the human chromosomal β-globin locus by homologous recombination. Nature.

[42] Suzuki K, Izpisua Belmonte JC (2018). In vivo genome editing via the HITI method as a tool for gene therapy. J Hum Genet.

[43] Cui Z, Jiang X, Zheng H, Qi Q, Hou J (2019). Homology-independent genome integration enables rapid library construction for enzyme expression and pathway optimization in Yarrowia lipolytica. Biotechnol Bioeng.

[44] Gao D, Smith S, Spagnuolo M, Rodriguez G, Blenner M (2018). Dual CRISPR-Cas9 cleavage mediated gene excision and targeted integration in Yarrowia lipolytica. Biotechnol J.

[45] Seol JH, Shim EY, Lee SE (2018). Microhomology-mediated end joining: Good, bad and ugly. Mutat Res.

[46] van Schendel R, van Heteren J, Welten R, Tijsterman M (2016). Genomic scars generated by polymerase theta reveal the versatile mechanism of alternative end-joining. PLoS Genet.

[47] Li L, Liu X, Wei K, Lu Y, Jiang W (2019). Synthetic biology approaches for chromosomal integration of genes and pathways in industrial microbial systems. Biotechnol Adv.

[48] Nakade S, Tsubota T, Sakane Y, Kume S, Sakamoto N, Obara M (2014). Microhomology-mediated end-joining-dependent integration of donor DNA in cells and animals using TALENs and CRISPR/Cas9. Nat Commun.

[49] Auer TO, Duroure K, De Cian A, Concordet JP, Del Bene F (2014). Highly efficient CRISPR/Cas9-mediated knock-in in zebrafish by homology-independent DNA repair. Genome Res.

[50] Hisano Y, Sakuma T, Nakade S, Ohga R, Ota S, Okamoto H (2015). Precise in-frame integration of exogenous DNA mediated by CRISPR/Cas9 system in zebrafish. Sci Rep.

[51] Suzuki K, Tsunekawa Y, Hernandez-Benitez R, Wu J, Zhu J, Kim EJ (2016). In vivo genome editing via CRISPR/Cas9 mediated homology-independent targeted integration. Nature.

[52] Yao X, Wang X, Hu X, Liu Z, Liu J, Zhou H (2017). Homology-mediated end joining-based targeted integration using CRISPR/Cas9. Cell Res.

[53] Yao X, Wang X, Liu J, Shi L, Huang P, Yang H CRISPR/Cas9-mediated targeted integration in vivo using a homology-mediated end joining-based strategy. J Vis Exp. 2018: 56844.

[54] Xie L, Sun J, Mo L, Xu T, Shahzad Q, Chen D (2019). HMEJ-mediated efficient site-specific gene integration in chicken cells. J Biol Eng.

[55] Wang M, Glass ZA, Xu Q (2017). Non-viral delivery of genome-editing nucleases for gene therapy. Gene Ther.

[56] Zuris JA, Thompson DB, Shu Y, Guilinger JP, Bessen JL, Hu JH (2015). Cationic lipid-mediated delivery of proteins enables efficient protein-based genome editing *in vitro* and *in vivo*. Nat Biotechnol.

[57] Liu C, Wan T, Wang H, Zhang S, Ping Y, Cheng Y (2019). A boronic acid-rich dendrimer with robust and unprecedented efficiency for cytosolic protein delivery and CRISPR-Cas9 gene editing. Sci Adv.

[58] Guan X, Luo Z, Sun W (2018). A peptide delivery system sneaks CRISPR into cells. J Biol Chem.

[59] Mout R, Ray M, Yesilbag Tonga G, Lee YW, Tay T, Sasaki K (2017). Direct cytosolic delivery of CRISPR/Cas9-ribonucleoprotein for efficient gene editing. ACS Nano.

[60] Lattanzi A, Meneghini V, Pavani G, Amor F, Ramadier S, Felix T (2019). Optimization of CRISPR/Cas9 delivery to human hematopoietic stem and progenitor cells for therapeutic genomic rearrangements. Mol Ther.

[61] Chandrasekaran AP, Song M, Kim KS, Ramakrishna S (2018). Different methods of delivering CRISPR/Cas9 into cells. Prog Mol Biol Transl Sci.

[62] D'Astolfo DS, Pagliero RJ, Pras A, Karthaus WR, Clevers H, Prasad V (2015). Efficient intracellular delivery of native proteins. Cell.

[63] Chiper M, Niederreither K, Zuber G (2018). Transduction methods for cytosolic delivery of proteins and bioconjugates into living cells. Adv Healthc Mater.

[64] Lostalé-Seijo I, Montenegro J (2018). Synthetic materials at the forefront of gene delivery. Nat Rev Chem.

[65] Lv J, He B, Yu J, Wang Y, Wang C, Zhang S (2018). Fluoropolymers for intracellular and in vivo protein delivery. Biomaterials.

[66] Qin X, Yu C, Wei J, Li L, Zhang C, Wu Q (2019). Rational design of nanocarriers for intracellular protein delivery. Adv Mater.

[67] Stewart MP, Langer R, Jensen KF (2018). Intracellular delivery by membrane disruption: Mechanisms, strategies, and concepts. Chem Rev.

[68] Lee YW, Luther DC, Kretzmann JA, Burden A, Jeon T, Zhai S (2019). Protein delivery into the cell cytosol using non-viral nanocarriers. Theranostics.

[69] Geng WC, Huang Q, Xu Z, Wang R, Guo DS (2019). Gene delivery based on macrocyclic amphiphiles. Theranostics.

[70] Yin H, Kanasty RL, Eltoukhy AA, Vegas AJ, Dorkin JR, Anderson DG (2014). Non-viral vectors for gene-based therapy. Nat Rev Genet.

[71] Wang HX, Li M, Lee CM, Chakraborty S, Kim HW, Bao G (2017). CRISPR/Cas9-based genome editing for disease modeling and therapy: Challenges and opportunities for nonviral delivery. Chem Rev.

[72] Gagnon JA, Valen E, Thyme SB, Huang P, Ahkmetova L, Pauli A (2014). Efficient Mutagenesis by Cas9 Protein-Mediated Oligonucleotide Insertion and Large-Scale Assessment of Single-Guide RNAs. PLoS One.

[73] Kotani H, Taimatsu K, Ohga R, Ota S, Kawahara A (2015). Efficient Multiple Genome Modifications Induced by the crRNAs, tracrRNA and Cas9 Protein Complex in Zebrafish. PLoS One.

[74] Liu P, Luk K, Shin M, Idrizi F, Kwok S, Roscoe B (2019). Enhanced Cas12a editing in mammalian cells and zebrafish. Nucleic Acids Res.

[75] Kim K, Ryu SM, Kim ST, Baek G, Kim D, Lim K (2017). Highly efficient RNA-guided base editing in mouse embryos. Nat Biotechnol.

[76] Song Y, Xu Y, Liang M, Zhang Y, Chen M, Deng J (2018). CRISPR/Cas9-mediated mosaic mutation of SRY gene induces hermaphroditism in rabbits. Biosci Rep.

[77] Fei J-F, Schuez M, Knapp D, Taniguchi Y, Drechsel DN, Tanaka EM (2017). Efficient gene knockin in axolotl and its use to test the role of satellite cells in limb regeneration. Proc Natl Acad Sci U S A.

[78] Cleves PA, Strader ME, Bay LK, Pringle JR, Matz MV (2018). CRISPR/Cas9-mediated genome editing in a reef-building coral. Proc Natl Acad Sci U S A.

[79] Dermauw W, Jonckheere W, Riga M, Livadaras I, Vontas J, Van Leeuwen T (2020). Targeted mutagenesis using CRISPR-Cas9 in the chelicerate herbivore Tetranychus urticae. Insect Biochem Mol Biol.

[80] Meccariello A, Tsoumani KT, Gravina A, Primo P, Buonanno M, Mathiopoulos KD (2020). Targeted somatic mutagenesis through CRISPR/Cas9 ribonucleoprotein complexes in the olive fruit fly, *Bactrocera oleae*. Arch Insect Biochem Physiol.

[81] Chaverra-Rodriguez D, Macias VM, Hughes GL, Pujhari S, Suzuki Y, Peterson DR (2018). Targeted delivery of CRISPR-Cas9 ribonucleoprotein into arthropod ovaries for heritable germline gene editing. Nat Commun.

[82] Macias VM, McKeand S, Chaverra-Rodriguez D, Hughes GL, Fazekas A, Pujhari S (2020). Cas9-mediated gene-editing in the malaria mosquito *Anopheles stephensi* by ReMOT Control. G3 (Bethesda).

[83] Heu CC, McCullough FM, Luan JB, Rasgon JL (2020). CRISPR-Cas9-based genome editing in the silverleaf whitefly (*Bemisia tabaci*). Crispr Journal.

[84] Svitashev S, Schwartz C, Lenderts B, Young JK, Mark Cigan A (2016). Genome editing in maize directed by CRISPR-Cas9 ribonucleoprotein complexes. Nat Commun.

[85] Liang Z, Chen K, Li T, Zhang Y, Wang Y, Zhao Q (2017). Efficient DNA-free genome editing of bread wheat using CRISPR/Cas9 ribonucleoprotein complexes. Nat Commun.

[86] Liang Z, Chen K, Gao C (2019). Biolistic delivery of CRISPR/Cas9 with ribonucleoprotein complex in wheat. Methods Mol Biol.

[87] Banakar R, Eggenberger AL, Lee K, Wright DA, Murugan K, Zarecor S (2019). High-frequency random DNA insertions upon co-delivery of CRISPR-Cas9 ribonucleoprotein and selectable marker plasmid in rice. Sci Rep.

[88] Serif M, Dubois G, Finoux AL, Teste MA, Jallet D, Daboussi F (2018). One-step generation of multiple gene knock-outs in the diatom Phaeodactylum tricornutum by DNA-free genome editing. Nat Commun.

[89] Makhotenko AV, Khromov AV, Snigir EA, Makarova SS, Makarov VV, Suprunova TP (2019). Functional analysis of coilin in virus resistance and stress tolerance of potato solanum tuberosum using CRISPR-Cas9 editing. Dokl Biochem Biophys.

[90] Wang P (2018). Two distinct approaches for CRISPR-Cas9-mediated gene editing in cryptococcus neoformans and related species. Msphere.

[91] Chang KS, Kim J, Park H, Hong S-J, Lee C-G, Jin E (2020). Enhanced lipid productivity in AGP knockout marine microalga Tetraselmis sp. using a DNA-free CRISPR-Cas9 RNP method. Bioresour Technol.

[92] Boukany PE, Morss A, Liao WC, Henslee B, Jung H, Zhang X (2011). Nanochannel electroporation delivers precise amounts of biomolecules into living cells. Nat Nanotechnol.

[93] Modarai SR, Man D, Bialk P, Rivera-Torres N, Bloh K, Kmiec EB (2018). Efficient delivery and nuclear uptake is not sufficient to detect gene editing in CD34+ cells directed by a ribonucleoprotein complex. Mol Ther - Nucleic Acids.

[94] Hendel A, Bak RO, Clark JT, Kennedy AB, Ryan DE, Roy S (2015). Chemically modified guide RNAs enhance CRISPR-Cas genome editing in human primary cells. Nat Biotechnol.

[95] Dever DP, Bak RO, Reinisch A, Camarena J, Washington G, Nicolas CE (2016). CRISPR/Cas9 β-globin gene targeting in human haematopoietic stem cells. Nature.

[96] Wang L, Li L, Ma Y, Hu H, Li Q, Yang Y (2020). Reactivation of γ-globin expression through Cas9 or base editor to treat β-hemoglobinopathies. Cell Research.

[97] Wu Y, Zeng J, Roscoe BP, Liu P, Yao Q, Lazzarotto CR (2019). Highly efficient therapeutic gene editing of human hematopoietic stem cells. Nat Med.

[98] Vakulskas CA, Dever DP, Rettig GR, Turk R, Jacobi AM, Collingwood MA (2018). A high-fidelity Cas9 mutant delivered as a ribonucleoprotein complex enables efficient gene editing in human hematopoietic stem and progenitor cells. Nat Med.

[99] Ihry RJ, Worringer KA, Salick MR, Frias E, Ho D, Theriault K (2018). p53 inhibits CRISPR-Cas9 engineering in human pluripotent stem cells. Nat Med.

[100] Gundry MC, Brunetti L, Lin A, Mayle AE, Kitano A, Wagner D (2016). Highly efficient genome editing of murine and human hematopoietic progenitor cells by CRISPR/Cas9. Cell Rep.

[101] Lin S, Staahl BT, Alla RK, Doudna JA (2014). Enhanced homology-directed human genome engineering by controlled timing of CRISPR/Cas9 delivery. eLife.

[102] Ma L, Jang L, Chen J, Song J, Yang D, Zhang J CRISPR/Cas9 ribonucleoprotein-mediated precise gene editing by tube electroporation. J Vis Exp. 2019: e59512.

[103] Hung KL, Meitlis I, Hale M, Chen C-Y, Singh S, Jackson SW (2018). Engineering protein-secreting plasma cells by homology-directed repair in primary human B cells. Mol Ther.

[104] Schumann K, Lin S, Boyer E, Simeonov DR, Subramaniam M, Gate RE (2015). Generation of knock-in primary human T cells using Cas9 ribonucleoproteins. Proc Natl Acad Sci U S A.

[105] Kagoya Y, Guo T, Yeung B, Saso K, Anczurowski M, Wang CH (2020). Genetic ablation of HLA class I, class II, and the T cell receptor enables allogeneic T cells to be used for adoptive T cell therapy. Cancer Immunol Res.

[106] Leonetti MD, Sekine S, Kamiyama D, Weissman JS, Huang B (2016). A scalable strategy for high-throughput GFP tagging of endogenous human proteins. Proc Natl Acad Sci U S A.

[107] Lee JK, Jeong E, Lee J, Jung M, Shin E, Kim Yh (2018). Directed evolution of CRISPR-Cas9 to increase its specificity. Nat Commun.

[108] Nussing S, House IG, Kearney CJ, Chen AXY, Vervoort SJ, Beavis PA (2020). Efficient CRISPR/Cas9 gene editing in uncultured naive mouse T cells for in vivo studies. J Immunol.

[109] Kalebic N, Taverna E, Tavano S, Wong FK, Suchold D, Winkler S (2016). CRISPR/Cas9-induced disruption of gene expression in mouse embryonic brain and single neural stem cells in vivo. EMBO reports.

[110] Wu W, Lu Z, Li F, Wang W, Qian N, Duan J (2017). Efficient in vivo gene editing using ribonucleoproteins in skin stem cells of recessive dystrophic epidermolysis bullosa mouse model. Proc Natl Acad Sci U S A.

[111] Modzelewski AJ, Chen S, Willis BJ, Lloyd KCK, Wood JA, He L (2018). Efficient mouse genome engineering by CRISPR-EZ technology. Nat Protoc.

[112] Riggan L, Hildreth AD, Rolot M, Wong YY, Satyadi W, Sun R (2020). CRISPR-Cas9 ribonucleoprotein-mediated genomic editing in mature primary innate immune cells. Cell Rep.

[113] Picariello T, Hou Y, Kubo T, McNeill NA, Yanagisawa H-a, Oda T (2020). TIM, a targeted insertional mutagenesis method utilizing CRISPR/Cas9 in *Chlamydomonas reinhardtii*. PLoS One.

[114] Kim J, Lee S, Baek K, Jin E (2020). Site-specific gene knock-out and on-site heterologous gene overexpression in *Chlamydomonas reinhardtii* via a CRISPR-Cas9-mediated knock-in method. Front Plant Sci.

[115] Malvezzi AM, Aricó M, Souza-Melo N, dos Santos GP, Bittencourt-Cunha P, Holetz FB (2020). GCN2-like kinase modulates stress granule formation during nutritional stress in *Trypanosoma cruzi*. Front Cell Infect Microbiol.

[116] Canatella PJ, Karr JF, Petros JA, Prausnitz MR (2001). Quantitative study of electroporation-mediated molecular uptake and cell viability. Biophysical journal.

[117] Bak RO, Dever DP, Reinisch A, Hernandez DC, Majeti R, Porteus MH (2017). Multiplexed genetic engineering of human hematopoietic stem and progenitor cells using CRISPR/Cas9 and AAV6. eLife.

[118] Yang R, Lemaître V, Huang C, Haddadi A, McNaughton R, Espinosa HD (2018). Monoclonal cell line generation and CRISPR/Cas9 manipulation via single-cell electroporation. Small.

[119] Cao Y, Ma E, Cestellos-Blanco S, Zhang B, Qiu R, Su Y (2019). Nontoxic nanopore electroporation for effective intracellular delivery of biological macromolecules. Proc Natl Acad Sci U S A.

[120] Nguyen DN, Roth TL, Li PJ, Chen PA, Apathy R, Mamedov MR (2020). Polymer-stabilized Cas9 nanoparticles and modified repair templates increase genome editing efficiency. Nat Biotechnol.

[121] Ma Y, Han X, Quintana Bustamante O, Bessa de Castro R, Zhang K, Zhang P (2017). Highly efficient genome editing of human hematopoietic stem cells via a nano-silicon-blade delivery approach. Integr Biol.

[122] Sharei A, Zoldan J, Adamo A, Sim WY, Cho N, Jackson E (2013). A vector-free microfluidic platform for intracellular delivery. Proc Natl Acad Sci U S A.

[123] Fajrial AK, He QQ, Wirusanti NI, Slansky JE, Ding X (2020). A review of emerging physical transfection methods for CRISPR/Cas9-mediated gene editing. Theranostics.

[124] Han X, Liu Z, Ma Y, Zhang K, Qin L (2017). Cas9 ribonucleoprotein delivery via microfluidic cell-deformation chip for human T-cell genome editing and immunotherapy. Adv Biosys.

[125] Williams AR, Bao S, Miller DL (1999). Filtroporation: A simple, reliable technique for transfection and macromolecular loading of cells in suspension. Biotechnol Bioeng.

[126] Yen J, Fiorino M, Liu Y, Paula S, Clarkson S, Quinn L (2018). TRIAMF: A New method for delivery of Cas9 ribonucleoprotein complex to human hematopoietic stem cells. Sci Rep.

[127] Chen Y, Aslanoglou S, Murayama T, Gervinskas G, Fitzgerald LI, Sriram S (2020). Silicon-nanotube-mediated intracellular delivery enables ex vivo gene editing. Adv Mater.

[128] Liu YC, Vidali L Efficient polyethylene glycol (PEG) mediated transformation of the moss *Physcomitrella patens*. J Vis Exp. 2011: e2560.

[129] Berg M, Maruthachalam K, *Genetic Transformation Systems in Fungi, Volume 1*. 2015

[130] Zhang C, Zong H, Zhuge B, Lu X, Fang H, Zhu J (2016). Protoplast preparation and polyethylene glycol (PEG)-mediated transformation of Candida glycerinogenes. Biotechnol Bioproc E.

[131] Koiso N, Toda E, Ichikawa M, Kato N, Okamoto T (2017). Development of gene expression system in egg cells and zygotes isolated from rice and maize. Plant Direct.

[132] Osakabe Y, Liang Z, Ren C, Nishitani C, Osakabe K, Wada M (2018). CRISPR-Cas9-mediated genome editing in apple and grapevine. Nat Protoc.

[133] Woo JW, Kim J, Kwon SI, Corvalán C, Cho SW, Kim H (2015). DNA-free genome editing in plants with preassembled CRISPR-Cas9 ribonucleoproteins. Nat Biotechnol.

[134] Subburaj S, Chung SJ, Lee C, Ryu SM, Kim DH, Kim JS (2016). Site-directed mutagenesis in Petunia x hybrida protoplast system using direct delivery of purified recombinant Cas9 ribonucleoproteins. Plant Cell Rep.

[135] Malnoy M, Viola R, Jung MH, Koo OJ, Kim S, Kim JS (2016). DNA-free genetically edited grapevine and apple protoplast using CRISPR/Cas9 ribonucleoproteins. Front Plant Sci.

[136] Andersson M, Turesson H, Olsson N, Falt AS, Ohlsson P, Gonzalez MN (2018). Genome editing in potato via CRISPR-Cas9 ribonucleoprotein delivery. Physiol Plant.

[137] Hooghvorst I, Lopez-Cristoffanini C, Nogues S (2019). Efficient knockout of phytoene desaturase gene using CRISPR/Cas9 in melon. Sci Rep.

[138] Leisen T, Bietz F, Werner J, Wegner A, Schaffrath U, Scheuring D (2020). CRISPR/Cas with ribonucleoprotein complexes and transiently selected telomere vectors allows highly efficient marker-free and multiple genome editing in *Botrytis cinerea*. PLoS Pathog.

[139] Blechert O, Mei H, Zang X, Zheng H, Liang G, Liu W (2020). Auxotrophic mutations of Trichophyton rubrum created by in vitro synthesized Cas9 ribonucleoprotein. BMC Biotechnol.

[140] González MN, Massa GA, Andersson M, Turesson H, Olsson N, Fält AS (2020). Reduced enzymatic browning in potato tubers by specific editing of a polyphenol oxidase gene via ribonucleoprotein complexes delivery of the CRISPR/Cas9 system. Front Plant Sci.

[141] Fan Y, Xin S, Dai X, Yang X, Huang H, Hua Y (2020). Efficient genome editing of rubber tree (hevea brasiliensis) protoplasts using CRISPR/Cas9 ribonucleoproteins. Ind Crops Prod.

[142] Toda E, Koiso N, Takebayashi A, Ichikawa M, Kiba T, Osakabe K (2019). An efficient DNA- and selectable-marker-free genome-editing system using zygotes in rice. Nat Plants.

[143] Choi JG, Dang Y, Abraham S, Ma H, Zhang J, Guo H (2016). Lentivirus pre-packed with Cas9 protein for safer gene editing. Gene Ther.

[144] Mangeot PE, Risson V, Fusil F, Marnef A, Laurent E, Blin J (2019). Genome editing in primary cells and in vivo using viral-derived Nanoblades loaded with Cas9-sgRNA ribonucleoproteins. Nat Commun.

[145] Lyu P, Javidi-Parsijani P, Atala A, Lu B (2019). Delivering Cas9/sgRNA ribonucleoprotein (RNP) by lentiviral capsid-based bionanoparticles for efficient 'hit-and-run' genome editing. Nucleic Acids Res.

[146] Raposo G, Stoorvogel W (2013). Extracellular vesicles: Exosomes, microvesicles, and friends. J Cell Biol.

[147] El Andaloussi S, Mäger I, Breakefield XO, Wood MJA (2013). Extracellular vesicles: Biology and emerging therapeutic opportunities. Nat Rev Drug Discovery.

[148] Zappulli V, Pagh Friis K, Fitzpatrick Z, Maguire CA, Breakefield XO (2016). Extracellular vesicles and intercellular communication within the nervous system. J Clin Invest.

[149] Wang Q, Yu J, Kadungure T, Beyene J, Zhang H, Lu Q (2018). ARMMs as a versatile platform for intracellular delivery of macromolecules. Nat Commun.

[150] Nabhan JF, Hu R, Oh RS, Cohen SN, Lu Q (2012). Formation and release of arrestin domain-containing protein 1-mediated microvesicles (ARMMs) at plasma membrane by recruitment of TSG101 protein. Proc Natl Acad Sci U S A.

[151] Ye Y, Zhang X, Xie F, Xu B, Xie P, Yang T (2020). An engineered exosome for delivering sgRNA:Cas9 ribonucleoprotein complex and genome editing in recipient cells. Biomater Sci.

[152] Mangeot PE, Dollet S, Girard M, Ciancia C, Joly S, Peschanski M (2011). Protein transfer into human cells by VSV-G-induced nanovesicles. Mol Ther.

[153] Montagna C, Petris G, Casini A, Maule G, Franceschini GM, Zanella I (2018). VSV-G-enveloped vesicles for traceless delivery of CRISPR-Cas9. Mol Ther - Nucleic Acids.

[154] Campbell LA, Coke LM, Richie CT, Fortuno LV, Park AY, Harvey BK (2019). Gesicle-mediated delivery of CRISPR/Cas9 ribonucleoprotein complex for inactivating the HIV provirus. Mol Ther.

[155] Gee P, Lung MSY, Okuzaki Y, Sasakawa N, Iguchi T, Makita Y (2020). Extracellular nanovesicles for packaging of CRISPR-Cas9 protein and sgRNA to induce therapeutic exon skipping. Nat Commun.

[156] Li W, Szoka FC (2007). Lipid-based nanoparticles for nucleic acid delivery. Pharm Res.

[157] Felgner PL, Ringold GM (1989). Cationic liposome-mediated transfection. Nature.

[158] El Ouahabi A, Thiry M, Pector V, Fuks R, Ruysschaert JM, Vandenbranden M (1997). The role of endosome destabilizing activity in the gene transfer process mediated by cationic lipids. FEBS Letters.

[159] Zhou X, Huang L (1994). DNA transfection mediated by cationic liposomes containing lipopolylysine: characterization and mechanism of action. Biochim Biophys Acta.

[160] Liang X, Potter J, Kumar S, Zou Y, Quintanilla R, Sridharan M (2015). Rapid and highly efficient mammalian cell engineering via Cas9 protein transfection. J Biotechnol.

[161] Liu W, Rudis MR, Cheplick MH, Millwood RJ, Yang JP, Ondzighi-Assoume CA (2020). Lipofection-mediated genome editing using DNA-free delivery of the Cas9/gRNA ribonucleoprotein into plant cells. Plant Cell Rep.

[162] Wang Y, Wang B, Xie H, Ren Q, Liu X, Li F (2019). Efficient human genome editing using SaCas9 ribonucleoprotein complexes. Biotechnol J.

[163] Kim K, Park SW, Kim JH, Lee SH, Kim D, Koo T (2017). Genome surgery using Cas9 ribonucleoproteins for the treatment of age-related macular degeneration. Genome Res.

[164] Aird EJ, Lovendahl KN, St (2018). Martin A, Harris RS, Gordon WR. Increasing Cas9-mediated homology-directed repair efficiency through covalent tethering of DNA repair template. Commun Biol.

[165] Savic N, Ringnalda FC, Berk C, Bargsten K, Hall J, Jinek M (2019). In vitro generation of CRISPR-Cas9 complexes with covalently bound repair templates for genome editing in mammalian cells. Bio Protoc.

[166] Yu X, Liang X, Xie H, Kumar S, Ravinder N, Potter J (2016). Improved delivery of Cas9 protein/gRNA complexes using lipofectamine CRISPRMAX. Biotechnol Lett.

[167] Haapaniemi E, Botla S, Persson J, Schmierer B, Taipale J (2018). CRISPR-Cas9 genome editing induces a p53-mediated DNA damage response. Nat Med.

[168] de Almeida Monteiro Melo Ferraz M, Nagashima JB, Venzac B, Le Gac S, Songsasen N (2020). A dog oviduct-on-a-chip model of serous tubal intraepithelial carcinoma. Sci Rep.

[169] Chin JS, Chooi WH, Wang H, Ong W, Leong KW, Chew SY (2019). Scaffold-mediated non-viral delivery platform for CRISPR/Cas9-based genome editing. Acta Biomater.

[170] Wang M, Zuris JA, Meng F, Rees H, Sun S, Deng P (2016). Efficient delivery of genome-editing proteins using bioreducible lipid nanoparticles. Proc Natl Acad Sci U S A.

[171] Li Y, Bolinger J, Yu Y, Glass Z, Shi N, Yang L (2019). Intracellular delivery and biodistribution study of CRISPR/Cas9 ribonucleoprotein loaded bioreducible lipidoid nanoparticles. Biomater Sci.

[172] Li Y, Yang T, Yu Y, Shi N, Yang L, Glass Z (2018). Combinatorial library of chalcogen-containing lipidoids for intracellular delivery of genome-editing proteins. Biomaterials.

[173] Li Y, Li AC, Xu Q (2019). Intracellular delivery of His-tagged genome-editing proteins enabled by nitrilotriacetic acid-containing lipidoid nanoparticles. Adv Healthc Mater.

[174] Cho EY, Ryu JY, Lee HAR, Hong SH, Park HS, Hong KS (2019). Lecithin nano-liposomal particle as a CRISPR/Cas9 complex delivery system for treating type 2 diabetes. J Nanobiotechnol.

[175] Kuhn J, Lin Y, Krhac Levacic A, Al Danaf N, Peng L, Höhn M (2020). Delivery of Cas9/sgRNA ribonucleoprotein complexes via hydroxystearyl oligoamino amides. Bioconjugate Chem.

[176] Li J, Røise JJ, Zhang J, Yang J, Kerr DL, Han H (2019). A novel fluorescent surfactant enhances the delivery of the Cas9 ribonucleoprotein and enables the identification of edited cells. Chem Commun.

[177] Ramakrishna S, Kwaku Dad AB, Beloor J, Gopalappa R, Lee SK, Kim H (2014). Gene disruption by cell-penetrating peptide-mediated delivery of Cas9 protein and guide RNA. Genome Res.

[178] Yin J, Wang Q, Hou S, Bao L, Yao W, Gao X (2018). Potent protein delivery into mammalian cells via a supercharged polypeptide. J Am Chem Soc.

[179] Yin J, Hou S, Wang Q, Bao L, Liu D, Yue Y (2019). Microenvironment-responsive delivery of the Cas9 RNA-guided endonuclease for efficient genome editing. Bioconjug Chem.

[180] Kim SM, Shin SC, Kim EE, Kim SH, Park K, Oh SJ (2018). Simple in vivo gene editing via direct self-assembly of Cas9 ribonucleoprotein complexes for cancer treatment. ACS Nano.

[181] Park YJ, Chang L-C, Liang JF, Moon C, Chung C-P, Yang VC (2005). Nontoxic membrane translocation peptide from protamine, low molecular weight protamine (LMWP), for enhanced intracellular protein delivery: in vitro and in vivo study. FASEB J.

[182] Ju A, Lee SW, Lee YE, Han KC, Kim JC, Shin SC (2019). A carrier-free multiplexed gene editing system applicable for suspension cells. Biomaterials.

[183] Shen Y, Cohen JL, Nicoloro SM, Kelly M, Yenilmez B, Henriques F (2018). CRISPR-delivery particles targeting nuclear receptor-interacting protein 1 (Nrip1) in adipose cells to enhance energy expenditure. J Bacteriol.

[184] Del'Guidice T, Lepetit-Stoffaes JP, Bordeleau LJ, Roberge J, Théberge V, Lauvaux C (2018). Membrane permeabilizing amphiphilic peptide delivers recombinant transcription factor and CRISPR-Cas9/Cpf1 ribonucleoproteins in hard-to-modify cells. PLoS One.

[185] Newcomb CJ, Sur S, Lee SS, Yu JM, Zhou Y, Snead ML (2016). Supramolecular nanofibers enhance growth factor signaling by increasing lipid raft mobility. Nano Lett.

[186] Ji T, Ding Y, Zhao Y, Wang J, Qin H, Liu X (2015). Peptide assembly integration of fibroblast-targeting and cell-penetration features for enhanced antitumor drug delivery. Adv Mater.

[187] Rong G, Wang C, Chen L, Yan Y, Cheng Y (2020). Fluoroalkylation promotes cytosolic peptide delivery. Sci Adv.

[188] Lostalé-Seijo I, Louzao I, Juanes M, Montenegro J (2017). Peptide/Cas9 nanostructures for ribonucleoprotein cell membrane transport and gene edition. Chem Sci.

[189] Thach TT, Bae DH, Kim NH, Kang ES, Lee BS, Han K (2019). Lipopeptide-based nanosome-mediated delivery of hyperaccurate CRISPR/Cas9 ribonucleoprotein for gene editing. Small.

[190] Chen JS, Dagdas YS, Kleinstiver BP, Welch MM, Sousa AA, Harrington LB (2017). Enhanced proofreading governs CRISPR-Cas9 targeting accuracy. Nature.

[191] Jain PK, Lo JH, Rananaware S, Downing M, Panda A, Tai M (2019). Non-viral delivery of CRISPR/Cas9 complex using CRISPR-GPS nanocomplexes. Nanoscale.

[192] Wang M, Liu H, Li L, Cheng Y (2014). A fluorinated dendrimer achieves excellent gene transfection efficacy at extremely low nitrogen to phosphorus ratios. Nat Commun.

[193] Srivastava A, Yadav T, Sharma S, Nayak A, Akanksha Kumari A, Mishra N (2016). Polymers in drug delivery. J Biosci Med.

[194] Lv J, Fan Q, Wang H, Cheng Y (2019). Polymers for cytosolic protein delivery. Biomaterials.

[195] Sun M, Wang K, Oupicky D (2018). Advances in stimulus-responsive polymeric materials for systemic delivery of nucleic acids. Adv Healthc Mater.

[196] Shen W, Wang R, Fan Q, Gao X, Wang H, Shen Y (2020). Natural polyphenol inspired polycatechols for efficient siRNA delivery. CCS Chem.

[197] Zhang Z, Shen W, Ling J, Yan Y, Hu J, Cheng Y (2018). The fluorination effect of fluoroamphiphiles in cytosolic protein delivery. Nat Commun.

[198] Li G, Yuan S, Deng D, Ou T, Li Y, Sun R (2019). Fluorinated polyethylenimine to enable transmucosal delivery of photosensitizer-conjugated catalase for photodynamic therapy of orthotopic bladder tumors postintravesical instillation. Adv Func Mater.

[199] Shen W, Wang Q, Shen Y, Gao X, Li L, Yan Y (2018). Green tea catechin dramatically promotes RNAi mediated by low-molecular-weight polymers. ACS Cent Sci.

[200] Lv J, Tan E, Wang Y, Fan Q, Yu J, Cheng Y (2020). Tailoring guanidyl-rich polymers for efficient cytosolic protein delivery. J Control Release.

[201] Yang J, Zhang Q, Chang H, Cheng Y (2015). Surface-engineered dendrimers in gene delivery. Chem Rev.

[202] Li T-F, Cheng Y-Y, Wang Y, Wang H, Chen D-F, Liu Y-T (2019). Analysis of dimer impurity in polyamidoamine dendrimer solutions by small-angle neutron scattering. Chin J Polym Sci.

[203] Zhao L, Wu Q, Cheng Y, Zhang J, Wu J, Xu T (2010). High-throughput screening of dendrimer-binding drugs. J Am Chem Soc.

[204] Wang H, Wang Y, Wang Y, Hu J, Li T, Liu H (2015). Self-assembled fluorodendrimers combine the features of lipid and polymeric vectors in gene delivery. Angew Chem Int Ed.

[205] Ren L, Lv J, Wang H, Cheng Y (2020). A coordinative dendrimer achieves excellent efficiency in cytosolic protein and peptide delivery. Angew Chem Int Ed.

[206] Chang H, Lv J, Gao X, Wang X, Wang H, Chen H (2017). Rational design of a polymer with robust efficacy for intracellular protein and peptide delivery. Nano Lett.

[207] Lv S, Wu Y, Cai K, He H, Li Y, Lan M (2018). High drug loading and sub-quantitative loading efficiency of polymeric micelles driven by donor-receptor coordination interactions. J Am Chem Soc.

[208] Lv J, Liu C, Lv K, Wang H, Cheng Y (2020). Boronic acid-rich dendrimer for efficient intracellular peptide delivery. Sci Chin Mater.

[209] Taharabaru T, Yokoyama R, Higashi T, Mohammed AFA, Inoue M, Maeda Y (2020). Genome editing in a wide area of the brain using dendrimer-based ternary polyplexes of Cas9 ribonucleoprotein. ACS Appl Mater Interfaces.

[210] Gao Y, Huang J-Y, O'Keeffe Ahern J, Cutlar L, Zhou D, Lin F-H (2016). Highly branched poly(β-amino esters) for non-viral gene delivery: High transfection efficiency and low toxicity achieved by increasing molecular weight. Biomacromolecules.

[211] Rui Y, Wilson DR, Sanders K, Green JJ (2019). Reducible branched ester-amine quadpolymers (rBEAQs) codelivering plasmid DNA and RNA oligonucleotides enable CRISPR/Cas9 genome editing. ACS Appl Mater Interfaces.

[212] Rui Y, Wilson DR, Choi J, Varanasi M, Sanders K, Karlsson J (2019). Carboxylated branched poly(β-amino ester) nanoparticles enable robust cytosolic protein delivery and CRISPR-Cas9 gene editing. Sci Adv.

[213] Vlassi E, Papagiannopoulos A, Pispas S (2017). Self-assembly of poly(ethylene glycol-b-phenyl oxazoline) diblock copolymers in aqueous media and their interactions with proteins. Colloid Polym Sci.

[214] Izaki S, Kurinomaru T, Handa K, Kimoto T, Shiraki K (2015). Stress tolerance of antibody-poly(amino acid) complexes for improving the stability of high concentration antibody formulations. J Pharm Sci.

[215] Goycoolea FM, Valle-Gallego A, Stefani R, Menchicchi B, David L, Rochas C (2012). Chitosan-based nanocapsules: physical characterization, stability in biological media and capsaicin encapsulation. Colloid Polym Sci.

[216] Bagre AP, Jain K, Jain NK (2013). Alginate coated chitosan core shell nanoparticles for oral delivery of enoxaparin: in vitro and in vivo assessment. Int J Pharm.

[217] Schubert J, Chanana M (2018). Coating matters: Review on colloidal stability of nanoparticles with biocompatible coatings in biological media, living cells and organisms. Curr Med Chem.

[218] Liu Q, Cai J, Zheng Y, Tan Y, Wang Y, Zhang Z (2019). NanoRNP overcomes tumor heterogeneity in cancer treatment. Nano Lett.

[219] Qiao J, Sun W, Lin S, Jin R, Ma L, Liu Y (2019). Cytosolic delivery of CRISPR/Cas9 ribonucleoproteins for genome editing using chitosan-coated red fluorescent protein. Chem Commun.

[220] Wan T, Chen Y, Pan Q, Xu X, Kang Y, Gao X (2020). Genome editing of mutant KRAS through supramolecular polymer-mediated delivery of Cas9 ribonucleoprotein for colorectal cancer therapy. J Controlled Release.

[221] Chen G, Ma B, Wang Y, Gong S (2018). A universal GSH-responsive nanoplatform for the delivery of DNA, mRNA, and Cas9/sgRNA ribonucleoprotein. ACS Appl Mater Interfaces.

[222] Wang Y, Ma B, Abdeen AA, Chen G, Xie R, Saha K (2018). Versatile redox-responsive polyplexes for the delivery of plasmid DNA, messenger RNA, and CRISPR-Cas9 genome-editing machinery. ACS Appl Mater Interfaces.

[223] Chen G, Abdeen AA, Wang Y, Shahi PK, Robertson S, Xie R (2019). A biodegradable nanocapsule delivers a Cas9 ribonucleoprotein complex for in vivo genome editing. Nat Nanotechnol.

[224] Shah S, Rangaraj N, Laxmikeshav K, Sampathi S (2020). "Nanogels as drug carriers - Introduction, chemical aspects, release mechanisms and potential applications". Int J Pharm.

[225] Yin Y, Hu B, Yuan X, Cai L, Gao H, Yang Q (2020). Nanogel: A versatile nano-delivery system for biomedical applications. Pharmaceutics.

[226] Ding F, Huang X, Gao X, Xie M, Pan G, Li Q (2019). A non-cationic nucleic acid nanogel for the delivery of the CRISPR/Cas9 gene editing tool. Nanoscale.

[227] Mout R, Ray M, Tay T, Sasaki K, Yesilbag Tonga G, Rotello VM (2017). General strategy for direct cytosolic protein delivery via protein-nanoparticle co-engineering. ACS Nano.

[228] Jiang Y, Hardie J, Liu Y, Ray M, Luo X, Das R (2018). Nanocapsule-mediated cytosolic siRNA delivery for anti-inflammatory treatment. J Control Release.

[229] Mout R, Rotello VM (2017). Cytosolic and nuclear delivery of CRISPR/Cas9-ribonucleoprotein for gene editing using arginine functionalized gold nanoparticles. Bio Protoc.

[230] Ray M, Lee YW, Hardie J, Mout R, Yeşilbag Tonga G, Farkas ME (2018). CRISPRed macrophages for cell-based cancer immunotherapy. Bioconjug Chem.

[231] Wang P, Zhang L, Xie Y, Wang N, Tang R, Zheng W (2017). Genome editing for cancer therapy: Delivery of Cas9 protein/sgRNA plasmid via a gold nanocluster/lipid core-shell nanocarrier. Adv Sci.

[232] Wang P, Zhang L, Zheng W, Cong L, Guo Z, Xie Y (2018). Thermo-triggered release of CRISPR-Cas9 system by lipid-encapsulated gold nanoparticles for tumor therapy. Angew Chem Int Ed Engl.

[233] Ju E, Li T, Ramos da Silva S, Gao S-J (2019). Gold nanocluster-mediated efficient delivery of Cas9 protein through pH-induced assembly-disassembly for inactivation of virus oncogenes. ACS Appl Mater Interfaces.

[234] Prigodich AE, Randeria PS, Briley WE, Kim NJ, Daniel WL, Giljohann DA (2012). Multiplexed nanoflares: mRNA detection in live cells. Anal Chem.

[235] Zheng D, Giljohann DA, Chen DL, Massich MD, Wang XQ, Iordanov H (2012). Topical delivery of siRNA-based spherical nucleic acid nanoparticle conjugates for gene regulation. Proc Natl Acad Sci U S A.

[236] Young KL, Scott AW, Hao L, Mirkin SE, Liu G, Mirkin CA (2012). Hollow spherical nucleic acids for intracellular gene regulation based upon biocompatible silica shells. Nano Lett.

[237] Li TI, Sknepnek R, Macfarlane RJ, Mirkin CA, de la Cruz MO (2012). Modeling the crystallization of spherical nucleic acid nanoparticle conjugates with molecular dynamics simulations. Nano Lett.

[238] Jensen SA, Day ES, Ko CH, Hurley LA, Luciano JP, Kouri FM (2013). Spherical nucleic acid nanoparticle conjugates as an RNAi-based therapy for glioblastoma. Sci Transl Med.

[239] Briley WE, Bondy MH, Randeria PS, Dupper TJ, Mirkin CA (2015). Quantification and real-time tracking of RNA in live cells using Sticky-flares. Proc Natl Acad Sci U S A.

[240] Randeria PS, Seeger MA, Wang XQ, Wilson H, Shipp D, Mirkin CA (2015). siRNA-based spherical nucleic acids reverse impaired wound healing in diabetic mice by ganglioside GM3 synthase knockdown. Proc Natl Acad Sci U S A.

[241] Barnaby SN, Perelman GA, Kohlstedt KL, Chinen AB, Schatz GC, Mirkin CA (2016). Design considerations for RNA spherical nucleic acids (SNAs). Bioconjug Chem.

[242] Tommasini-Ghelfi S, Lee A, Mirkin CA, Stegh AH (2019). Synthesis, physicochemical, and biological evaluation of spherical nucleic acids for RNAi-based therapy in glioblastoma. Methods Mol Biol.

[243] Guan CM, Chinen AB, Ferrer JR, Ko CH, Mirkin CA (2019). Impact of sequence specificity of spherical nucleic acids on macrophage activation in vitro and in vivo. Mol Pharm.

[244] Lee K, Conboy M, Park HM, Jiang F, Kim HJ, Dewitt MA (2017). Nanoparticle delivery of Cas9 ribonucleoprotein and donor DNA in vivo induces homology-directed DNA repair. Nat Biomed Eng.

[245] Lee B, Lee K, Panda S, Gonzales-Rojas R, Chong A, Bugay V (2018). Nanoparticle delivery of CRISPR into the brain rescues a mouse model of fragile X syndrome from exaggerated repetitive behaviours. Nat Biomed Eng.

[246] Shahbazi R, Sghia-Hughes G, Reid JL, Kubek S, Haworth KG, Humbert O (2019). Targeted homology-directed repair in blood stem and progenitor cells with CRISPR nanoformulations. Nat Mater.

[247] James SL (2003). Metal-organic frameworks. Chem Soc Rev.

[248] Hayashi H, Côté AP, Furukawa H, O'Keeffe M, Yaghi OM (2007). Zeolite A imidazolate frameworks. Nat Mater.

[249] Alsaiari SK, Patil S, Alyami M, Alamoudi KO, Aleisa FA, Merzaban JS (2018). Endosomal escape and delivery of CRISPR/Cas9 genome editing machinery enabled by nanoscale zeolitic imidazolate framework. J Am Chem Soc.

[250] Alyami MZ, Alsaiari SK, Li Y, Qutub SS, Aleisa FA, Sougrat R (2020). Cell-type-specific CRISPR/Cas9 delivery by biomimetic metal organic frameworks. J Am Chem Soc.

[251] Yang X, Tang Q, Jiang Y, Zhang M, Wang M, Mao L (2019). Nanoscale ATP-responsive zeolitic imidazole framework-90 as a general platform for cytosolic protein delivery and genome editing. J Am Chem Soc.

[252] Wang Y, Shahi PK, Xie R, Zhang H, Abdeen AA, Yodsanit N (2020). A pH-responsive silica-metal-organic framework hybrid nanoparticle for the delivery of hydrophilic drugs, nucleic acids, and CRISPR-Cas9 genome-editing machineries. J Controlled Release.

[253] Dreyer DR, Park S, Bielawski CW, Ruoff RS (2010). The chemistry of graphene oxide. Chem Soc Rev.

[254] Huang P, Xu C, Lin J, Wang C, Wang X, Zhang C (2011). Folic acid-conjugated graphene oxide loaded with photosensitizers for targeting photodynamic therapy. Theranostics.

[255] Shen H, Zhang L, Liu M, Zhang Z (2012). Biomedical applications of graphene. Theranostics.

[256] Yue H, Zhou X, Cheng M, Xing D (2018). Graphene oxide-mediated Cas9/sgRNA delivery for efficient genome editing. Nanoscale.

[257] Qu G, Liu W, Zhao Y, Gao J, Xia T, Shi J (2017). Improved biocompatibility of black Phosphorus nanosheets by chemical modification. Angew Chem Int Ed.

[258] Kim DW, Jeong HS, Kwon KO, Ok JM, Kim SM, Jung H-T (2016). Ultrastrong anchoring on the periodic atomic grooves of black phosphorus. Adv Mater Interfaces.

[259] Eswaraiah V, Zeng Q, Long Y, Liu Z (2016). Black phosphorus nanosheets: Synthesis, characterization and applications. Small.

[260] Zhou W, Cui H, Ying L, Yu XF (2018). Enhanced cytosolic delivery and release of CRISPR/Cas9 by black phosphorus nanosheets for genome editing. Angew Chem Int Ed.

[261] Levingstone TJ, Herbaj S, Redmond J, McCarthy HO, Dunne NJ (2020). Calcium phosphate nanoparticles-based systems for RNAi delivery: Applications in bone tissue regeneration. Nanomaterials (Basel).

[262] Levingstone TJ, Herbaj S, Dunne NJ (2019). Calcium phosphate nanoparticles for therapeutic applications in bone regeneration. Nanomaterials (Basel).

[263] Li S, Song Z, Liu C, Chen XL, Han H (2019). Biomimetic mineralization-based CRISPR/Cas9 ribonucleoprotein nanoparticles for gene editing. ACS Appl Mater Interfaces.

[264] Sun W, Jiang T, Lu Y, Reiff M, Mo R, Gu Z (2014). Cocoon-like self-degradable DNA nanoclew for anticancer drug delivery. J Am Chem Soc.

[265] Ruan W, Zheng M, An Y, Liu Y, Lovejoy DB, Hao M (2018). DNA nanoclew templated spherical nucleic acids for siRNA delivery. Chem Commun.

[266] Sun W, Ji W, Hall JM, Hu Q, Wang C, Beisel CL (2015). Self-assembled DNA nanoclews for the efficient delivery of CRISPR-Cas9 for genome editing. Angew Chem Int Ed.

[267] Wujin S, Jinqiang W, Quanyin H, Xingwu Z, Ali K, Zhen G (2020). CRISPR-Cas12a delivery by DNA-mediated bioresponsive editing for cholesterol regulation. Sci Adv.

[268] Ha JS, Lee JS, Jeong J, Kim H, Byun J, Kim SA (2017). Poly-sgRNA/siRNA ribonucleoprotein nanoparticles for targeted gene disruption. J Controlled Release.

[269] Zhou W, Brown W, Bardhan A, Delaney M, Ilk A, Rauen R (2020). Spatiotemporal control of CRISPR/Cas9 function in cells and zebrafish using light-activated guide RNA. Angew Chem Int Ed.

[270] Chen G, Qiu H, Prasad PN, Chen X (2014). Upconversion nanoparticles: Design, nanochemistry, and applications in theranostics. Chem Rev.

[271] Haase M, Schäfer H (2011). Upconverting nanoparticles. Angew Chem Int Ed Engl.

[272] Pan Y, Yang J, Luan X, Liu X, Li X, Yang J (2019). Near-infrared upconversion-activated CRISPR-Cas9 system: A remote-controlled gene editing platform. Sci Adv.

[273] Ryu JY, Won EJ, Lee HAR, Kim JH, Hui E, Kim HP (2020). Ultrasound-activated particles as CRISPR/Cas9 delivery system for androgenic alopecia therapy. Biomaterials.

[274] Kocak G, Tuncer C, Bütün V (2017). pH-Responsive polymers. Polym Chem.

[275] Pei D, Buyanova M (2019). Overcoming endosomal entrapment in drug delivery. Bioconjugate Chem.

[276] Rouet R, Thuma BA, Roy MD, Lintner NG, Rubitski DM, Finley JE (2018). Receptor-mediated delivery of CRISPR-Cas9 endonuclease for cell-type-specific gene editing. J Am Chem Soc.

[277] Rouet R, Christ D (2019). Efficient intracellular delivery of CRISPR-Cas ribonucleoproteins through receptor mediated endocytosis. ACS Chem Biol.

[278] Zhang L, Wang L, Xie Y, Wang P, Deng S, Qin A (2019). Triple-targeting delivery of CRISPR/Cas9 to reduce the risk of cardiovascular diseases. Angew Chem Int Ed.

[279] Chen Z, Liu F, Chen Y, Liu J, Wang X, Chen AT (2017). Targeted delivery of CRISPR/Cas9-mediated cancer gene therapy via liposome-templated hydrogel nanoparticles. Adv Funct Mater.

[280] He X, Long Q, Zeng Z, Yang L, Tang Y, Feng X (2019). Simple and efficient targeted intracellular protein delivery with self-assembled nanovehicles for effective cancer therapy. Adv Funct Mater.

[281] Cheng Q, Wei T, Farbiak L, Johnson LT, Dilliard SA, Siegwart DJ (2020). Selective organ targeting (SORT) nanoparticles for tissue-specific mRNA delivery and CRISPR-Cas gene editing. Nat. Nanotech.

[282] Chaterji S, Ahn EH, Kim DH (2017). CRISPR genome engineering for human pluripotent stem cell research. Theranostics.

[283] Wu SS, Li QC, Yin CQ, Xue W, Song CQ (2020). Advances in CRISPR/Cas-based gene therapy in human genetic diseases. Theranostics.

[284] Carlson-Stevermer J, Abdeen AA, Kohlenberg L, Goedland M, Molugu K, Lou M (2017). Assembly of CRISPR ribonucleoproteins with biotinylated oligonucleotides via an RNA aptamer for precise gene editing. Nat Commun.

[285] Varkouhi AK, Scholte M, Storm G, Haisma HJ (2011). Endosomal escape pathways for delivery of biologicals. J Control Release.

[286] Smith SA, Selby LI, Johnston APR, Such GK (2019). The endosomal escape of nanoparticles: Toward more efficient cellular delivery. Bioconjug Chem.

[287] Ahmad A, Khan JM, Haque S (2019). Strategies in the design of endosomolytic agents for facilitating endosomal escape in nanoparticles. Biochimie.

